# Total Synthesis of Putative 11-*epi*-Lyngbouilloside Aglycon

**DOI:** 10.3389/fchem.2016.00034

**Published:** 2016-08-09

**Authors:** Amandine Kolleth, Julian Gebauer, Abdelatif ElMarrouni, Raphael Lebeuf, Céline Prévost, Eric Brohan, Stellios Arseniyadis, Janine Cossy

**Affiliations:** ^1^Laboratoire de Chimie Organique, Institute of Chemistry, Biology and Innovation, ESPCI Paris, Centre National de la Recherche Scientifique (UMR8231), PSL Research UniversityParis, France; ^2^LGCR-Analytical Sciences, SanofiVitry-sur-Seine, France

**Keywords:** lyngbouilloside, total synthesis, *Lyngbya bouillonii*, Boeckman esterification, Mukaiyama aldol, asymmetric Sharpless dihydroxylation, ring-closing metathesis

## Abstract

We report here the total synthesis of 11-*epi*-lyngbouilloside aglycon. Our strategy features a Boeckman-type esterification followed by a RCM to form the 14-membered ring macrolactone and a late-stage side chain introduction via a Wittig olefination. Overall, the final product was obtained in 20 steps and 2% overall yield starting from commercially available 3-methyl-but-3-enol. Most importantly, the strategy employed is versatile enough to eventually allow us to complete the synthesis of the natural product and irrevocably confirm its structure.

## Introduction

**Figure 1:**
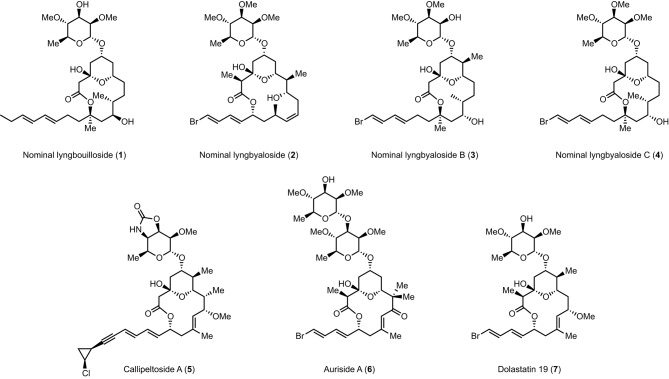
**Structures of lyngbouilloside, lyngbyaloside, lyngbyaloside B, lyngbyaloside C, callipeltoside A, auriside A, and dolastatin 19**.

Lyngbouilloside (**1**) is a glycosidic macrolide isolated by Gerwick et al. (Tan et al., 2002) from the cyanobacteria *Lyngbya bouillonii* (Hoffmann and Demoulin, 1991), which also produce several other structurally intriguing natural products including the tetrapeptide lyngbyapeptin (Klein et al., 1999a,b), several macrolides such as laingolide, laingolide A, and madangolide (Klein et al., 1996, 1999a,b), and various lyngbouilloside analogs such as lyngbyaloside (**2**) (Klein et al., 1997), lyngbyaloside B (**3**) (Luesch et al., 2002; Matthew et al., 2010), and lyngbyaloside C (**4**) (Matthew et al., 2010; Figure 1). The structure of lyngbouilloside was determined after exhaustive 1D and 2D NMR analysis, HR-FABMS, IR, and UV absorption experiments, which unveiled the presence of the pendant dienyl side chain, the 14-membered ring lactone, the presence of hydroxyl groups, the chair conformation of the tetrahydropyran ring and the relative configuration of the stereogenic centers in the aglycon portion of the natural product. The nature of the sugar, on the other hand, was assigned by correlations in the ^1^H-^1^H COSY and HMBC spectral data and comparison with the sugar unit present in auriside A. Interestingly, lyngbouilloside exhibits only a moderate cytotoxic activity (IC_50_ = 17 μM) toward neuroblastoma cell lines. Nonetheless, its structural resemblance with several biologically active 14-membered macrolides, such as callipeltoside A (**5**), auriside A (**6**), or dolastatin 19 (**7**), encouraged a few groups including ours to complete its synthesis (Gebauer et al., 2008; Webb et al., 2009; ElMarrouni et al., 2012; Sabitha et al., 2014). In this context, we recently reported the total synthesis of nominal lyngbouilloside aglycone *via* a flexible approach featuring an acyl ketene macrolactonization and a late stage side chain introduction, which led us to suggest a stereochemical reassignment at C11. With this hypothesis in mind, we embarked on the synthesis of putative 11-*epi*-lyngbouilloside aglycon; we report here the results of our endeavor.

## Materials and methods

Experimental procedures and compound characterization data are furnished in the Supplementary Material.

## Results and discussion

**Figure 2:**
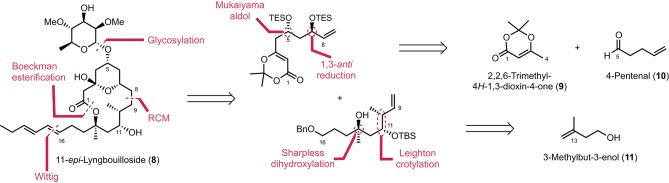
**Retrosynthetic analysis of 11-*epi*-lyngbouilloside**.

Our initial route to 11-*epi*-lyngbouilloside **8** relied on the same acyl ketene macrolactonization and Wittig olefination that were previously used to complete the synthesis of the proposed structure of lyngbouilloside aglycone. Unfortunately, the poor yields obtained in the macrolactonization process, combined with the difficulties encountered while trying to selectively reduce the C8–C9 double bond in the presence of the pendant alkyne side chain, led us to reconsider our strategy. We therefore opted for a slightly modified route, which involved a Boeckman-type esterification between an alcohol and an acyl ketene (Boeckman and Pruitt, 1989) and a ring-closing metathesis to form the 14-membered ring macrolactone, while a pendant hydroxyl group was placed instead of an alkynyl group in order to introduce the dienyl side-chain *via* a stereoselective Wittig reaction (Figure 2). We projected to control the stereogenic centers at C7 and C13 *via* a Sharpless dihydroxylation (Jacobsen et al., 1988; Kolb et al., 1994) and a 1,3-*anti* reduction respectively, while the C10 and C11 stereogenic centers were to be controlled through a Leighton type crotylation.

**Scheme 1:**
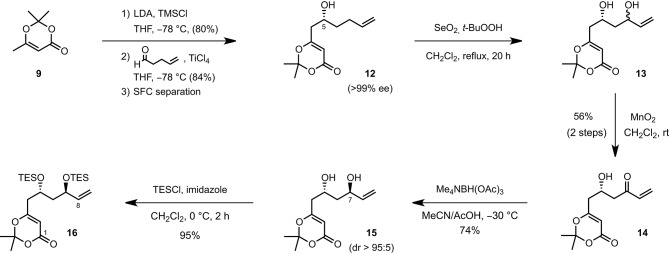
**Synthetis of the C1–C8 fragment**.

The synthesis of 11-*epi*-lyngbouilloside **8** began by first converting 2,2,6-trimethyl-4*H*-1,3-dioxin-4-one **9** to the corresponding silyl dienol ether (LDA, TMSCl, THF, −78°C) and subjecting the latter to 4-pentenal under asymmetric vinylogous aldol conditions (Denmark et al., 2005a,b). Among the various enantioselective catalytic processes developed so far in the field of asymmetric Mukaiyama aldol, the ones reported by Denmark et al. (Denmark et al., 2002, 2005a,b; Denmark and Beutner, 2003), involving the combination of a catalytic amount of chiral bis-phosphoramide and silicon tetrachloride to promote a highly enantio- and diastereoselective addition of silyl ketene acetals to aldehydes (SiCl_4_, CH_2_Cl_2_, −78°C), appeared particularly attractive. Unfortunately, the application of these conditions to our system afforded the desired product **12** in a modest 65% ee. The conditions reported by Sato [Ti(O*i*-Pr)_4_ (20 mol%), (*S*)-BINOL (20 mol%), THF, −78°C; Sato et al., 1995] and more recently by Scettri [Ti(O*i*-Pr)_4_ (8 mol%), (*S*)-BINOL (8 mol%), and 2 equiv of silyl dienol ether instead of 1.4 equiv, THF, −78°C; De Rosa et al., 2003] were also tested but afforded compound **12** in moderate yields albeit in up to 86% ee. With these rather disappointing results in hand, we decide to perform the aldol reaction in a racemic fashion (TiCl_4_, THF, −78°C) and separate the racemate by chiral preparative supercritical fluid chromatography (SFC) (Scheme 1). This preparative separation allowed to readily obtain large quantities of alcohol **12** in optically pure form (>99% ee) and with an acceptable overall yield of 34%. The absolute configuration was secured after hydrogenating the terminal double bond and comparing the optical rotation of the resulting product {${[\alpha ]}_{\text{D}}^{20}$ −21.0 (c 0.1, CHCl_3_)} with the one reported in the literature {${[\alpha ]}_{\text{D}}^{20}$ +19.0 (CHCl_3_)} (Sato et al., 1995). To complete the synthesis of the C1-C8 fragment, alcohol **12** was eventually treated with SeO_2_ and *t*-BuOOH (CH_2_Cl_2_, rt) to afford the corresponding diol, which was subsequently engaged in a MnO_2_-mediated oxidation to provide the desired enone **14** in 56% overall yield. A diastereoselective *anti*-reduction [Me_4_NBH(OAc)_3_, MeCN/AcOH, −30°C; Evans et al., 1988] followed by the protection of the resulting diol as a bis(triethylsilyl) ether (TESCl, imidazole, CH_2_Cl_2_, 0°C) finally provided the C1–C8 fragment **16** in six steps and 14% overall yield starting from the inexpensive dioxolenone **9**.

**Scheme 2:**
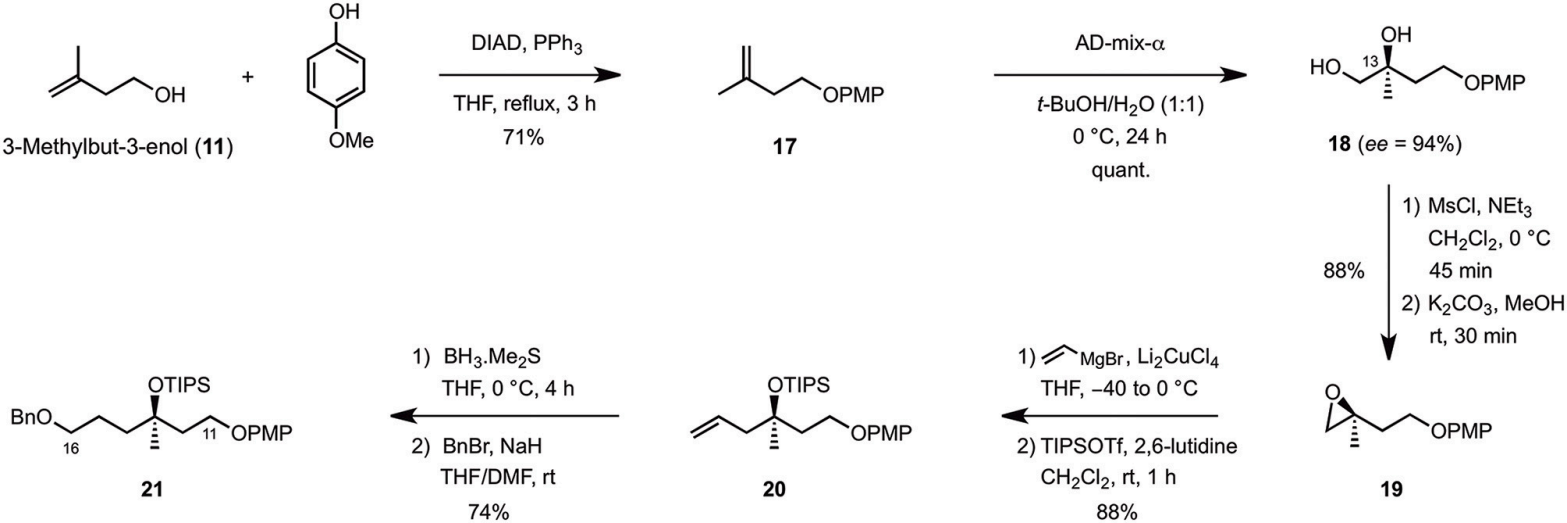
**Synthetis of the C11–C16 fragment**.

The synthesis of the C9–C16 fragment started off from commercially available 3-methylbuten-3-enol (**11**), which was first protected as its PMP-ether under Mitsunobu conditions (DIAD, PPh_3_, THF reflux) (Mitsunobu and Yamada, 1967) before it was engaged in the asymmetric Sharpless dihydroxylation (AD-mix-α, *t*-BuOH/H_2_O, 0°C) to quantitatively afford diol **17** in 94% ee (Scheme 2). Mesylation (MsCl, Et_3_N, CH_2_Cl_2_, 0°C) and cyclization under basic conditions (K_2_CO_3_, MeOH) then yielded epoxide **19** which, after Cu-catalyzed ring-opening using vinyl magnesium bromide (Li_2_CuCl_4_, THF, −40 to 0°C) and TIPS-protection (TIPSOTf, 2,6-lutidine, CH_2_Cl_2_, rt), produced the corresponding homoallylic ether **20** in 88% overall yield. Finally, hydroboration of the terminal double bond (BH_3_·Me_2_S, THF, 0°C) and benzylation of the primary alcohol obtained upon oxidative workup (BnBr, NaH, THF/DMF, rt) gave rise to the C11–C16 fragment **21** in 74% yield over two steps.

**Scheme 3:**
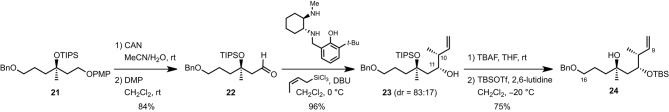
**Synthetis of the C9–C16 fragment**.

To control the two stereogenic centers at C10 and C12 and complete the synthesis of the C9–C16 fragment, we performed a *syn*-crotylation of aldehyde **22** obtained upon sequential PMP-deprotection (CAN, MeCN/H_2_O, rt)/oxidation [DMP, CH_2_Cl_2_, rt] using a procedure recently developed by Leighton and co-workers (Kim et al., 2011) (Scheme 3). This almost quantitatively afforded a mixture of the two diastereoisomeric homoallylic alcohols **23** (dr = 83:17), which could be converted to the desired C9–C16 fragment **24** by simple protecting group manipulation (TBAF, THF, rt, then TBSOTf, 2,6-lutidine, CH_2_Cl_2_, −40°C) in 75% yield.

**Scheme 4:**
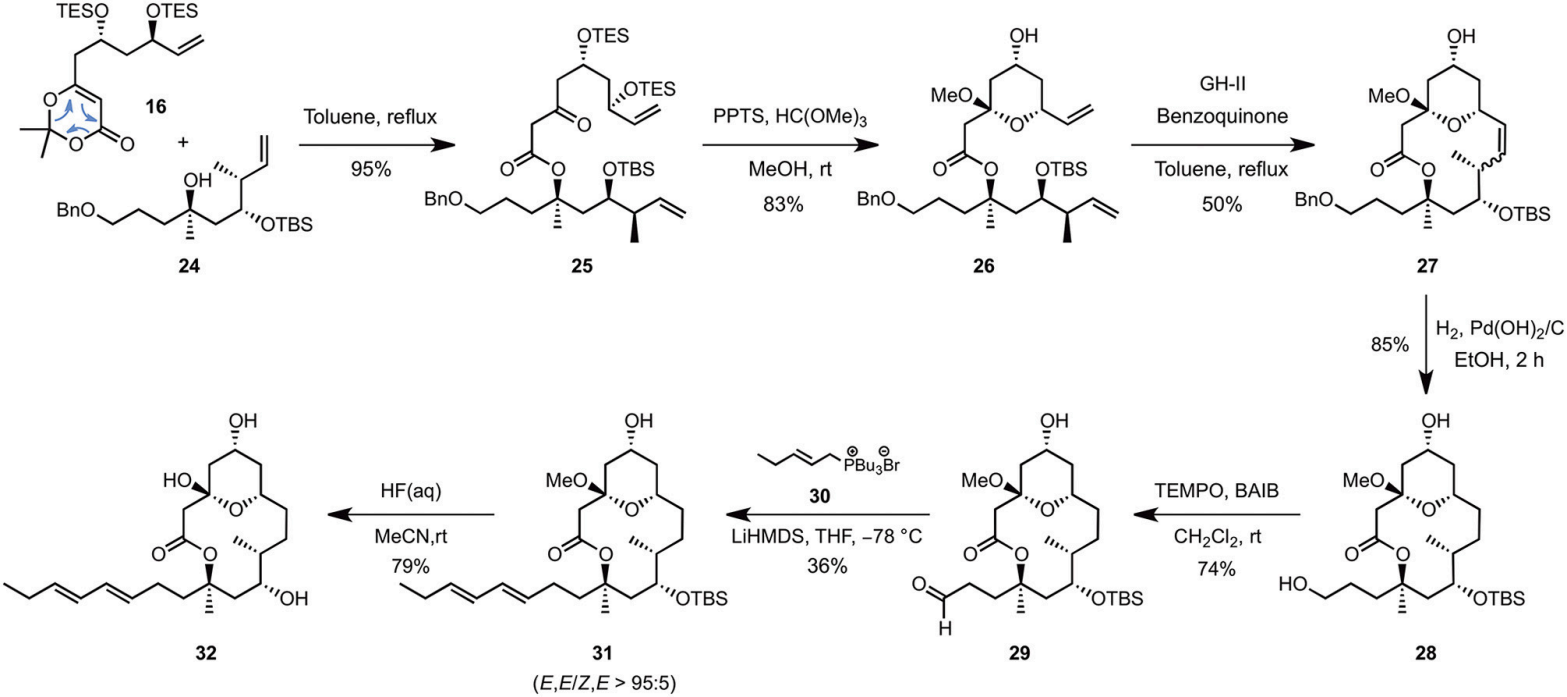
**End game**.

The C1–C8 and C9–C16 fragments were eventually coupled together using the approved intermolecular acyl ketene trapping by mixing the two fragments in refluxing toluene, giving rise to the fully functionalized carbon backbone of the natural product in an excellent yield of 95% (Scheme 4). Hemi-acetal formation (PPTS, MeOH, trimethyl orthoformate), ring-closing metathesis using the Grubbs-Hoveyda 2nd generation catalyst (GH-II) and a final catalytic hydrogenation allowed to isolate the 14-membered macrolactone **27** possessing a hydroxypropyl side-chain appropriate for the elongative olefination (3 steps, 37% overall yield). The latter could be achieved by a selective TEMPO-mediated oxidation (BAIB, CH_2_Cl_2_, rt) followed by a Wittig reaction of the resulting aldehyde **29** with tributyl phosphonium bromide **30** (LiHMDS, THF, −78°C), which enabled the *E,E*-dienyl moiety to be installed in a highly diastereoselective fashion but with a yet unoptimized yield of 27%. Finally, removal of the remaining TBS-protecting group (HF, MeCN, rt) afforded the putative structure of 11-*epi*-lyngbouilloside aglycone **32** as a single diastereoisomer in 20 steps and 2% overall yield starting from commercially available 3-methyl-but-3-enol (**11**). Unfortunately, comparison of the NMR chemical shifts of our synthetic aglycon with the ones reported for natural lyngbouilloside, particularly in the C9-C13 region, revealed some disparities suggesting one or more of the stereochemical configurations of the natural product needed to be reassigned.

## Conclusion

In summary, we have completed the synthesis of what we believed was the actual structure of lyngbouilloside aglycon. Unfortunately, after careful analysis of the spectroscopic data of our final product with the ones reported for lyngbouilloside, some discrepancies still remained. This observation combined with the recent syntheses of lyngbyaloside B and C by Fuwa (Fuwa et al., 2016) and Taylor (Chang et al., 2015), suggest not only a stereochemical reassignment for C11, but also for C10 and C13. Nonetheless, our strategy featuring a ring-closing metathesis (RCM) to form the 14-membered ring macrolactone, a late stage side chain introduction *via* a Wittig olefination and a glycosylation to introduce the rhamnose should allow to complete the synthesis of lyngbouilloside and irrevocably confirm its structure.

## Author contributions

SA and JC conceived the project and designed the research. AK, JG, AE, and RL carried out the experimental work. CP and EB were in charge of the preparative HPLC separations. SA and JG wrote the manuscript. All authors commented on the manuscript.

### Conflict of interest statement

The authors declare that the research was conducted in the absence of any commercial or financial relationships that could be construed as a potential conflict of interest. The reviewer ZX and handling Editor declared their shared affiliation, and the handling Editor states that the process nevertheless met the standards of a fair and objective review.
