# Supplementary material for: Total Synthesis of Putative 11-epi-Lyngbouilloside Aglycon
Source: Front Chem. 2016 Aug 9;4:34. doi: 10.3389/fchem.2016.00034 (PMC4977289; doi:10.3389/fchem.2016.00034)
Supplement: Supplementary file 1 [file DataSheet1.PDF]

Total synthesis of putative 11-*epi*-lyngbouilloside aglycon

I. General Experimental

The reactions were run under argon atmosphere in oven-dried glassware unless otherwise specified. Dichloromethane was distilled from calcium hydride. THF and Et_2O were distilled from sodium/benzophenone. DMF was distilled under vacuum over $MgSO_4$, and pyridine was stored over NaOH pellets. Analytical thin layer chromatography (TLC) was performed on silica gel plates (Merck $60F_{254}$) visualized either with a UV lamp (254 nm) or by using solutions of *p*-anisaldehyde/sulfuric acid/acetic acid in EtOH, phosphomolybdic acid in EtOH or $KMnO_4/K_2CO_3$/AcOH in water followed by heating. Flash chromatographies were performed on silica gel (60-230 mesh mesh). All the reactions were carried out under N_2 atmosphere. Organic extracts were dried over anhydrous Na_2SO_4. Infrared spectra (IR) were recorded on a Bruker TENSOR™ 27 (IRTF) and wave-numbers are indicated in cm^{-1}. ^{1}H NMR spectra were recorded on a Bruker AVANCE 400 at 400 MHz in $CDCl_3$ and data are reported as follows: chemical shift in parts per million from tetramethylsilane as an internal standard, multiplicity (s = singlet, d = doublet, t = triplet, q = quartet, m = multiplet or overlap of nonequivalent resonances), integration. ^{13}C NMR spectra were recorded at 100 MHz in $CDCl_3$ (unless otherwise specified) and data were reported as follows: chemical shift in parts per million from tetramethylsilane with the solvent as an internal indicator ($CDCl_3$ δ 77.0 ppm), multiplicity with respect to proton (deduced from DEPT experiments, s = quaternary C, d = CH, t = CH_2, q = CH_3). Mass spectra (MS) were recorded using a Hewlett-Packard tandem 5890A/5971 GCMS (70 eV). High resolution mass spectra were performed by "Groupe de Spectrométrie de masse de l'Université Pierre et Marie Curie (Paris)".

II. Experimental and Spectral data

6-[(2*R*)-2-Hydroxyhex-5-en-1-yl]-2,2-dimethyl-2,4-dihydro-1,3-dioxin-4-one (12)

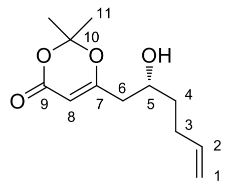

MW (g/mol): 226.2689 **Molecular formula:** $C_{12}H_{18}O_4$

To a mixture of 4-pentenal (1.45 g, 17.2 mmol) and $TiCl_4$ (2.2 mL, 20.6 mmol) in anhydrous DCM (124 mL) at −78 °C, was added the enol silane (4.4 g, 20.6 mmol) and the resulting orange solution was stirred for 2 h. The cold bath was then removed and stirring was continued for 2 h. The reaction mixture was diluted with DCM (30 mL) and a saturated aqueous solution of $NaHCO_3$ was added drop-wise until gas evolution ceased. The mixture was filtered through a pad of Celite© and organic layer was separated (2 x 50 mL). The combined organic layers were dried over anhydrous Na_2SO_4, filtered and concentrated under reduced pressure. The crude residue was purified by flash column chromatography over silica gel (PE/EtOAc = 25:75) to afford the desired product as a colourless oil (3.27 g, 84 %). The two enantiomers were separated by preparative Supercritical Fluid Chromatography (SFC): CHIRALPAK AD-H, 150 bar, 3 $mL.min^{-1}$, $MeOH/CO_2$ = 1:9 (30 injections) and the desired enantiomer **12** was obtained with an *ee* > 99%. Spectroscopic and physical data matched with the ones reported in the literature.[1]

$[\alpha]_D^{20}$ −16.3 (*c* 0.34, $CHCl_3$)

^{1}H-NMR (400 MHz, $CDCl_3$) δ (ppm): 5.76 (m, 1H, H_2), 5.25 (s, 1H, H_8), 5.03-4.93 (m, 2H, H_1), 3.87 (m, 1H, H_5), 2.37-2.25 (m, 2H, H_3), 2.20-2.07 (m, 2H, H_6, H_{12}), 1.63 (s, 6H, H_{11}), 1.57-1.52 (m, 3H, H_4, H_6).

^{13}C-NMR (100 MHz, $CDCl_3$) δ (ppm): 169.1 (s, C_7), 161.0 (s, C_9), 137.8 (d, C2), 115.5 (t, C_1), 106.6 (s, C_{10}), 95.1 (d, C_8), 68.4 (d, C_5), 41.7 (t, C_6), 36.3 (t, C_4), 29.8 (t, C_3), 25.3 (q, C_{10}), 24.9 (q, C_{10}).

[1] A. ElMarrouni, R. Lebeuf, J. Gebauer, M. Heras, S. Arseniyadis, J. Cossy, *Org. lett.* **2012**, *14*, 314.

6-[(2*S*)-2-Hydroxy-4-oxohex-5-en-1-yl]-2,2-dimethyl-2,4-dihydro-1,3-dioxin-4-one (14).

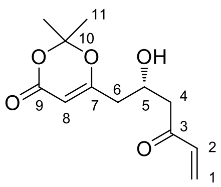

MW (g/mol): 240.2524 **Molecular formula:** $C_{12}H_{16}O_5$

To a solution of (**12**) (1.1 g, 5.1 mmol) in CH_2Cl_2 (17 mL) at rt was added SeO_2 (1.14 g, 10.2 mmol) followed by *t*-BuOOH (6.5 ml of a 5.5 M solution in decane, 35.7 mmol) and the mixture was stirred for 24 h at reflux. After addition of H_2O (10 mL) and vigorous stirring for 30 min, the aqueous phase was extracted with CH_2Cl_2 (3 x 20 mL) and the combined organic phases were dried over anhydrous Na_2SO_4. Evaporation of the solvent left a residue, which was dissolved in CH_2Cl_2 and filtered through a short pad of silica. Elution with Et_2O and evaporation of the solvent under reduced pressure gave 700 mg of the crude diol, which were dissolved in CH_2Cl_2 (55 ml) and treated with MnO_2 (1.2 g, 14 mmol) for 2 h at reflux. A second portion of MnO_2 (1.2 g, 14 mmol) was then added and the resulting reaction mixture was stirred for 2 additional hours at the same temperature. The reaction mixture was then diluted with CH_2Cl_2 (30 mL) and filtered through Celite©. Evaporation of the solvent and purification of the crude residue by flash column chromatography over silica gel (CH_2Cl_2/Acetone: 95/5) afforded enone **14** (56%) as a yellowish viscous oil. Spectroscopic and physical data matched with the ones reported in the literature.[1]

^{1}H-NMR (400 MHz, $CDCl_3$) δ (ppm): 6.30 (dd, J = 17.8, 10.3 Hz, 1H, H_2), 6.19 (d, J = 17.8 Hz, 1H, H_1), 5.88 (d, J = 10.3 Hz, 1H, H_2), 5.29 (s, 1H, H_8), 4.34 (m, 1H, H_5), 2.80-2.67 (m, 2H, H_4), 2.44-2.29 (m, 2H, H_6), 1.64 (s, 3H, H_{11}), 1.63 (s, 3H, H_{11}), 1.50 (br s, 1H, H_{12}).
^{13}C-NMR (100 MHz, $CDCl_3$) δ (ppm): 200.3 (s, C_3), 168.3 (s, C_7), 161.0 (s, C_9), 136.4 (d, C_2), 129.8 (t, C_1), 106.7 (s, C_{10}), 95.5 (d, C_8), 64.7 (d, C_5), 45.1 (t, C_4), 40.5 (t, C_6), 25.5 (q, C_{11}), 24.6 (q, C_{11}).

6-((2*R*,4*R*)-2,4-Dihydroxyhex-5-en-1-yl)-2,2-dimethyl-4H-1,3-dioxin-4-one (15)

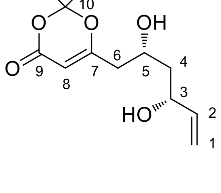

MW (g/mol): 242.27 **Molecular formula:** $C_{12}H_{18}O_5$

To a solution of hydroxyketone **14** (0.88 g, 3.66 mmol) in a 1:1 MeCN/AcOH mixture (62 mL) at −30 °C was added tetramethylammonium triacetoxyborohydride (7.7 g, 29.3 mmol). After stirring overnight at the same temperature, the reaction mixture was poured into a saturated aq. solution of $NaHCO_3$ (400 mL). The solution was stirred again for 20 min. The two layers were separated and the aqueous fraction was extracted with EtOAc (2 x 100 mL). The combined organic fractions were washed with brine, dried over Na_2SO_4, filtered, and concentrated under reduced pressure. The obtained residue was purified by flash column chromatography over silica gel (Et_2O, 100%) to afford 6-((2*R*,4*R*)-2,4-dihydroxyhex-5-en-1-yl)-2,2-dimethyl-4H-1,3-dioxin-4-one (**15**) as a white solid (659 mg, 74%).

$[\alpha]_D^{20}$ −15.5 (*c* 0.76, $CHCl_3$).
IR (ATR) *ν* (cm^{-1}): 3410, 2923, 1711, 1633, 1392, 1378, 1276, 1204, 1015, 807, 688.
^{1}H-NMR (400 MHz, $CDCl_3$) *δ* (ppm): 5.92 (ddd, *J* = 17.1, 10.5, 5.6 Hz, 1H, H_2), 5.33 (s, 1H, H_8), 5,30 (d, *J* = 16.0 Hz, 1H, H1), 5.17 (d, *J* = 10.5 Hz, 1H), 4.49 (s, 1H, H_3), 4.23 (m, 1H, H_5), 2.41 (m, 2H, H_6), 1.78 (m, 1H, H_4), 1.69 (m, 6H, H_{11}), 1.68 (m, 1H, H_4).
^{13}C-NMR (100 MHz, $CDCl_3$) *δ* (ppm): 169.1 (s, C_7), 161.4 (s, C_9), 140.2 (d, C_2), 115.2 (t, C_1), 106.8 (s, C_{10}), 95.2 (d, C_8), 70.5 (d, C_3), 66.1 (d, C_5), 42.2 (t, C_4), 41.8 (t, C_6), 25.4 (q, C_{11}), 25.0 (q, C_{11}).
HRMS (ESI) *m/z*: calculated for $C_{12}H_{18}O_5$ $[M+Na]^+$: 265.10464, found: 265.10501.

6-((2*R*,4*R*)-2,4-Dihydroxyhex-5-en-1-yl)-2,2-dimethyl-4H-1,3-dioxin-4-one (16)

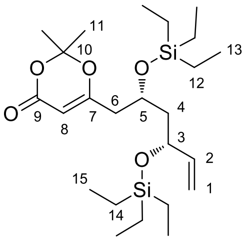

MW (g/mol): 470.79 **Molecular formula:** $C_{24}H_{46}O_5Si_2$

To a solution of the diol (650 mg, 2.69 mmol) in CH_2Cl_2 (65 mL) at 0 °C were added imidazole (731 mg, 10.7 mmol) and TESCl (1.36 mL, 8.0 mmol). After stirring 1.5 h at the same temperature, the reaction mixture was poured into a saturated aq. solution of $NaHCO_3$ (30 mL). The two layers were separated and the aqueous fraction was extracted twice with CH_2Cl_2 (2 x 30 mL). The combined organic layers were then washed with brine (20 mL), dried over Na_2SO_4, filtered and concentrated under reduced pressure. The crude residue was finally purified by flash column

chromatography over silica gel (PE/Et_2O = 90:10) to afford 6-((2*R*,4*R*)-2,4-dihydroxyhex-5-en-1-yl)-2,2-dimethyl-4H-1,3-dioxin-4-one (**16**) as colourless oil (1.2 g, 95%).

$[\alpha]_D^{20}$ −2.3 (*c* 0.43, $CHCl_3$).
IR (ATR) ν (cm^{-1}): 2955, 2912, 2877, 1736, 1637, 1390, 1376, 1271, 1204, 1093, 1014, 742.
^{1}H-NMR (400 MHz, $CDCl_3$) δ (ppm): 5.78 (ddd, J = 17.3, 10.2, 7.3 Hz, 1H, H_2), 5.27 (s, 1H, H_8), 5.14 (d, J = 17.2 Hz, 1H, H_1), 5.06 (d, J = 10.3 Hz, 1H, H_1), 4.19 (m, 1H, H_3), 4.10 (p_{app}, J = 6.0 Hz, 1H, H_5), 2.40 (m, 2H, H_6), 1.78-1.58 (m, 2H, H_4),), 1.68 (s, 3H, H_{11}), 1.67 (s, 3H, H_{11}), 1.00-0.89 (m, 18H, H_{13}+H_{15}), 0.64-0.53 (m, 12H, H_{12}+H_{14}).
^{13}C-NMR (100 MHz, $CDCl_3$) δ (ppm): 169.3 (s, C_7), 161.3 (s, C_9), 141.7 (d, C_2), 114.9 (t, C_1), 106.5 (s, C_{10}), 95.4 (d, C_8), 71.8 (d, C_3), 67.1 (d, C_5), 46.5 (t, C_4), 42.8 (t, C_6), 25.8 (q, C_{11}), 24.7 (q, C_{11}), 7.0 (q, C_{13}+C_{15}), 5.3 (t, C_{12}+C_{14}).
HRMS (ESI) *m/z*: calculated for $C_{24}H_{46}O_5Si_2$ $[M+Na]^+$: 493.27760, found: 493.27781.

1-Methoxy-4-[(3-methylbut-3-en-1-yl)oxy]benzene (17)[2]

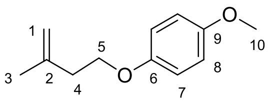

MW (g/mol): 192.25 **Molecular formula:** $C_{12}H_{16}O_2$

To a solution of 3-methylbut-3-en-1-ol (**11**) (12 mL, 116 mmol), 4-methoxyphenol (21.6 g, 174 mmol) and triphenylphosphine (39.5 g, 150 mmol) in anhydrous THF (400 mL) at 0 °C was added DIAD (29.3 mL, 150 mmol) drop-wise, and the resulting mixture was stirred at reflux for 3 h. The solvent was then evaporated under reduced pressure and the salts were precipitated by addition of Et_2O (200 mL) and filtered through a plug of Celite©. The solvent was evaporated under reduced pressure and the crude residue was purified by flash column chromatography over silica gel (Et_2O/PE = 10:90) to afford 1-methoxy-4-[(3-methylbut-3-en-1-yl)oxy]benzene **17** (15.9 g, 71%), which spectroscopic and physical data matched the ones reported in the literature.[2]

^{1}H-NMR (400 MHz, $CHCl_3$) δ (ppm): 6.87 (m, 4H, H_7+H_8), 4.87 (s, lH, H_1), 4.83 (s, lH, H_1), 4.05 (t, J = 6.8 Hz, 2H H_4), 3.78 (s, 3H, H_{10}), 2.51 (t, J = 6.8 Hz, 2H, H_5), 1.83 (s, 3H, H_3).
^{13}C-NMR (400 MHz, $CHCl_3$) δ (ppm):153.8 (s, C_9), 153.1 (s, C_6), 142.3 (s, C_2), 115.6 (d, C_7), 114.6 (d, C_8), 111.9 (t, C_1), 67.1 (t, C_5), 55.6 (q, C_{10}), 37.3 (t, C_4), 22.8 (q, C_3).

[2] Corey, E. J.; Guzman-Perez, A.; Noe, M. *J. Am. Chem. Soc.*, **1995**, *117*, 10805-10816.

(2*S*)-4-(4-Methoxyphenoxy)-2-methylbutane-1,2-diol (18)[2]

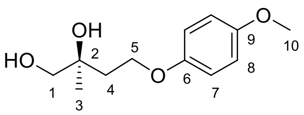

MW (g/mol): 226.27 **Molecular formula:** $C_{12}H_{18}O_4$

To a solution of olefin **17** (7.95 g, 41.3 mmol) in *t*-BuOH (205 mL) at 0 °C was added a solution of AD-mix-α (57.8 g, 1.4 g/mmol) and $(DHQ)_2$PHAL (150 mg, 0.19 mmol) in water (205 mL). After stirring for 24 h at the same temperature, $Na_2S_2O_3$ (2.0 g) was added and the mixture was stirred for an additional 20 min. The crude residue was then extracted three times with an EtOAc/hexanes (2:1) mixture (3 x 100 mL) and the combined organic phases were washed with brine (100 mL). The organic layer was finally dried over anhydrous Na_2SO_4, filtered, and evaporatedunder reduced pressure, and the residue was purified by flash column chromatography over silica gel (EtOAc, 100%) to afford (*S*)-4-(4-methoxyphenoxy)-2-methylbutane-1,2-diol **18** (9.30 g, 100%) as a white solid, which spectroscopic and physical data matched the ones reported in the literature.[2]

^{1}H-NMR (400 MHz, $CDC1_3$) δ 6.82 (m, 4H, H_7+H_8), 4.13 (ddd, J = 9.7, 7.5, 4.9 Hz, lH, H_5), 4.06 (ddd, J = 9.6, 6.4, 5.3 Hz, lH, H_5), 3.74 (s, 3H, H_{10}), 3.51 (dd, J = 11.1, 4.9 Hz, lH, H_1), 3.45 (dd, J = 11.1, 5.3 Hz, lH, H_1), 3.19 (brs, lH, OH), 3.05 (m. lH, OH), 2.06 (ddd, J = 14.7, 7.5, 5.2 Hz, lH, H_4), 1.89 (ddd, J = 14.7, 6.4, 5.1 Hz, lH, H_4), 1.23 (s, 3H, H_3).
^{13}C-NMR (100 MHz, $CDCl_3$) δ 154.0 (s, C_9), 152.4 s, C_6), 115.5 (d, C_7), 114.6 (d, C_8), 72.4 (t, C_1), 69.9 (t, C_2), 65.2(t, C_5), 55.6 (q, C_{10}), 37.4 (t, C_4), 23.9 (q, C_3).

(*S*)-2-[2-(4-Methoxyphenoxy)ethyl]-2-methyloxirane (19)

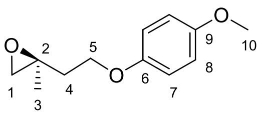

MW (g/mol): 208.25 **Molecular formula:** $C_{12}H_{16}O_3$

To a solution of diol **18** (18.7 g, 82.6 mmol) and triethylamine (14.2 mL, 101.8 mmol) in CH_2Cl_2 (150 mL) at 0 °C was added MsCl (7.0 mL, 90.0 mmol) drop-wise. After stirring for 45 min at 0 °C, most of the CH_2Cl_2 was evaporated under reduced pressure (T_{bath} < 20 °C) and Et_2O (200 mL) was added to allow the salts to precipitate. The salts were filtered off and the solution was washed with water (50 mL) and brine (50 mL). The combined organic layers were then dried over Na_2SO_4, filtered and evaporated, and the crude residue was dissolved in MeOH (310 mL). The reaction

mixture was allowed to cool at 0 °C before K_2CO_3 (35.0 g, 253.2 mmol) was added in one portion, and stirring was continued for 30 min at rt. Most of the MeOH was then evaporated (T_{bath} < 20 °C) and the residue was extracted with Et_2O (300 mL). The organic phase was finally washed with water (3 x 100 mL) and brine (100 mL), and the organic layer was dried over Na_2SO_4, filtered and evaporated. The obtained residue was purified by flash chromatography (EtOAc/PE = 20:80) to afford (*S*)-2-[2-(4-methoxyphenoxy)ethyl]-2-methyloxirane **19** (15.13 g, 88%) as a white solid. Spectroscopic and physical data matched with the ones reported in the literature.[1]

^{1}H-NMR (400 MHz, $CDCl_3$) δ 6.83 (d, J = 0.6 Hz, 4H, H_7+H_8), 4.06-3.93 (m, 2H, H_5), 3.77 (s, 3H, H_{10}), 2.74 (d, J = 4.8 Hz, 1H, H_1), 2.63 (d, J = 4.8 Hz, 1H, H_1), 2.15-1.95 (m, 2H, H_4), 1.40 (s, 3H, H_3)

^{13}C-NMR (100 MHz, $CDCl_3$) δ 154.0 (s, C_6 or C_9), 152.9 (s, C_6 or C_9), 115.5 (d, C_7), 114.8 (d, C_8), 65.0 (t, C_5), 55.86 (q, C_{10}), 55.4 (s, C_2), 54.1 (t, C_1), 36.2 (t, C_4), 21.7 (q, C_3).

(*R*)-Triisopropyl((1-(4-methoxyphenoxy)-3-methylhex-5-en-3-yl)oxy)silane (20)

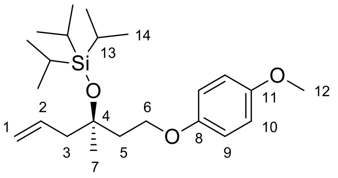

MW (g/mol): 392.65

Molecular formula: $C_{23}H_{40}O_3Si$

To a solution of Li_2CuCl_4 (0.1 M in THF) (0.1 mmol, 1 mL) at −40 °C was added a solution of vinylmagnesium bromide (1 M in THF) (2 mL, 2.0 mmol). The dark reaction mixture was stirred for 1 h at the same temperature and then epoxyde **19** (208 mg, 1.0 mmol) diluted in THF (2.6 mL) was added. After stirring 1 h at −40 °C, 1 h at −20 °C and overnight at 0 °C, a saturated aq. solution of NH_4Cl (20 mL) was poured into the reaction mixture. The two layers were separated and the aqueous fraction was extracted twice with EtOAc (2 x 20 mL). The combined organic layers were then washed with brine (10 mL), dried over Na_2SO_4, filtered and concentrated under reduced pressure. The crude residue was finally purified by flash column chromatography over silica gel (PE/Et_2O = 80:20) to afford the tertiary free alcohol as colourless oil (218 mg, 92%).

$[\alpha]_D^{20}$ +2.0 (*c* 1.3, $CHCl_3$).

IR (ATR) ν (cm^{-1}): 3446, 2832, 1507, 1228, 1037, 916, 824, 736.

^{1}H-NMR (400 MHz, $CDCl_3$) δ 6.90-6.78 (m, 4H, H_9+H_{10}), 5.91 (ddt, J = 16.9, 10.4, 7.4 Hz, 1H, H_2), 5.16 (m, 2H, H_1), 4.15 (t, J = 6.3 Hz, 2H, H_6), 3.78 (s, 3H, H_{12}), 2.43 (s br, 1H, OH), 2.33 (d, J = 6.8 Hz, 2H, H_3), 1.97 (m, 2H, H_5), 1.27 (s, 3H, H_7).
^{13}C-NMR (100 MHz, $CDCl_3$) δ 154.1 (s, C_8 or C_{11}), 152.8 (s, C_8 or C_{11}), 134.1 (d, C_2), 118.8 (t, C_1), 115.6 (d, C_9 or C_{10}), 114.8 (d, C_9 or C_{10}), 71.9 (s, C_4), 65.7 (t, C_6), 55.8 (q, C_{12}), 47.1 (t, C_3), 39.9 (t, C_5), 27.0 (q, C_7).
HRMS (ESI) *m/z*: calculated for $C_{14}H_{20}O_3$ $[M+Na]^+$: 259.13047, found: 259.13057.

To a solution of the tertiary free alcohol (1.9 g, 8.0 mmol) in CH_2Cl_2 (8 mL) at 0 °C were added 2,6-lutidine (1.9 mL, 16.1 mmol) and TIPSOTf (3.2 mL, 12.1 mmol) drop-wise. The resulting mixture was stirred for 1 h at rt before a saturated aq. solution of $NaHCO_3$ (20 mL) was added. The two layers were separated and the aqueous fraction was extracted twice with CH_2Cl_2 (2 x 20 mL). The combined organic layers were washed with brine (10 mL), dried over Na_2SO_4, filtered, and concentrated under reduced pressure. The crude residue was finally purified by flash column chromatography over silica gel (PE/Et_2O = 98:2) to afford (*R*)-triisopropyl((1-(4-methoxyphenoxy)-3-methylhex-5-en-3-yl)oxy)silane (**20**) as colourless oil (2.98 g, 95%).

(*R*)-((6-(Benzyloxy)-1-(4-methoxyphenoxy)-3-methylhexan-3-yl)oxy)triisopropylsilane (21)

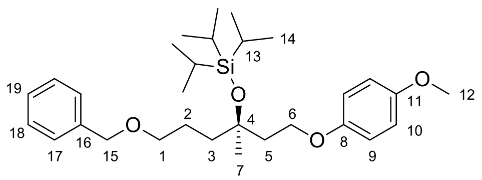

MW (g/mol): 500.78 **Molecular formula:** $C_{30}H_{48}O_4Si$

To a solution of (*R*)-triisopropyl((1-(4-methoxyphenoxy)-3-methylhex-5-en-3-yl)oxy)silane **20** (2.9 g, 7.4 mmol) in THF (33 mL) at 0 °C was added $BH_3.Me_2S$ (2.7 mL, 28.1 mmol) drop-wise. The resulting mixture was stirred for 4 h at rt before a 6 M aq. solution of NaOH (22 mL) followed by a 35 % wt aq. solution of H_2O_2 (20 mL) were added. The resulting mixture was stirred for three additional hours at rt and then, the aqueous fraction was extracted twice with Et_2O (2 x 20 mL). The combined organic layers were washed with a saturated aqueous solution of $Na_2S_2O_3$ (10 mL), dried over Na_2SO_4, filtered, and concentrated under reduced pressure. The crude residue was finally filtrated through a pad of silica (PE/Et_2O = 90:10) and used in the next step without further purification.

To a solution of the primary alcohol (2.5 g, 6.1 mmol) in a (1:1) THF/DMF mixture (15 mL) at 0 °C was added sodium hydride (316 mg, 7.91 mmol). The resulting mixture was stirred for 1 h at the same temperature before benzylbromide (950 µL, 7.91 mmol) was added dropwise. After stirring overnigth at rt, the mixture was diluted with EtOAc (20mL) and poured into a saturated aq. solution of $NaHCO_3$ (50 mL). The two layers were separated and the aqueous fraction was extracted twice with EtOAc (2 x 50 mL). The combined organic layers were washed with brine (10 mL), dried over Na_2SO_4, filtered, and concentrated under reduced pressure. The crude residue was finally purified by flash column chromatography (PE/Et_2O, 98/2) to afford (*R*)-((6-(benzyloxy)-1-(4-methoxyphenoxy)-3-methylhexan-3-yl)oxy)triisopropylsilane (**21**) as colourless oil (2.7 g, 74% over two steps).

$[\alpha]_D^{20}$ +2.0 (*c* 1.15, $CHCl_3$).

IR (ATR) *ν* (cm^{-1}): 2943, 2864, 1508, 1463, 1229, 1040, 882, 823, 734, 676.

^{1}H-NMR (400 MHz, $CDCl_3$) *δ* 7.37-7.26 (m, 5H, H_{17}+H_{18}+H_{19}), 6.81 (s, 4H, H_9+H_{10}), 4.51 (s, 2H, H_{15}), 4.04 (t, *J* = 7.1 Hz, 2H, H_6), 3.77 (s, 3H, H_{12}), 3.48 (m, 2H, H_1), 2.00 (t, *J* = 7.1 Hz, 2H, H_5), 1.77-1.52 (m, 4H, H_2+H_3), 1.31 (s, 3H, H_7), 1.06 (s, 21H, H_{13}+H_{14}).

^{13}C-NMR (100 MHz, $CDCl_3$) *δ* 153.8 (s, C_8 or C_{11}), 153.3 (s, C_8 or C_{11}), 138.8 (s, C_{16}), 128.5 (d, C_{17} or C_{18} or C_{19}), 127.7 (d, C_{17} or C_{18} or C_{19}), 127.6 (d, C_{17} or C_{18} or C_{19}), 115.4 (d, C_9 or C_{10}), 114.7 (d, C_9 or C_{10}), 74.7 (t, C_{15}), 72.9 (t, C_1), 70.9 (s, C_4), 65.3 (t, C_6), 55.9 (q, C_{12}), 41.0 (t, C_5), 39.6 (t, C_2 or C_3), 28.5 (q, C_7), 25.3 (t, C_2 or C_3), 18.6 (q, C_{14}), 13.6 (d, C_{13}).

HRMS (ESI) *m/z*: calculated for $C_{30}H_{48}O_4Si$ $[M+Na]^+$: 523.32141, found: 523.32141.

(*R*)-6-(Benzyloxy)-3-methyl-3-((triisopropylsilyl)oxy)hexan-1-ol

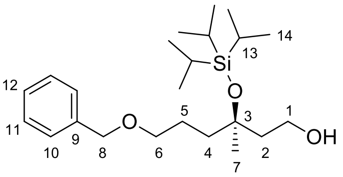

MW (g/mol): 394.66 **Molecular formula:** $C_{23}H_{42}O_3Si$

(*R*)-((6-(benzyloxy)-1-(4-methoxyphenoxy)-3-methylhexan-3-yl)oxy)triisopropylsilane **21** (3.5 g, 6.99 mmol) was dissolved in acetonitrile (108 mL) and the temperature was allowed to cool to 0 °C. A solution of CAN (9.6 g, 17.5 mmol) in water (27 mL) was added and stirring was continued for 10 min at rt. EtOAc (100 mL) was then added followed by water (100 mL) and the organic layer was separated, washed twice with a 1M aq. solution of NaOH (100 mL), dried over Na_2SO_4, filtered and concentrated under reduced pressure. The crude residue was finally purified by flash column

chromatography over silica gel (PE/Et_2O = 60:40) to afford (*R*)-6-(benzyloxy)-3-methyl-3-((triisopropylsilyl)oxy)hexan-1-ol as pale yellow oil (2.45 g, 89%).

$[\alpha]_D^{20}$ −3.7 (*c* 1.49, $CHCl_3$).
IR (ATR) ν (cm^{-1}): 3385, 2942, 2864, 1462, 1375, 1361, 1037, 881, 734, 670.
^{1}H-NMR (400 MHz, $CDCl_3$) δ 7.40-7.26 (m, 5H, H_9+H_{10}+H_{11}), 4.51 (s, 2H, H_8), 3.79 (td, *J* = 6.3, 2.1 Hz, 2H, H_1), 3.47 (m, 2H, H_6), 1.79 (td, *J* = 6.3, 3.4 Hz, 2H, H_2), 1.71 (m, 1H, H_4), 1.66-1.57 (m, 3H, H_4+H_5), 1.29 (s, 3H, H_7), 1.06 (s, 21H, H_{13}+H_{14}).
^{13}C-NMR (100 MHz, $CDCl_3$) δ 138.66 (s, C_9), 128.50 (d, C_{10} or C_{11} or C_{12}), 127.71 (d, C_{10} or C_{11} or C_{12}), 127.67 (d, C_{10} or C_{11} or C_{12}), 76.58 (s, C_3), 73.01 (t, C_8), 70.71 (t, C_6), 59.94 (t, C_1), 43.06 (t, C_2), 39.29 (t, C_4), 28.13 (q, C_7), 25.58 (t, C_5), 18.56 (q, C_{14}), 13.69 (d, C_{13}).
HRMS (ESI) *m/z*: calculated for $C_{23}H_{42}O_3Si$ $[M+Na]^+$: 417.27954, found: 417.27994.

(*R*)-6-(Benzyloxy)-3-methyl-3-((triisopropylsilyl)oxy)hexanal (22)

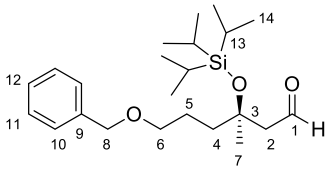

MW (g/mol): 392.75 **Molecular formula:** $C_{23}H_{40}O_3Si$

To a solution of (*R*)-6-(benzyloxy)-3-methyl-3-((triisopropylsilyl)oxy)hexan-1-ol (2.0 g, 5.1 mmol) in CH_2Cl_2 (100 mL) was added Dess-Martin periodinane (2.4 g, 7.6 mmol). The resulting mixture was stirred for 1 h at rt before a saturated aqueous solution of $Na_2S_2O_3$ (50 mL) was added. The two layers were separated and the aqueous fraction was extracted twice with CH_2Cl_2 (2 x 20 mL). The combined organic layers were washed with brine (10 mL), dried over Na_2SO_4, filtered, and concentrated under reduced pressure. The crude residue was finally purified by flash column chromatography (PE/EtOAc = 95:5) to afford (*R*)-6-(benzyloxy)-3-methyl-3-((triisopropylsilyl)oxy)hexanal (**22**) as colourless oil (1.87 g, 94%).

$[\alpha]_D^{20}$ +3.7 (*c* 1.90, $CHCl_3$).
IR (ATR) ν (cm^{-1}): 2943, 2865, 1721, 1462, 1058, 881, 734, 672.
^{1}H-NMR (400 MHz, $CDCl_3$) δ 9.89 (t, *J* = 3.0 Hz, 1H, H_1), 7.46-7.16 (m, 5H, H_{10}+H_{11}+H_{12}), 4.50 (s, 2H, H_8), 3.47 (m, 2H, H_6), 2.54 (dd, *J* = 3.0, 0.8 Hz, 2H, H_2), 1.76-1.60 (m, 4H, H_4+H_5), 1.38 (s, 3H, H_7), 1.07 (s, 21H, H_{13}+H_{14}).

^{13}C-NMR (100 MHz, $CDCl_3$) δ 203.8 (d, C_1), 138.9 (s, C_9), 128.7 (d, C_{10} or C_{11}), 127.94 (d, C_{10} or C_{11}), 127.90 (d, C_{12}), 74.0 (s, C_3), 72.9 (t, C_8), 70.4 (t, C_6), 54.9 (t, C_2), 40.1 (t, C_4 or C_5), 28.3 (q, C_7), 24.8 (t, C_4 or C_5), 18.1 (q, C_{14}), 13.10 (d, C_{13}).

HRMS (ESI) *m/z*: calculated for $C_{23}H_{40}O_3Si$ $[M+Na]^+$: 415.26389, found: 415.26441.

(*Z*)-But-2-en-1-yltrichlorosilane

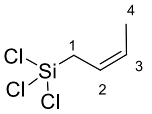

MW (g/mol): 189.54 **Molecular formula:** $C_4H_7Cl_3Si$

To a solution of condensed 1,3-butadiene (5.8 g, 107 mmol) in THF (400 mL) at −78 °C was added $Pd(PPh_3)_4$ (0.2 g, 0.17 mmol) followed by trichlorosilane (6.7 mL, 66.9 mmol). After 15 min, the cooling bath was removed and the mixture was allowed to warm to rt. After 17 h, the reaction flask was fitted with a short-path distillation head and the THF was removed by distillation. The residue was transferred to a distillation-head equipped with a 25 mL round-bottom flask. The THF was removed by distillation and the residue was distilled under reduced pressure (bp ~60 °C at ~20 mm Hg) to give the desired product as a clear and colorless liquid (9.5 g, 75%). ^{1}H NMR spectroscopic analysis of the crude mixture revealed that the *cis* to *trans* ratio was ≥ 99:1. Spectroscopic data is in agreement with data reported in literature.[3]

^{1}H-NMR (400 MHz, $CDCl_3$) δ 5.72 (m, 1H, H_2 or H_3), 5.42 (m, 1H, H_2 or H_3), 2.35 (d, J = 8.2 Hz, 2H, H_1), 1.66 (m, 3H, H_4).

(3*R*,4*R*,6*R*)-9-(Benzyloxy)-3,6-dimethyl-6-((triisopropylsilyl)oxy)non-1-en-4-ol (23)

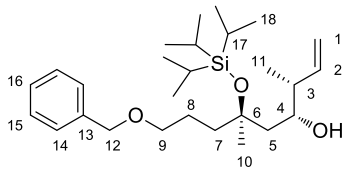

MW (g/mol): 448.75 **Molecular formula:** $C_{27}H_{48}O_3Si$

To a solution of ligand (1.0 g, 3.44 mmol) in CH_2Cl_2 (8 mL) at 0 °C was added freshly distilled DBU (1.5 mL, 10.33 mmol) followed by (*Z*)-but-2-en-1-yltrichlorosilane (712 mg, 3.76 mmol). The ice/water bath was removed and after 1 h the mixture was recooled to 0 °C. The aldehyde (1.23 g,

[3] K. Iseki, Y. Kuroki, M. Takahashi, S. Kishimoto and Y. Kobayashi, *Tetrahedron*, 1997, **53**, 3513

3.13 mmol) diluted in CH_2Cl_2 (5 mL) was then added and the reaction mixture was stirred for an additional hour at the same temperature. The solution was treated with TBAF (2.0 equiv) and after 10 min the solvent was evaporated under reduced pressure. The crude residue was finally purified by flash column chromatography over silica gel (PE/EtOAc = 90:10) to afford (3*R*,4*R*,6*R*)-9-(benzyloxy)-3,6-dimethyl-6-((triisopropylsilyl)oxy)non-1-en-4-ol (**23**) as a 83:17 mixture of diastereoisomers (*syn/syn* : *anti/syn*) (1.35 g, 96%).

$[\alpha]_D^{20}$ +3.6 (*c* 2.0, $CHCl_3$).
IR (ATR) ν (cm^{-1}): 3494, 2943, 2866, 1455, 1100, 996, 913, 882, 734, 675.
Major diastereoisomer: ^{1}H-NMR (400 MHz, $CDCl_3$) δ 7.43-7.26 (m, 5H, H_{14}+H_{15}+H_{16}), 5.78 (m, 1H, H_2), 5.02 (m, 2H, H_1), 4.50 (s, 2H, H_{12}), 3.79 (m, 1H, H_4), 3.58 (s, 1H, OH), 3.46 (m, 2H, H_9), 2.21 (m, 1H, H_3), 1.79-1.44 (m, 6H, H_5+H_7+H_8), 1.33 (s, 3H, H_{10}), 1.12-1.06 (m, 21H, H_{17}+H_{18}), 1.04 (d, J = 6.8 Hz, 3H, H_{11}).
Minor diastereoisomer: ^{1}H-NMR (400 MHz, $CDCl_3$) δ 7.43-7.26 (m, 5H, H_{14}+H_{15}+H_{16}), 5.78 (m, 1H, H_2), 5.02 (m, 2H, H_1), 4.51 (s, 2H, H_{12}), 3.74 (m, 1H, H_4), 3.58 (s, 1H, OH), 3.46 (m, 2H, H_9), 2.21 (m, 1H, H_3), 1.79-1.44 (m, 6H, H_5+H_7+H_8), 1.29 (s, 3H, H_{10}), 1.12-1.06 (m, 21H, H_{17}+H_{18}), 1.04 (d, J = 6.8 Hz, 3H, H_{11}).

The signals of both the major and the minor diastereoisomers could not be assigned separately in the ^{13}C NMR spectrum
^{13}C-NMR (100 MHz, $CDCl_3$) δ 141.4 (d, C_2), 138.6 (s, C_{13}), 128.5 (d, C_{14} or C_{15} or C_{16}), 127.73 (d, C_{14} or C_{15} or C_{16}), 127.69 (d, C_{14} or C_{15} or C_{16}), 114.8 (t, C_1), 77.7 (s, C_6), 73.1 (t, C_{12}), 72.1 (d, C_4), 70.7 (t, C_9), 44.5 (d, C_3), 44.2 (t, C_5), 41.3 (t, C_7), 27.0 (q, C_{10}), 25.7 (t, C_8), 18.6 (q, C_{18}), 15.2 (q, C_{11}), 13.8 (d, C_{17}).
HRMS (ESI) *m/z*: calculated for $C_{27}H_{48}O_3Si$ $[M+Na]^+$: 471.32649, found: 471.32693.

(3*R*,4*R*,6*R*)-9-(Benzyloxy)-3,6-dimethylnon-1-ene-4,6-diol

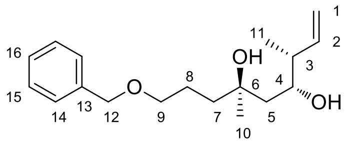

MW (g/mol): 292.41 **Molecular formula:** $C_{18}H_{28}O_3$

To a solution of (3*R*,4*R*,6*R*)-9-(benzyloxy)-3,6-dimethyl-6-((triisopropylsilyl)oxy)non-1-en-4-ol **23** (1.3 g, 2.90 mmol) in THF (13 mL) at 0 °C was added a solution of TBAF (1M in THF) (14.5 mL, 14.48 mmol). After stirring 5 h at room temperature, a saturated aq. solution of $NaHCO_3$ (15 mL)

was poured into the reaction mixture. The two layers were separated and the aqueous fraction was extracted twice with EtOAc (2 x 5 mL). The combined organic layers were then washed with brine (5 mL), dried over Na_2SO_4, filtered and concentrated under reduced pressure. The crude residue was finally purified by flash column chromatography over silica gel (PE/Et_2O = 70:30) to afford (3*R*,4*R*,6*R*)-9-(benzyloxy)-3,6-dimethylnon-1-ene-4,6-diol as a 83:17 mixture of diastereoisomers (*syn/syn*:*anti/syn*) (795 mg, 94%).

$[\alpha]_D^{20}$ +14.4 (*c* 0.59, $CHCl_3$).

IR (ATR) *ν* (cm^{-1}): 3355, 2966, 1717, 1453, 1276, 1112, 914, 713, 698.

Major diastereoisomer: ^{1}H-NMR (400 MHz, $CDCl_3$) *δ* 7.44-7.26 (m, 5H, H_{14}+H_{15}+H_{16}), 5.78 (m, 1H, H_2), 5.07 (m, 2H, H_1), 4.52 (s, 2H, H_{12}), 3.88 (ddd, *J* = 10.7, 5.9, 1.9 Hz, 1H, H_4), 3.50 (td, *J* = 6.1, 2.4 Hz, 2H, H_9), 3.20 (s br, 2H, OH), 2.24 (m, 1H, H_3), 1.73 (m, 2H, H_8), 1.64-1.54 (m, 3H, H_5+H_7), 1.47 (m, 1H, H_5), 1.25 (s, 3H, H_{10}), 1.05 (d, *J* = 6.8 Hz, 3H, H_{11}).

Minor diastereoisomer: ^{1}H-NMR (400 MHz, $CDCl_3$) *δ* 7.44-7.26 (m, 5H, H_{14}+H_{15}+H_{16}), 5.78 (m, 1H, H_2), 5.07 (m, 2H, H_1), 4.52 (s, 2H, H_{12}), 3.82 (m, 1H, H_4), 3.50 (td, *J* = 6.1, 2.4 Hz, 2H, H_9), 3.20 (s br, 2H, OH), 2.24 (m, 1H, H_3), 1.73 (m, 2H, H_8), 1.64-1.54 (m, 3H, H_5+H_7), 1.47 (m, 1H, H_5), 1.20 (s, 3H, H_{10}), 1.04 (d, *J* = 6.8 Hz, 3H, H_{11}).

Major diastereoisomer: ^{13}C-NMR (100 MHz, $CDCl_3$) *δ* 141.0 (d, C_2), 138.1 (s, C_{13}), 128.6 (d, C_{14} or C_{15} or C_{16}), 127.85 (d, C_{14} or C_{15} or C_{16}), 127.84 (d, C_{14} or C_{15} or C_{16}), 115.3 (t, C_1), 73.2 (t, C_{12}), 73.0 (s, C_6), 72.4 (d, C_4), 71.0 (t, C_9), 44.4 (d, C_3), 43.3 (t, C_5), 41.9 (t, C_7), 25.8 (q, C_{10}), 24.1 (t, C_8), 15.2 (q, C_{11}).

Minor diastereoisomer: ^{13}C-NMR (100 MHz, $CDCl_3$) *δ* 140.8 (d, C_2), 138.1 (s, C_{13}), 128.6 (d, C_{14} or C_{15} or C_{16}), 127.85 (d, C_{14} or C_{15} or C_{16}), 127.84 (d, C_{14} or C_{15} or C_{16}), 115.5 (t, C_1), 73.2 (t, C_{12}), 73.0 (s, C_6), 72.1 (d, C_4), 71.0 (t, C_9), 44.4 (d, C_3), 43.8 (t, C_5), 37.5 (t, C_7), 28.9 (q, C_{10}), 25.0 (t, C_8), 15.0 (q, C_{11}).

HRMS (ESI) *m/z*: calculated for $C_{18}H_{28}O_3$ $[M+Na]^+$: 31519307, found: 315.19328.

(4*R*,6*R*,7*R*)-1-(Benzyloxy)-6-((*tert*-butyldimethylsilyl)oxy)-4,7-dimethylnon-8-en-4-ol (24)

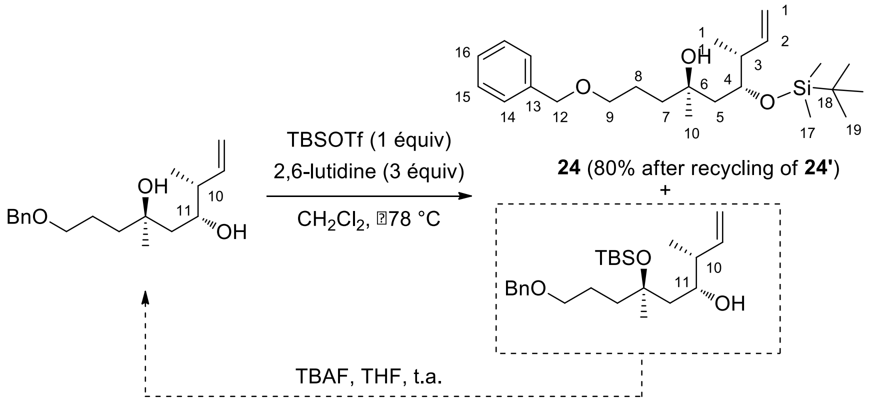

MW (g/mol): 406.67 **Molecular formula:** $C_{24}H_{42}O_3Si$

To a solution of diol (600 mg, 2.05 mmol) in CH_2Cl_2 (20 mL) at −20 °C were added 2,6-lutidine (717 µL, 6.16 mmol) and TBSOTf (518 µL, 2.25 mmol) drop-wise. The resulting mixture was stirred for 30 min at the same temperature before a saturated aq. solution of $NaHCO_3$ (10 mL) was added. The two layers were separated and the aqueous fraction was extracted twice with CH_2Cl_2 (2 x 10 mL). The combined organic layers were washed with brine (10 mL), dried over Na_2SO_4, filtered, and concentrated under reduced pressure. The crude residue was finally purified by flash column chromatography over silica gel (PE/Et_2O = 80:20) to afford the desired compound **24** as a 85:15 mixture of diastereoisomers (*syn/syn*:*anti/syn*) (56% but 80% after recycling of **24'**) and the undesired compound **24'** (39%) which can be converted to **24** after déprotection/reprotection.

$[\alpha]_D^{20}$ +55.2 (*c* 0.66, $CHCl_3$).

IR (ATR) ν (cm^{-1}): 3518, 2955, 2930, 2857, 1455, 1361, 1254, 1099, 1050, 911, 834, 774, 696.

Major diastereoisomer: ^{1}H-NMR (400 MHz, $CDCl_3$) δ 7.40-7.27 (m, 5H, H_{14}+H_{15}+H_{16}), 6.04 (ddd, J = 17.5, 10.8, 5.0 Hz, 1H, H_2), 5.12 (m, 1H, H_1), 5.02 (dt, J = 17.5, 1.9 Hz, 1H, H_1), 4.50 (s, 3H, H_{12}), 4.09 (m, 1H, H_4), 3.84 (s br, 1H,OH), 3.48 (t, J = 6.6 Hz, 2H, H_9), 2.53 (m, 1H, H_3), 1.71 (m, 2H, H_8), 1.60 (m, 1H, H_5), 1.49 (m, 2H, H_7), 1.35 (dd, J = 14.6, 2.5 Hz, 1H, H_5), 1.21 (s, 3H, H_{10}), 0.94 (d, J = 7.0 Hz, 3H, H_{11}), 0.93-0.90 (m, 9H, H_{19}), 0.17 (m, 3H, H_{17}), 0.16 (m, 3H, H_{17}).

Minor diastereoisomer: ^{1}H-NMR (400 MHz, $CDCl_3$) δ 7.40-7.27 (m, 5H, H_{14}+H_{15}+H_{16}), 6.04 (ddd, J = 17.5, 10.8, 5.0 Hz, 1H, H_2), 5.12 (m, 1H, H_1), 5.02 (dt, J = 17.5, 1.9 Hz, 1H, H_1), 4.51 (s, 3H, H_{12}), 4.09 (m, 1H, H_4), 3.84 (s br, 1H,OH), 3.48 (t, J = 6.6 Hz, 2H, H_9), 2.53 (m, 1H, H_3), 1.71 (m, 2H, H_8), 1.60 (m, 1H, H_5), 1.49 (m, 2H, H_7), 1.35 (dd, J = 14.6, 2.5 Hz, 1H, H_5), 1.13 (s, 3H, H_{10}), 0.94 (d, J = 7.0 Hz, 3H, H_{11}), 0.93-0.90 (m, 9H, H_{19}), 0.17 (m, 3H, H_{17}), 0.16 (m, 3H, H_{17}).

Major diastereoisomer: ^{13}C-NMR (100 MHz, $CDCl_3$) δ 138.7 (s, C_{13}), 138.4 (d, C_2), 128.5 (d, C_{14} or C_{15} or C_{16}), 127.8 (d, C_{14} or C_{15} or C_{16}), 128.6 (d, C_{14} or C_{15} or C_{16}), 115.1 (t, C_1), 75.2 (d, C_4),

73.0 (t, C_{12}), 71.8 (s, C_6), 71.2 (t, C_9), 42.3 (d, C_3), 40.9 (t, C_7), 39.8 (t, C_5), 26.1 (q, C_{10} or C_{19}), 26.0 (q, C_{10} or C_{19}), 24.2 (t, C_8), 18.1 (s, C_{18}), 14.9 (q, C_{11}), −3.1 (q, C_{17}), −4.5 (q, C_{17}).

Minor diastereoisomer: ^{13}C-NMR (100 MHz, $CDCl_3$) δ 138.7 (s, C_{13}), 138.4 (d, C_2), 128.5 (d, C_{14} or C_{15} or C_{16}), 127.8 (d, C_{14} or C_{15} or C_{16}), 128.6 (d, C_{14} or C_{15} or C_{16}), 115.1 (t, C_1), 74.9 (d, C_4), 73.0 (t, C_{12}), 72.0 (s, C_6), 71.0 (t, C_9), 42.3 (d, C_3), 40.3 (t, C_7), 37.5 (t, C_5), 28.1 (q, C_{10} or C_{19}), 26.0 (q, C_{10} or C_{19}), 25.0 (t, C_8), 18.1 (s, C_{18}), 14.9 (q, C_{11}), −3.1 (q, C_{17}), −4.5 (q, C_{17}).

HRMS (ESI) *m/z*: calculated for $C_{24}H_{42}O_3Si$ $[M+Na]^+$: 429.27954, found: 429.27983.

(5*R*,7*R*)-(4*R*,6*R*,7*R*)-1-(Benzyloxy)-6-((*tert*-butyldimethylsilyl)oxy)-4,7-dimethylnon-8-en-4-yl 3-oxo-5,7-bis((triethylsilyl)oxy)non-8-enoate (25)

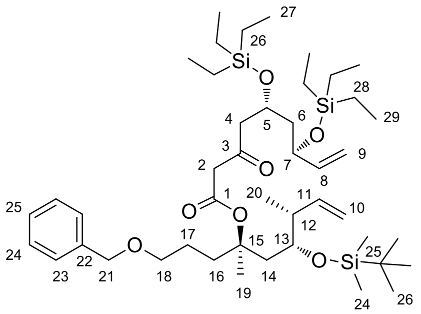

MW (g/mol): 819.38 **Molecular formula:** $C_{45}H_{82}O_7Si_3$

A solution of dioxinone **16** (1.83 g, 3.89 mmol) and alcohol **24** (1.1 g, 2.70 mmol) in dry toluene (9.6 mL) was refluxed for 2 h until complete conversion of the starting material (reaction monitored by TLC). The solvent was then removed under reduced pressure and the crude residue was purified by flash column chromatography over silica gel (PE/Et_2O, 98/2) to afford (5*R*,7*R*)-(4*R*,6*R*,7*R*)-1-(benzyloxy)-6-((*tert*-butyldimethylsilyl)oxy)-4,7-dimethylnon-8-en-4-yl-3-oxo-5,7-bis((triethylsilyl)oxy)non-8-enoate (**25**) as a 85/15 mixture of diastereoisomers (2.1 g, 95%). The ^{1}H-NMR spectrum shows a keto-enol equilibrium, only the ketone form has been described.

$[\alpha]_D^{20}$ +7.6 (*c* 0.55, $CHCl_3$).

IR (ATR) ν (cm^{-1}): 2954, 2877, 1737, 1716, 1641, 1238, 1074, 1004, 835, 729.

Signals of the major and the minor diastereoisomers could not be assigned separately in both ^{1}H and ^{13}C NMR spectra.

^{1}H-NMR (400 MHz, $CDCl_3$) δ 7.35-7.26 (m, 5H, H_{23}+H_{24}+H_{25}), 5.98 (ddd, J = 17.4, 10.6, 5.7 Hz, 1H, H_{11}), 5.78 (m, 1H, H_8), 5.17-4.92 (m, 4H, H_9+H_{10}), 4.49 (s, 2H, H_{21}), 4.23 (m, 1H, H_5), 4.12 (m, 1H, H_7), 3.82 (m, 1H, H_{13}), 3.46 (t, J = 6.6 Hz, 2H, H_{18}), 3.40 (d, J = 15.7 Hz, 1H, H_2), 3.34 (d, J = 15.7 Hz, 1H, H_2), 2.66 (dd, J = 5.9, 3.9 Hz, 2H, H_4), 2.40 (m, 1H, H_{12}), 2.09-1.90 (m, 2H,

H_{14}+H_{16}), 1.84-1.59 (m, 6H, H_6+H_{14}+H_{16}+H_{17}), 1.50 (s, 3H, H_{19}), 0.97-0.90 (m, 21H, H_{20}+H_{27}+H_{29}), 0.88 (s, 9H, H_{32}), 0.62-0.54 (m, 12H, H_{26}+H_{28}), 0.06 (d, J = 5.7 Hz, 6H, H_{30}).

^{13}C-NMR (100 MHz, $CDCl_3$) δ 202.0 (s, C_3), 166.3 (s, C_1), 141.4 (d, C_8), 140.2 (d, C_{11}), 138.7 (s, C_{22}), 128.5 (d, C_{23} or C_{24}), 127.8 (d, C_{23} or C_{24}), 127.7 (d, C_{25}), 114.9 (t, C_9 or C_{10}), 114.4 (t, C_9 or C_{10}), 86.1 (s, C_{15}), 73.1 (t, C_{21}), 72.8 (d, C_{13}), 71.7 (d, C_7), 70.6 (t, C_{18}), 66.7 (d, C_5), 52.0 (t, C_2), 51.0 (t, C_4), 46.4 (d, C_{12}), 43.1 (t, C_6), 41.0 (t, C_{14}), 35.4 (t, C_{16}), 26.1 (q, C_{32}), 25.1 (q, C_{19}), 24.3 (t, C_{17}), 18.2 (s, C_{31}), 14.0 (q, C_{20}), 7.0 (q, C_{27}+C_{29}), 5.2 (t, C_{26}+C_{28}), −3.7 (q, C_{30}), −4.0 (q, C_{30}).

HRMS (ESI) *m/z*: calculated for $C_{45}H_{82}O_7Si_3$ $[M+Na]^+$: 841.52606, found: 841.52663.

(4*R*,6*R*,7*R*)-1-(Benzyloxy)-6-((*tert*-butyldimethylsilyl)oxy)-4,7-dimethylnon-8-en-4-yl 2-((2*S*,4*R*,6*R*)-4-hydroxy-2-methoxy-6-vinyltetrahydro-2H-pyran-2-yl)acetate (26)

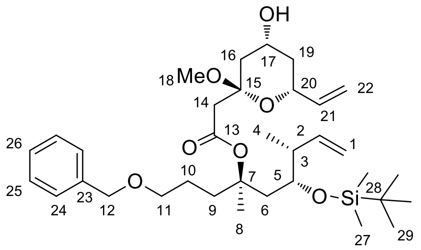

MW (g/mol): 604.90 **Molecular formula:** $C_{34}H_{56}O_7Si$

To a solution of **25** (1.0 g, 1.22 mmol) in MeOH (11.6 mL) was added pyridinium *p*-toluenesulfonate (153 mg, 0.61 mmol) followed by trimethoxymethane (400 µL, 3.66 mmol). After stirring overnigth at rt, the reaction mixture was diluted with Et_2O (30mL) and poured into a saturated aq. solution of $NaHCO_3$ (50 mL). The two layers were separated and the aqueous fraction was extracted twice with Et_2O (2 x 25 mL). The combined organic layers were washed with brine (10 mL), dried over Na_2SO_4, filtered, and concentrated under reduced pressure. The crude residue was finally purified by flash column chromatography over silica gel (PE/Et_2O = 80:20) to afford (4*R*,6*R*,7*R*)-1-(benzyloxy)-6-((*tert*-butyldimethylsilyl)oxy)-4,7-dimethylnon-8-en-4-yl 2-((2*S*,4*R*,6*R*)-4-hydroxy-2-methoxy-6-vinyltetrahydro-2H-pyran-2-yl)acetate (**26**) as a 87:13 mixture of diastereoisomers (610 mg, 83%).

$[\alpha]_D^{20}$ −6.0 (*c* 0.86, $CHCl_3$).

IR (ATR) ν (cm^{-1}): 3415, 2930, 2856, 1725, 1361, 1250, 1096, 1077, 915, 834, 773.

Signals of the major and the minor diastereoisomers could not be assigned separately in both ^{1}H and ^{13}C NMR spectra.

^{1}H-NMR (400 MHz, C_6D_6) δ 7.39-7.16 (m, 4H, H_{Ph}), 7.14-7.07 (m, 1H, H_{Ph}), 6.15 (ddd, J = 17.3, 10.7, 5.8 Hz, 1H, H_2), 5.79 (ddd, J = 17.3, 10.5, 5.5 Hz, 1H, H_{21}), 5.29-4.93 (m, 4H, H_1+H_{22}), 4.35 (s, 2H, H_{12}), 4.05 (m, 1H, H_{17}), 3.97 (m, 1H, H_5), 3.89 (m, 1H, H_{20}), 3.36 (t, J = 6.3 Hz, 2H, H_{11}), 3.11 (s, 3H, H_{18}), 2.72 (d, J = 13.7 Hz, 3H, H_{14}), 2.60-2.44 (m, 3H, H_3+H_{14}+H_{16}), 2.32 (dd, J = 14.9, 4.1 Hz, 1H, H_6), 2.16 (m, 1H, H_9), 1.91 (dd, J = 14.9, 7.2 Hz, 1H, H_6), 1.88-1.62 (m, 5H, H_9+H_{10}+H_{16}+H_{19}), 1.55 (s, 3H, H_8), 1.23 (m, 1H, H_{19}), 1.05 (d, J = 6.9 Hz, 3H, H_4), 0.99 (s, 9H, H_{29}), 0.15 (s, 3H, H_{27}), 0.11 (s, 3H, H_{27}).

^{13}C-NMR (100 MHz, C_6D_6) δ 168.2 (s, C_{13}), 140.6 (d, C_2), 139.3 (s, C_{23}), 138.7 (d, C_{21}), 128.6 (d, C_{24} or C_{25} or C_{26}), 127.9 (d, C_{24} or C_{25} or C_{26}), 127.7 (d, C_{24} or C_{25} or C_{26}), 114.6 (t, C_{11}), 114.6 (t, C_{22}), 100.0 (s, C_{15}), 84.6 (s, C_7), 73.3 (d, C_5), 73.1 (t, C_{12}), 70.6 (t, C_{11}), 70.4 (d, C_{20}), 64.5 (d, C_{17}), 47.8 (q, C_{18}), 43.54 (d, C_3), 43.49 (t, C_{14} or C_{16}), 43.2 (t, C_{14} or C_{16}), 41.7 (t, C_6), 40.6 (t, C_{19}), 36.2 (t, C_9), 26.3 (q, C_{29}), 25.1 (q, C_8), 24.7 (t, C_{10}), 18.4 (s, C_{28}), 14.1 (q, C_4), −3.6 (q, C_{27}), −3.9 (q, C_{27})

HRMS (ESI) *m/z*: calculated for $C_{34}H_{56}O_7Si$ $[M+Na]^+$: 627.36875, found: 627.36930.

(5*R*,7*R*,11*R*,13*R*)-5-(3-(Benzyloxy)propyl)-7-((*tert*-butyldimethylsilyl)oxy)-13-hydroxy-1-methoxy-5,8-dimethyl-4,15-dioxabicyclo[9.3.1]pentadec-9-en-3-one (27)

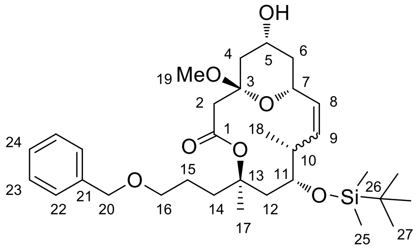

MW (g/mol): 576.84 **Molecular formula:** $C_{32}H_{52}O_7Si$

In a round-bottom flask, Hoveyda-Grubbs' second generation catalyst (53 mg, 10 mol %) was added to a solution of **26** (510 mg, 0.84 mmol) in toluene (270 mL). Benzoquinone (136 mg, 1.26 mmol) was then added and the resulting mixture was portioned in 10 sealed tubes and stirred overnight at 110 °C. The solvent was then removed under reduced pressure and the crude residue was purified by flash column chromatography over silica gel (PE/EtOAc = 95:5) to afford the macrocycle **27** as a 87/13 mixture of diastereoisomers (245 mg, 50%).

$[\alpha]_D^{20}$ −60 (*c* 0.50, $CHCl_3$).

IR (ATR) ν (cm^{-1}): 3403, 2956, 2856, 1725, 1248, 1097, 1034, 834, 774.

Signals of the major and the minor diastereoisomers could not be assigned separately in both ^{1}H and ^{13}C NMR spectra.

^{1}H-NMR (400 MHz, C_6D_6) δ 7.40-7.28 (m, 2H, H_{22} or H_{23}), 7.27-7.17 (m, 2H, H_{22} or H_{23}), 7.14-7.06 (m, 1H, H_{25}), 5.75 (m, 1H, H_9), 5.44 (dd, J = 11.3, 6.4 Hz, 1H, H_8), 4.32 (s br, 3H, H_{11}+H_{20}), 3.92 (m, 1H, H_5), 3.81 (ddd, J = 12.0, 6.4, 2.4 Hz, 1H, H_7), 3.32 (td, J = 6.2, 1.8 Hz, 2H, H_{16}), 3.09 (m, 1H, H_{10}), 2.86 (s, 3H, H_{19}), 2.81 (d, J = 12.8 Hz, 1H, H_2), 2.50 (dd, J = 14.8, 7.0 Hz, 1H, H_{12}), 2.22-2.10 (m, 2H, H_4+H_{14}), 2.06-1.95 (m, 2H, H_2+H_4), 1.85 (dd, J = 14.8, 3.5 Hz, 1H, H_{12}), 1.82-1.71 (m, 2H, H_6+H_{15}), 1.71-1.61 (m, 2H, H_{14}+H_{15}), 1.47 (s, 3H, H_{17}), 1.43-1.27 (m, 2H, H_6+OH), 1.10 (d, J = 6.7 Hz, 3H, H_{18}), 1.04 (s, 9H, H_{27}), 0.19 (s, 3H, H_{25}), 0.19 (s, 3H, H_{25}).

^{13}C-NMR (100 MHz, C_6D_6) δ 168.8 (s, C_1), 140.9 (d, C_9), 139.3 (s, C_{21}), 128.6 (d, C_{22} or C_{23} or C_{24}), 127.9 (d, C_{22} or C_{23} or C_{24}), 127.7 (d, C_{22} or C_{23} or C_{24}), 126.2 (d, C_8), 100.6 (s, C_3), 83.1 (s, C_{13}), 73.4 (d, C_{11}), 73.1 (t, C_{20}), 70.6 (t, C_{16}), 68.5 (d, C_7), 64.5 (d, C_5), 47.3 (q, C_{19}), 46.5 (t, C_{12}), 43.1 (t, C_2), 42.7 (t, C_4), 40.6 (t, C_6), 39.1 (d, C_{10}), 38.3 (t, C_{14}), 26.5 (q, C_{27}), 24.8 (q, C_{17}), 24.7 (t, C_{15}), 18.7 (s, C_{26}), 12.5 (q, C_{18}), −3.5 (q, C_{25}), −3.6 (q, C_{25}).

HRMS (ESI) *m/z*: calculated for $C_{32}H_{52}O_7Si$ $[M+Na]^+$: 599.33745, found: 599.33774.

(5*R*,7*R*,8*R*,11*S*,13*R*)-7-((*tert*-Butyldimethylsilyl)oxy)-13-hydroxy-5-(3-hydroxypropyl)-1-methoxy-5,8-dimethyl-4,15-dioxabicyclo[9.3.1]pentadecan-3-one (28)

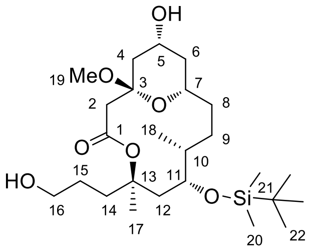

MW (g/mol): 488.73 **Molecular formula:** $C_{25}H_{48}O_7Si$

To a solution of **27** (200 mg, 0.35 mmol) in EtOH (7.8 mL) was added $Pd(OH)_2/C$ (10 % wt, 24 mg). After degasing the mixture under vacuum several times, the solution was then stirred 3 h under positive pressure of H_2. The catalyst was eventually filtered through a plug of Celite® and the solvent was evaporated under reduced pressure. The crude residue was purified by flash column chromatography over silica gel (PE/EtOAc = 70:30) to afford **28** as a 85:15 mixture of diastereoisomers (150 mg, 89%).

$[\alpha]_D^{20}$ −46.1 (*c* 0.50, $CHCl_3$).

IR (ATR) ν (cm^{-1}): 3360, 2954, 2926, 2855, 1726, 1462, 1249, 1039, 835, 772.

Major diastereoisomer: ^{1}H-NMR (400 MHz, C_6D_6) δ 4.49 (m, 1H, H_7), 3.97 (m, 1H, H_{11}), 3.55 (m, 1H, H_5), 3.35 (m, 2H, H_{16}), 2.93 (s, 3H, H_{19}), 2.72 (d, J = 12.4 Hz, 1H, H_2), 2.50 (dd, J = 14.8, 6.4 Hz, 1H, H_8), 2.12 (m, 1H, H_{14}), 2.06 (d, J = 12.4 Hz, 1H, H_2), 2.01-1.79 (m, 5H, H_4+H_8+H_9+H_{10}), 1.61-1.28 (m, 9H, H_4+H_6+H_{12}+H_{14}+H_{15}+OH), 1.44 (s, 3H, H_{17}), 1.05 (s, 9H, H_{22}), 1.00 (d, J = 6.7 Hz, 3H, H_{18}), 0.79 (s br, 1H, OH), 0.26 (s, 3H, H_{20}), 0.23 (s, 3H, H_{20}).
Minor diastereoisomer: ^{1}H-NMR (400 MHz, C_6D_6) δ 4.31 (m, 1H, H_7), 3.97 (m, 1H, H_{11}), 3.55 (m, 1H, H_5), 3.29 (m, 2H, H_{16}), 2.94 (s, 3H, H_{19}), 2.68 (d, J = 11.9 Hz, 1H, H_2), 2.50 (dd, J = 14.8, 6.4 Hz, 1H, H_8), 2.12 (m, 1H, H_{14}), 2.06 (d, J = 11.9 Hz, 1H, H_2), 2.01-1.79 (m, 5H, H_4+H_8+H_9+H_{10}), 1.61-1.28 (m, 9H, H_4+H_6+H_{12}+H_{14}+H_{15}+OH), 1.44 (s, 3H, H_{17}), 1.05 (s, 9H, H_{22}), 1.00 (d, J = 6.7 Hz, 3H, H_{18}), 0.79 (s br, 1H, OH), 0.15 (s, 3H, H_{20}), 0.13 (s, 3H, H_{20}).

Signals of the major and the minor diastereoisomers could not be assigned separately in ^{13}C NMR spectrum.
^{13}C-NMR (100 MHz, C_6D_6) δ 168.6 (s, C_1), 100.0 (s, C_3), 83.0 (s, C_{13}), 69.6 (d, C_5), 68.8 (d, C_7), 64.8 (d, C_{11}), 62.9 (t, C_{16}), 47.4 (q, C_{19}), 47.0 (t, C_8), 43.5 (t, C_2), 43.2 (t, C_9), 37.9 (t, C_{14}), 37.0 (d, C_{10}), 36.9 (t, C_{12}), 29.8 (t, C_4), 29.4 (t, C_6), 27.4 (t, C_{15}), 26.5 (t, C_{22}), 24.8 (q, C_{17}), 18.9 (s, C_{21}), 14.7 (q, C_{18}), −3.2 (q, C_{20}), −3.3 (q, C_{20}).
HRMS (ESI) m/z: calculated for $C_{25}H_{48}O_7Si$ $[M+Na]^+$: 511.30615, found: 511.30628.

(5*R*,7*R*,8*R*,11*S*,13*R*)-7-((*tert*-Butyldimethylsilyl)oxy)-13-hydroxy-1-methoxy-5,8-dimethyl-5-((3*E*,5*E*)-octa-3,5-dien-1-yl)-4,15-dioxabicyclo[9.3.1]pentadecan-3-one (31)

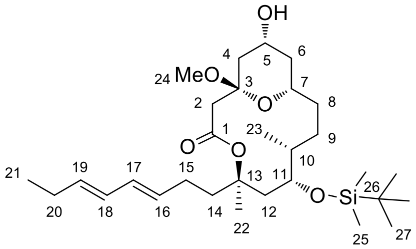

MW (g/mol): 538.83 **Molecular formula:** $C_{30}H_{54}O_6Si$

To a solution of alcohol (**28**) (140 mg, 0.29 mmol) in CH_2Cl_2 (2 mL) were added TEMPO (9 mg, 0.057 mmol) and BAIB (92 mg, 0.29 mmol). The resulting mixture was stirred for 2 h at rt and an additional portion of BAIB (45 mg, 0.15 mmol) was added. After 2 h at the same temperature, the solvent was removed under reduced pressure. The crude residue was filtered over a pad of silica (PE/EtOAc = 70:30) to afford the aldehyde (**29**), which was used in the next step without further purification (104 mg, 74%).

To a solution of (*E*)-bromotributyl(pent-2-en-1-yl)phosphorane **30** (90 mg, 0.26 mmol) in THF (0.4 mL) at −78 °C was added a solution of LiHMDS (1 M in THF), (230 µL, 0.023 mmol). The resulting mixture was stirred for 10 min at the same temperature and aldehyde **29** (25 mg, 51 µmol) diluted in THF (0.3 mL) was added dropwise. After 4 h at the same temperature, H_2O (5 mL) was poured into the reaction mixture. The two layers were separated and the aqueous fraction was extracted twice with EtOAc (2 x 10 mL). The combined organic layers were dried over Na_2SO_4, filtered and concentrated under reduced pressure. The crude residue was finally purified by flash column chromatography over silica gel (PE/EtOAc = 80:20) to afford (5*R*,7*R*,8*R*,11*S*,13*R*)-7-((*tert*-butyldimethylsilyl)oxy)-13-hydroxy-1-methoxy-5,8-dimethyl-5-((3*E*,5*E*)-octa-3,5-dien-1-yl)-4,15-dioxabicyclo[9.3.1]pentadecan-3-one **31** as a 90:10 mixture of diastereoisomers (10 mg, 36%).

$[\alpha]_D^{20}$ −39.4 (*c* 1.0, $CHCl_3$).
IR (ATR) *ν* (cm^{-1}): 3411, 2957, 2929, 1727, 1249, 1037, 989, 833, 772.
Major diastereoisomer: ^{1}H-NMR (400 MHz, C_6D_6) *δ* 6.23-5.97 (m, 2H, H_{17}+H_{18}), 5.71-5.44 (m, 2H, H_{16}+H_{19}), 4.49 (m, 1H, H_{11}), 3.97 (m, 1H, H_5), 3.55 (m, 1H, H_7), 2.94 (s, 3H, H_{24}), 2.73 (d, *J* = 12.4 Hz, 1H, H_2), 2.48 (dd, *J* = 14.7, 6.6 Hz, 1H, H_{12}), 2.31-2.10 (m, 3H, H_9+H_{15}), 2.07 (d, *J* = 12.4 Hz, 1H, H_2), 2.04-1.75 (m, 6H, H_4+H_8+H_{12}+H_{20}+OH), 1.70-1.49 (m, 3H, H_6+H_{14}+H_{15}), 1.46 (s, 3H, H_{22}), 1.38-1.29 (m, 3H, H_6+H_8+H_{14}), 1.04 (s, 9H, H_{27}), 1.00 (d, *J* = 6.7 Hz, 3H, H_{23}), 0.91 (t, *J* = 7.4 Hz, 3H, H_{21}), 0.26 (s, 3H, H_{25}), 0.21 (s, 3H, H_{25}).
Minor diastereoisomer: ^{1}H-NMR (400 MHz, C_6D_6) *δ* 6.23-5.97 (m, 2H, H_{17}+H_{18}), 5.71-5.44 (m, 2H, H_{16}+H_{19}), 4.32 (m, 1H, H_{11}), 3.97 (m, 1H, H_5), 3.37 (m, 1H, H_7), 2.94 (s, 3H, H_{24}), 2.68 (d, *J* = 11.9 Hz, 1H, H_2), 2.48 (dd, *J* = 14.7, 6.6 Hz, 1H, H_{12}), 2.31-2.10 (m, 3H, H_9+H_{15}), 2.07 (d, *J* = 12.4 Hz, 1H, H_2), 2.04-1.75 (m, 6H, H_4+H_8+H_{12}+H_{20}+OH), 1.70-1.49 (m, 3H, H_6+H_{14}+H_{15}), 1.46 (s, 3H, H_{22}), 1.38-1.29 (m, 3H, H_6+H_8+H_{14}), 1.04 (s, 9H, H_{27}), 1.00 (d, *J* = 6.7 Hz, 3H, H_{23}), 0.91 (t, *J* = 7.4 Hz, 3H, H_{21}), 0.15 (s, 3H, H_{25}), 0.14 (s, 3H, H_{25}).

Signals of the major and the minor diastereoisomers could not be assigned separately in ^{13}C NMR spectrum.
^{13}C-NMR (100 MHz, C_6D_6) *δ* 168.6 (s, C_1), 134.2 (d, C_{17} or C_{18}), 131.6 (d, C_{17} or C_{18}), 131.5 (d, C_{16} or C_{19}), 130.0 (d, C_{16} or C_{19}), 100.0 (s, C_3), 82.9 (s, C_{13}), 69.6 (d, C_7), 68.9 (d, C_{11}), 64.8 (d, C_5), 47.5 (q, C_{24}), 46.9 (t, C_{12}), 43.6 (t, C_2), 43.2 (t, C_4), 41.3 (t, C_{15}), 36.93 (t, C_6), 36.88 (d, C_{10}), 29.8 (t, C_{14}), 29.5 (t, C_8), 27.5 (t, C_9), 26.5 (q, C_{27}), 26.0 (t, C_{20}), 24.9 (q, C_{22}), 18.8 (q, C_{26}), 14.7 (q, C_{23}), 13.9 (q, C_{21}), −3.1 (q, C_{25}), −3.4 (q, C_{25}).
HRMS (ESI) *m/z*: calculated for $C_{30}H_{54}O_6Si$ $[M+Na]^+$: 561.35819, found: 561.35801.

(5*R*,7*R*,8*R*,11*S*,13*R*)-1,7,13-Trihydroxy-5,8-dimethyl-5-((3*E*,5*E*)-octa-3,5-dien-1-yl)-4,15-dioxabicyclo[9.3.1]pentadecan-3-one (32)

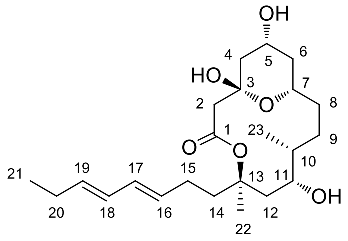

MW (g/mol): 410.54 **Molecular formula:** $C_{23}H_{38}O_6$

To a solution of **31** (10 mg, 0.18 mmol) in acetonitrile (1.2 mL) at 0 °C was added a solution of HF (in H_2O) (140 µL). After stirring 3 h at room temperature, a saturated aq. solution of $NaHCO_3$ (15 mL) was poured into the reaction mixture, a saturated aq. solution of $NaHCO_3$ (2 mL) was carefully added in order to quench the reaction. The reaction mixture was eventually extracted with EtOAc and the combined organic layers were dried over Na_2SO_4, filtered, and concentrated under reduced pressure. The crude residue was finally purified by flash column chromatography over silica gel (PE/EtOAc = 60:40) to afford (5*R*,7*R*,8*R*,11*S*,13*R*)-1,7,13-trihydroxy-5,8-dimethyl-5-((3*E*,5*E*)-octa-3,5-dien-1-yl)-4,15-dioxabicyclo[9.3.1]pentadecan-3-one (**32**) as a single diastereoisomers (6 mg, 79%).

^{1}H-NMR (400 MHz, C_6D_6) δ 6.17-5.86 (m, 2H, H_{17}+H_{18}), 5.73-5.46 (m, 2H, H_{16}+H_{19}), 4.15 (m, 1H, H_5), 3.96 (m, 2H, H_7+H_{11}), 2.63 (d, *J* = 13.4 Hz, 1H, H_2), 2.54 (d, *J* = 13.3 Hz, 1H, H_2), 2.26-1.99 (m, 6H, H_4+H_{15}+H_{20}+OH), 1.87 (m, 1H, H_6), 1.78-1.49 (m, 7H, H_9+H_{10}+H_{12}+H_{14}), 1.55 (s, 3H, H_{22}), 1.39-1.25 (m, 6H, H_4+H_6+H_8+2 OH), 0.99 (t, *J* = 7.5 Hz, 3H, H_{21}), 0.80 (d, *J* = 7.0 Hz, 3H, H_{23}).

^{13}C-NMR (100 MHz, C_6D_6) δ 134.7 (d, C_{19}), 131.2 (d, C_{16}), 131.0 (d, C_{17} or C_{18}), 129.3 (d, C_{17} or C_{18}), 97.3 (s, C_3), 87.1 (s, C_{13}), 71.2 (d, C_7 or C_{11}), 64.6 (d, C_5), 44.5 (t, C_2), 43.7 (t, C_4), 41.2 (t, C_6), 40.1 (s, C_{10}), 34.4 (t, C_{14}), 29.8 (t, C_8), 26.9 (t, C_{20}), 25.7 (t, C_{15}), 13.9 (q, C_{23}), 13.8 (q, C_{21}).

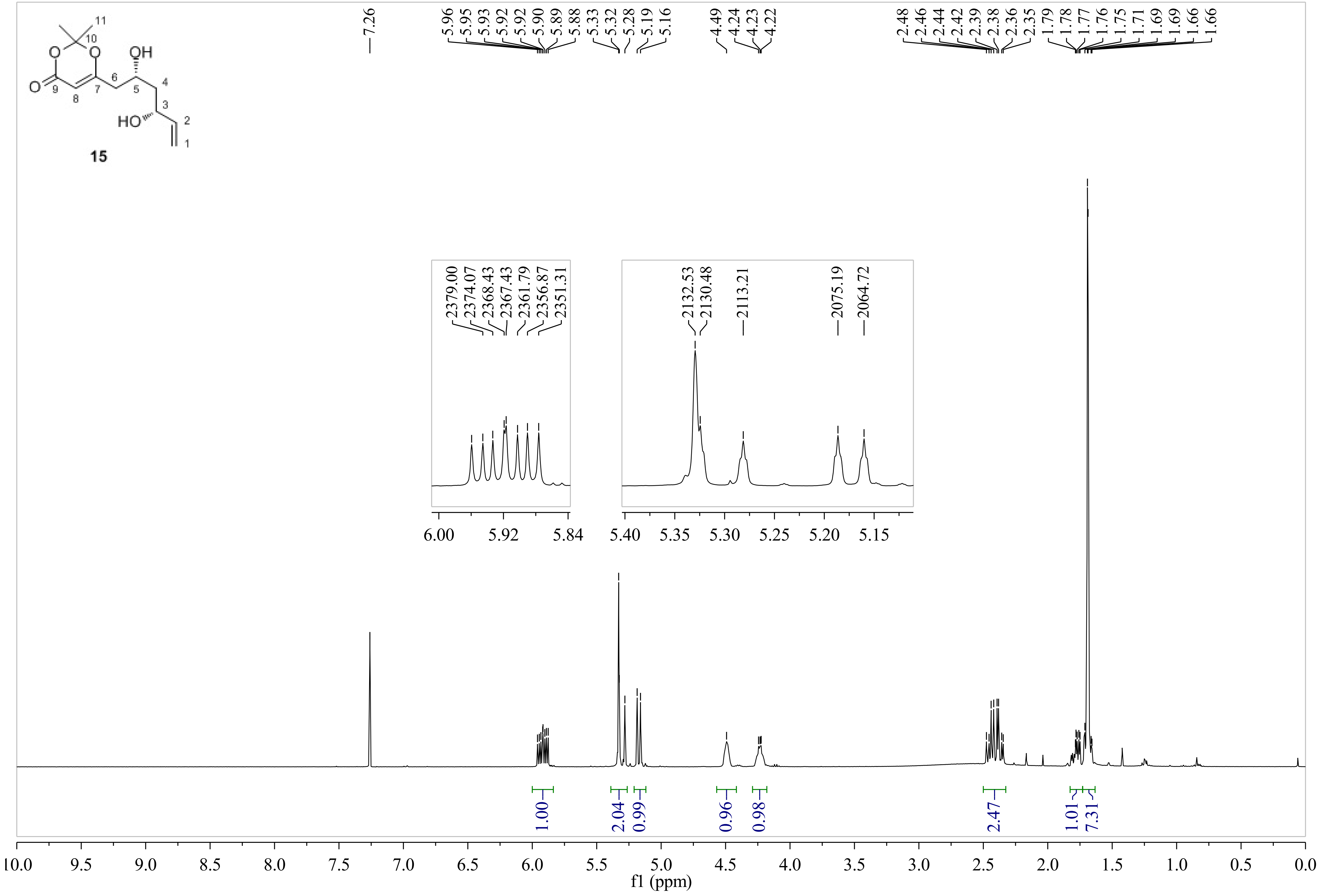
15
7.26
5.96 5.95 5.93 5.92 5.92 5.90 5.89 5.88 5.33 5.32 5.28 5.19 5.16
4.49 4.24 4.23 4.22
2.48 2.46 2.44 2.42 2.39 2.38 2.36 2.35 1.79 1.78 1.77 1.76 1.75 1.71 1.69 1.69 1.66 1.66
2379.00 2374.07 2368.43 2367.43 2361.79 2356.87 2351.31
2132.53 2130.48 2113.21 2075.19 2064.72
1.00 2.04 0.99 0.96 0.98 2.47 1.01 7.31
f1 (ppm)

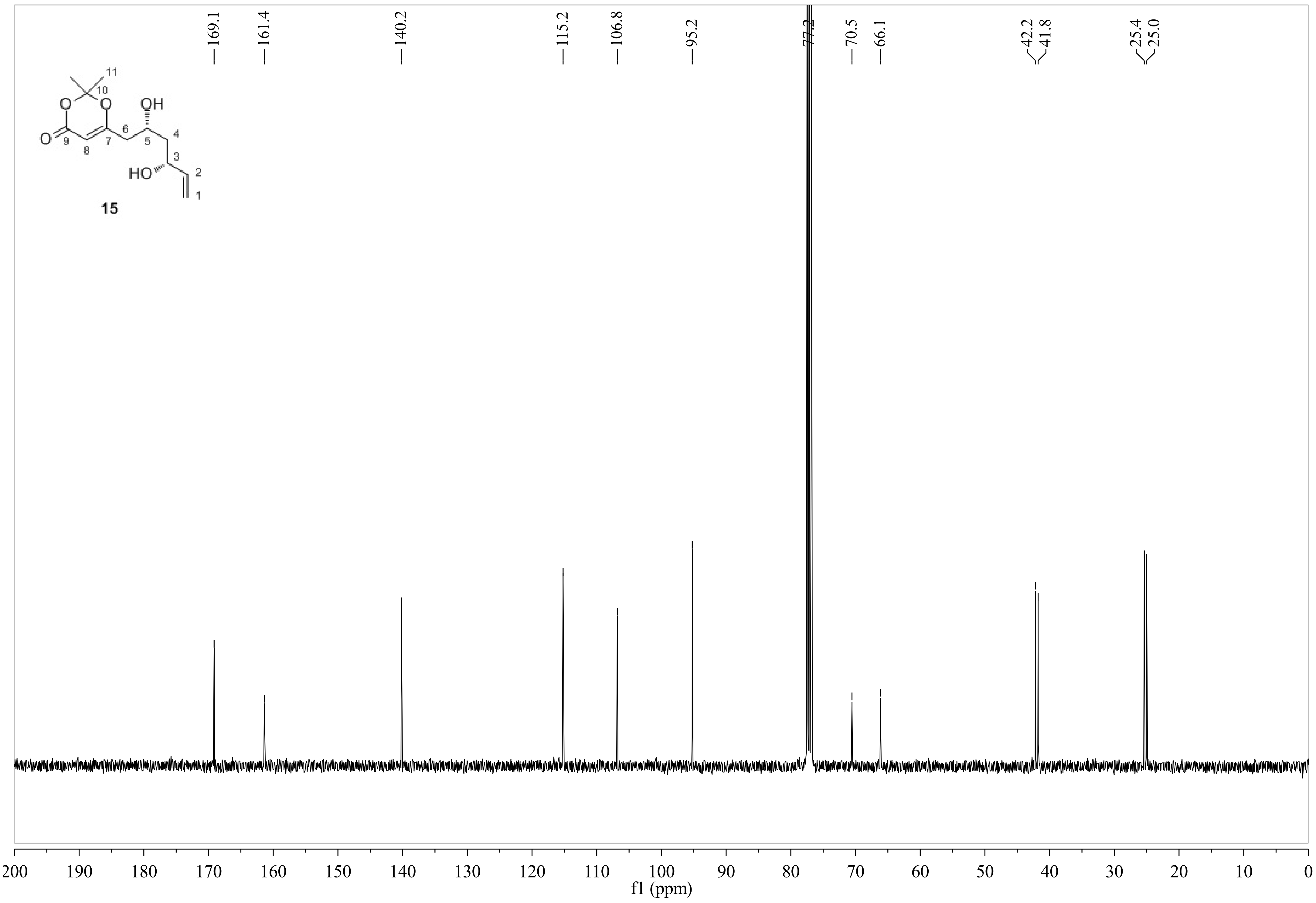
169.1
161.4
140.2
115.2
106.8
95.2
77.2
70.5
66.1
42.2
41.8
25.4
25.0
OH
HO
O
1
2
3
4
5
6
7
8
9
10
11
15
200
190
180
170
160
150
140
130
120
110
100
90
80
70
60
50
40
30
20
10
0
f1 (ppm)

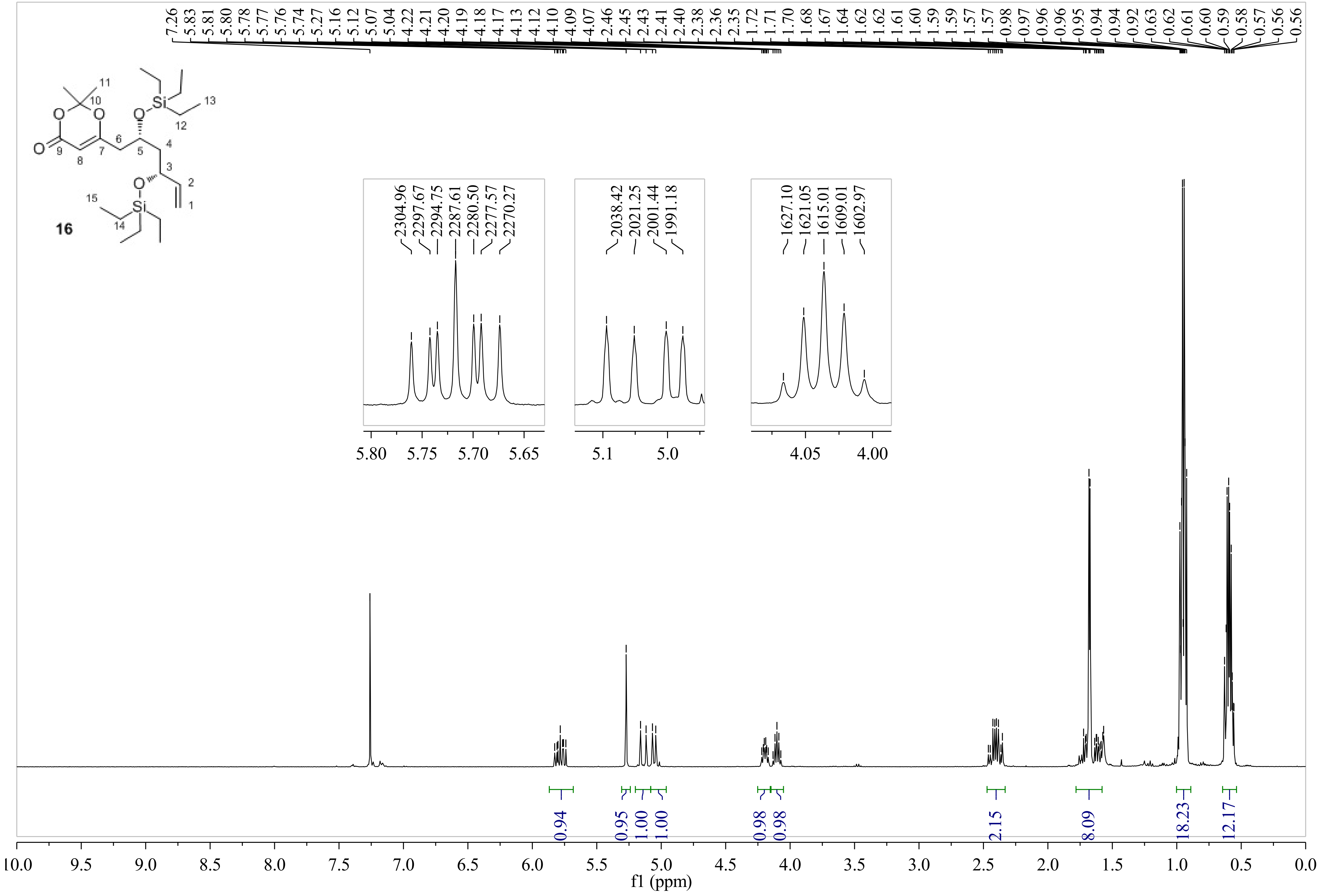

16
7.26 5.83 5.81 5.80 5.78 5.77 5.76 5.74 5.27 5.16 5.12 5.07 5.04 4.22 4.21 4.20 4.19 4.18 4.17 4.13 4.12 4.10 4.09 4.07 2.46 2.45 2.43 2.41 2.40 2.38 2.36 2.35 1.72 1.71 1.70 1.68 1.67 1.64 1.62 1.62 1.61 1.60 1.59 1.59 1.57 1.57 0.98 0.97 0.96 0.96 0.95 0.94 0.94 0.92 0.63 0.62 0.61 0.60 0.59 0.58 0.57 0.56 0.56
2304.96 2297.67 2294.75 2287.61 2280.50 2277.57 2270.27
2038.42 2021.25 2001.44 1991.18
1627.10 1621.05 1615.01 1609.01 1602.97
0.94 0.95 1.00 1.00 0.98 0.98 2.15 8.09 18.23 12.17
f1 (ppm)

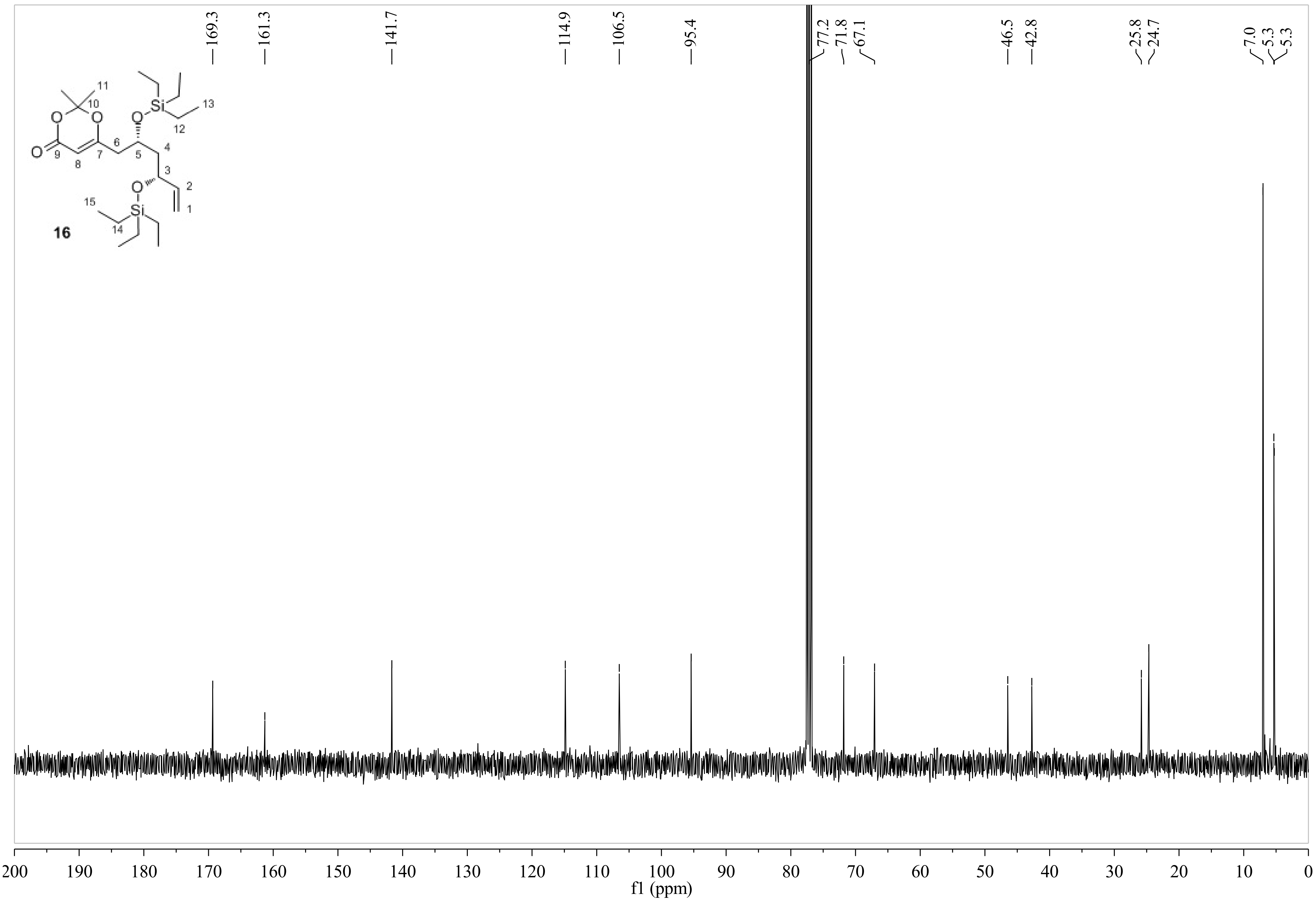
16
169.3
161.3
141.7
114.9
106.5
95.4
77.2
71.8
67.1
46.5
42.8
25.8
24.7
7.0
5.3
5.3
200 190 180 170 160 150 140 130 120 110 100 90 80 70 60 50 40 30 20 10 0
f1 (ppm)

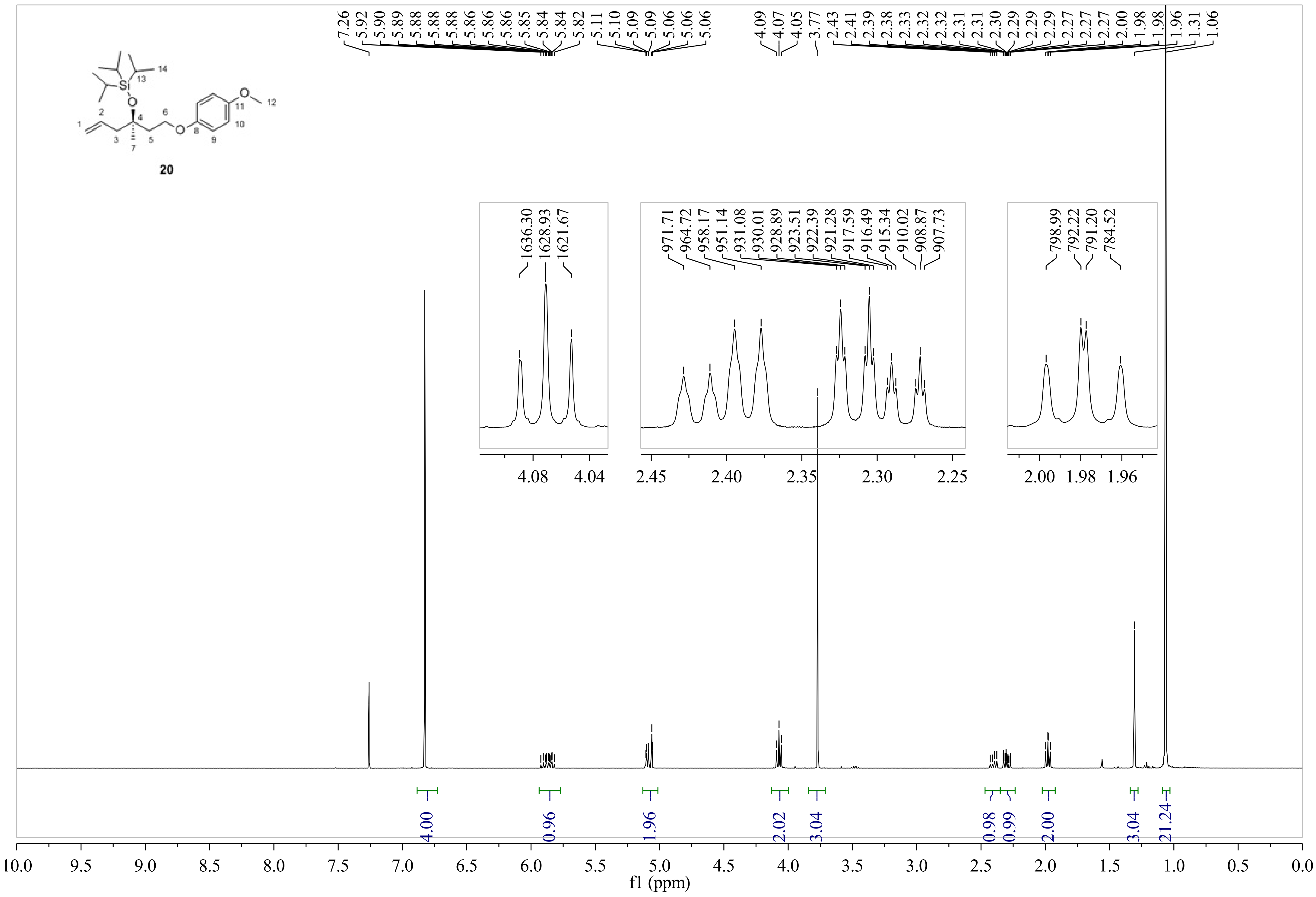

20
7.26 5.92 5.90 5.89 5.88 5.88 5.88 5.86 5.86 5.86 5.85 5.84 5.84 5.82 5.11 5.10 5.09 5.09 5.06 5.06 5.06 4.09 4.07 4.05 3.77 2.43 2.41 2.39 2.38 2.33 2.32 2.32 2.31 2.31 2.30 2.29 2.29 2.29 2.27 2.27 2.27 2.00 1.98 1.98 1.96 1.31 1.06
1636.30 1628.93 1621.67
971.71 964.72 958.17 951.14 931.08 930.01 928.89 923.51 922.39 921.28 917.59 916.49 915.34 910.02 908.87 907.73
798.99 792.22 791.20 784.52
4.08 4.04
2.45 2.40 2.35 2.30 2.25
2.00 1.98 1.96
4.00 0.96 1.96 2.02 3.04 0.98 0.99 2.00 3.04 21.24
10.0 9.5 9.0 8.5 8.0 7.5 7.0 6.5 6.0 5.5 5.0 4.5 4.0 3.5 3.0 2.5 2.0 1.5 1.0 0.5 0.0
f1 (ppm)

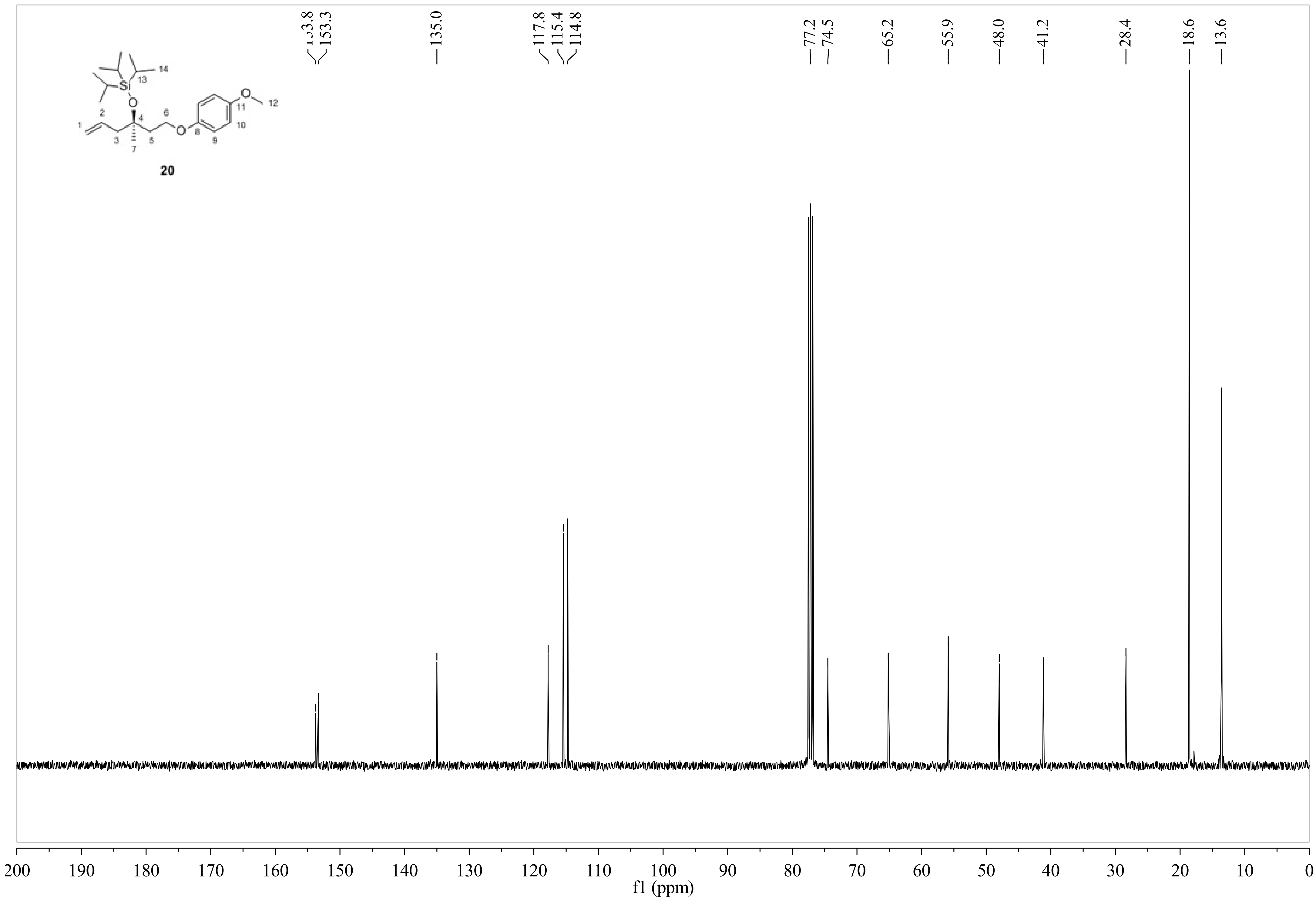

20
153.8
153.3
135.0
117.8
115.4
114.8
77.2
74.5
65.2
55.9
48.0
41.2
28.4
18.6
13.6
f1 (ppm)

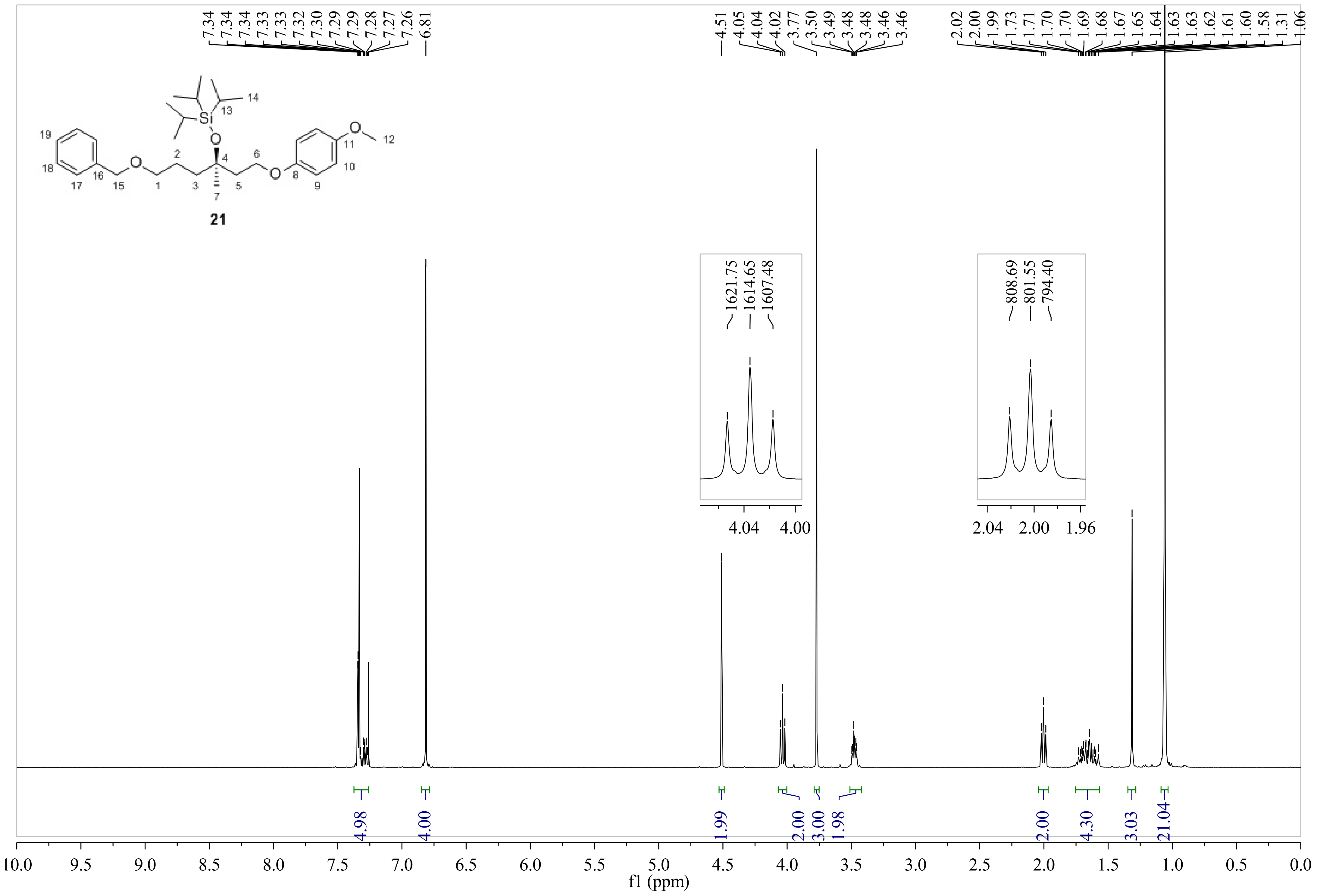
7.34
7.34
7.34
7.33
7.33
7.32
7.30
7.29
7.29
7.28
7.27
7.26
6.81
4.51
4.05
4.04
4.02
3.77
3.50
3.49
3.48
3.48
3.46
3.46
2.02
2.00
1.99
1.73
1.71
1.70
1.70
1.69
1.68
1.67
1.65
1.64
1.63
1.63
1.62
1.61
1.60
1.58
1.31
1.06
21
1621.75
1614.65
1607.48
808.69
801.55
794.40
4.98
4.00
1.99
2.00
3.00
1.98
2.00
4.30
3.03
21.04
f1 (ppm)

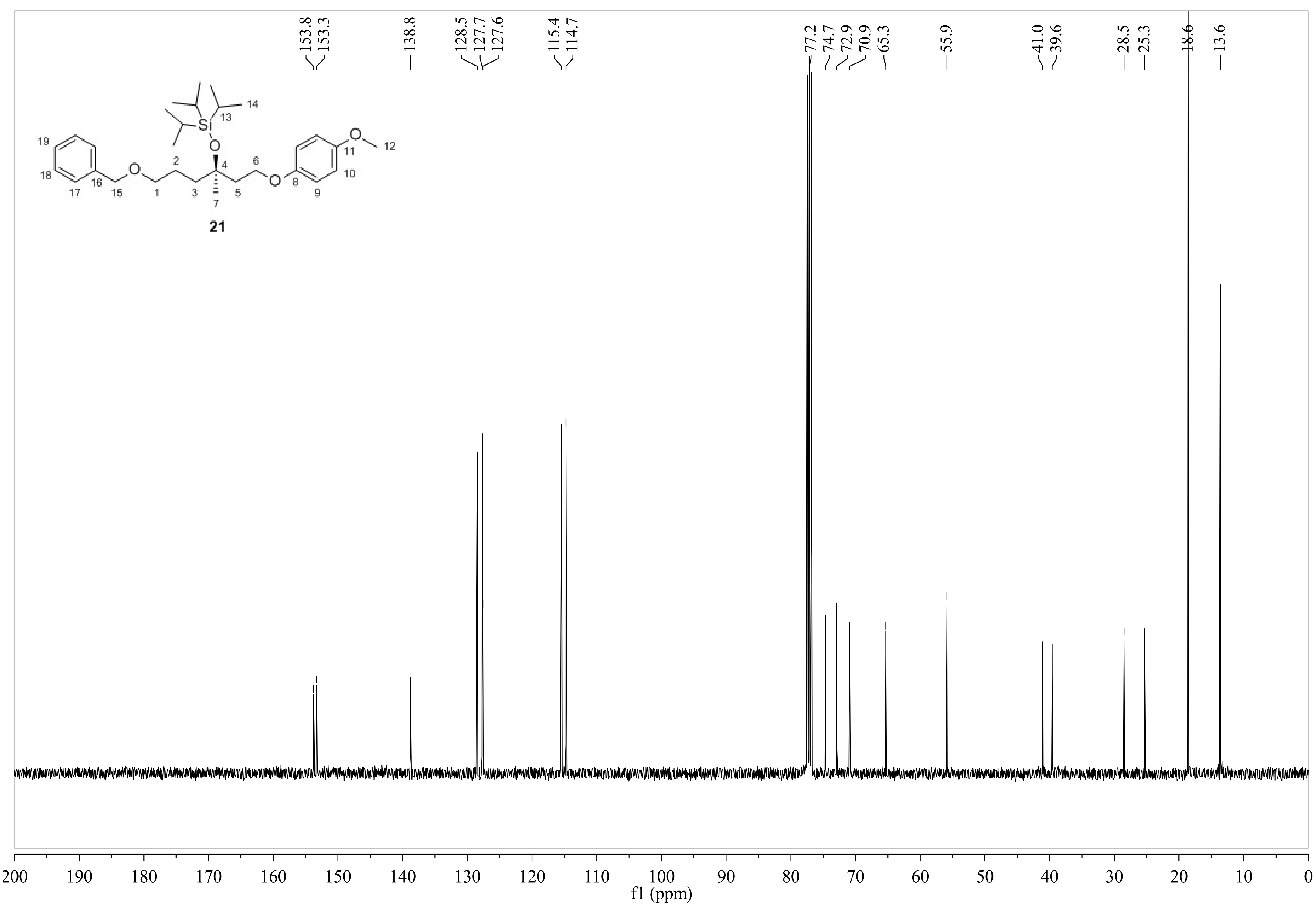
153.8
153.3
138.8
128.5
127.7
127.6
115.4
114.7
77.2
74.7
72.9
70.9
65.3
55.9
41.0
39.6
28.5
25.3
18.6
13.6
21
f1 (ppm)

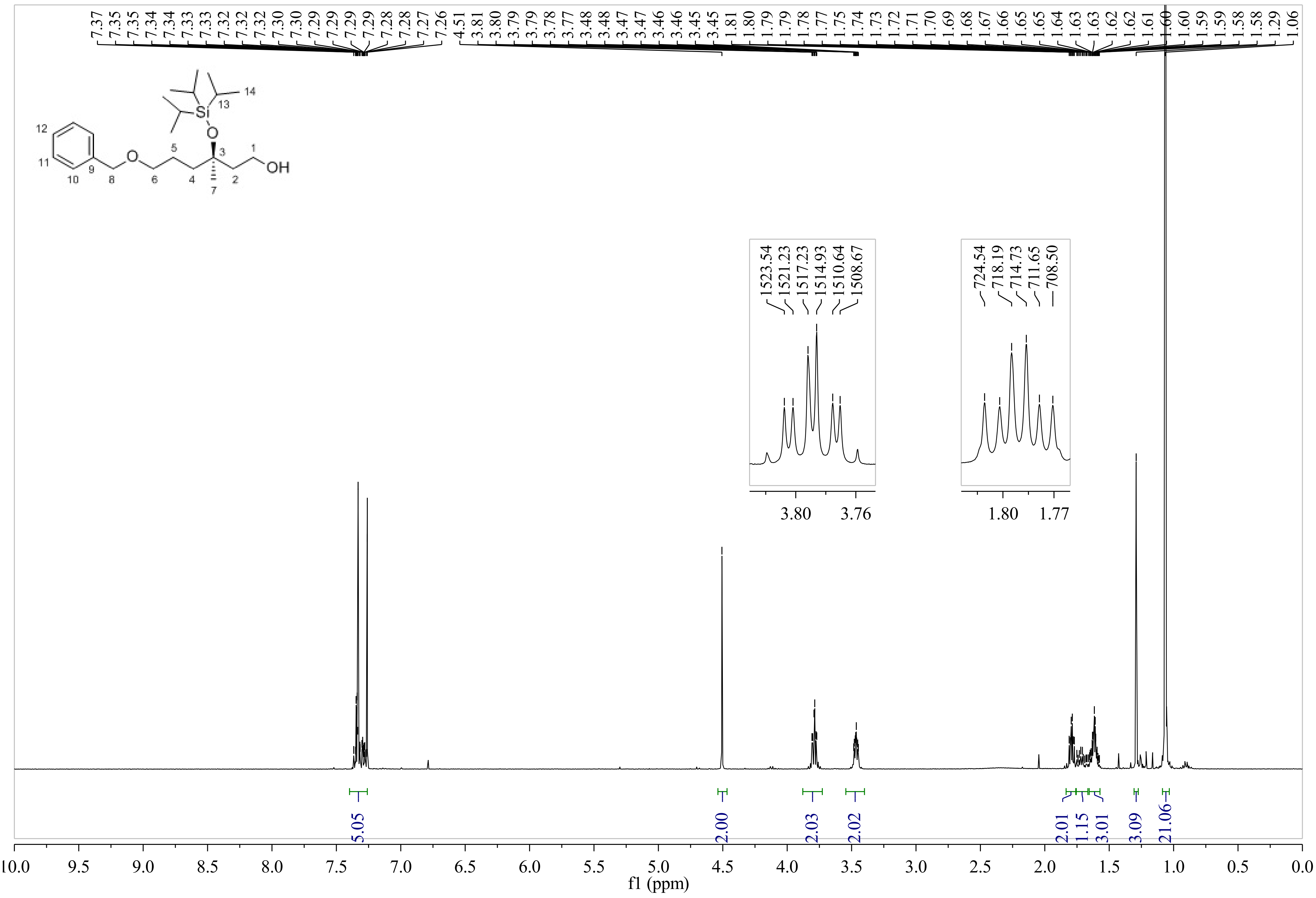

7.37 7.35 7.35 7.34 7.34 7.33 7.33 7.32 7.32 7.32 7.30 7.30 7.29 7.29 7.29 7.29 7.28 7.28 7.27 7.26
4.51 3.81 3.80 3.79 3.79 3.78 3.77 3.48 3.48 3.47 3.47 3.46 3.46 3.45 3.45 1.81 1.80 1.79 1.79 1.78 1.77 1.75 1.74 1.73 1.72 1.71 1.70 1.69 1.68 1.67 1.66 1.65 1.65 1.64 1.63 1.63 1.62 1.62 1.61 1.60 1.60 1.59 1.59 1.58 1.58 1.29 1.06
OH
Si
O
1 2 3 4 5 6 7 8 9 10 11 12 13 14
1523.54 1521.23 1517.23 1514.93 1510.64 1508.67
3.80 3.76
724.54 718.19 714.73 711.65 708.50
1.80 1.77
5.05 2.00 2.03 2.02 2.01 1.15 3.01 3.09 21.06
10.0 9.5 9.0 8.5 8.0 7.5 7.0 6.5 6.0 5.5 5.0 4.5 4.0 3.5 3.0 2.5 2.0 1.5 1.0 0.5 0.0
f1 (ppm)

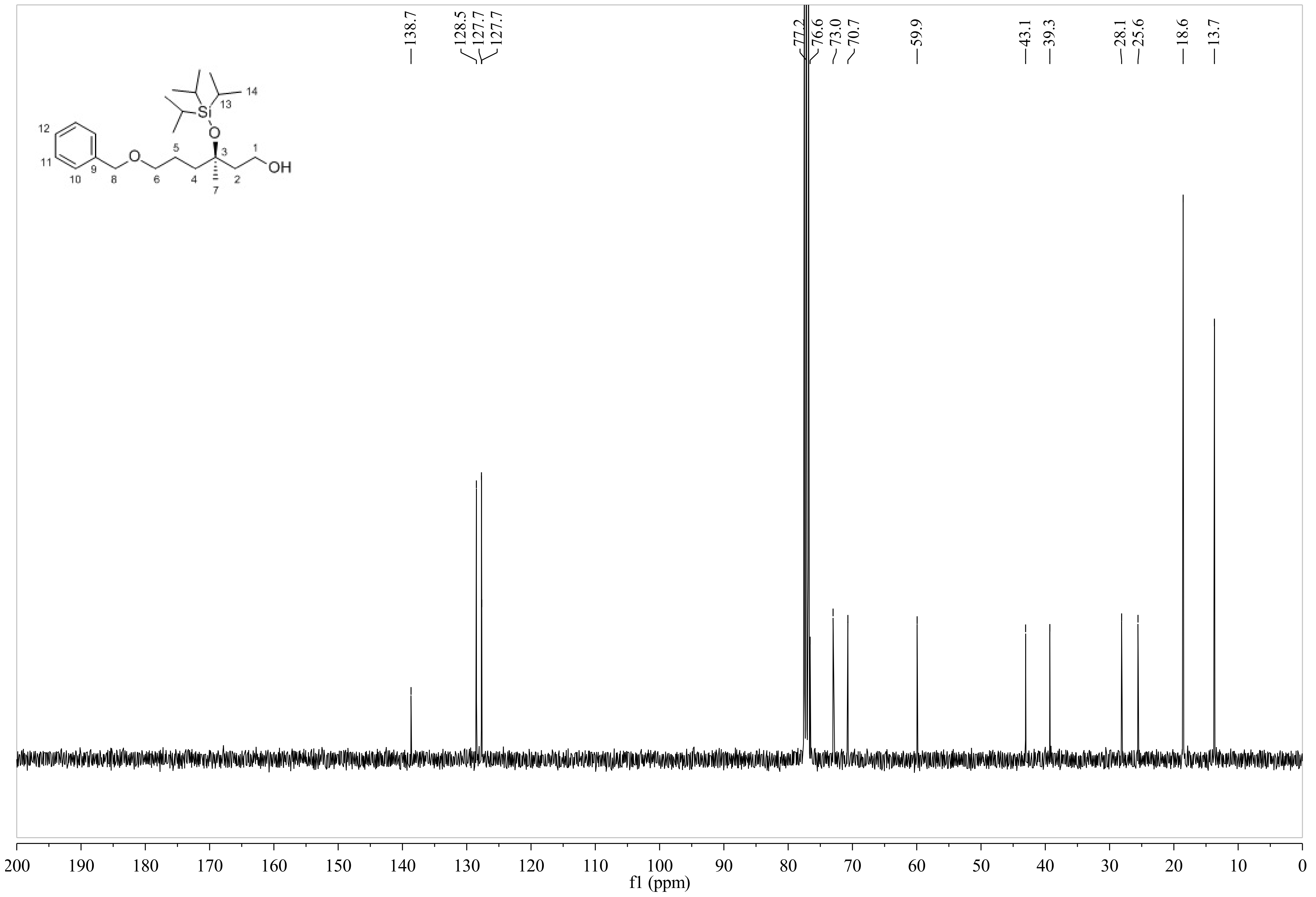

138.7
128.5
127.7
127.7
77.2
76.6
73.0
70.7
59.9
43.1
39.3
28.1
25.6
18.6
13.7
OH
O
Si
1
2
3
4
5
6
7
8
9
10
11
12
13
14
200
190
180
170
160
150
140
130
120
110
100
90
80
70
60
50
40
30
20
10
0
f1 (ppm)

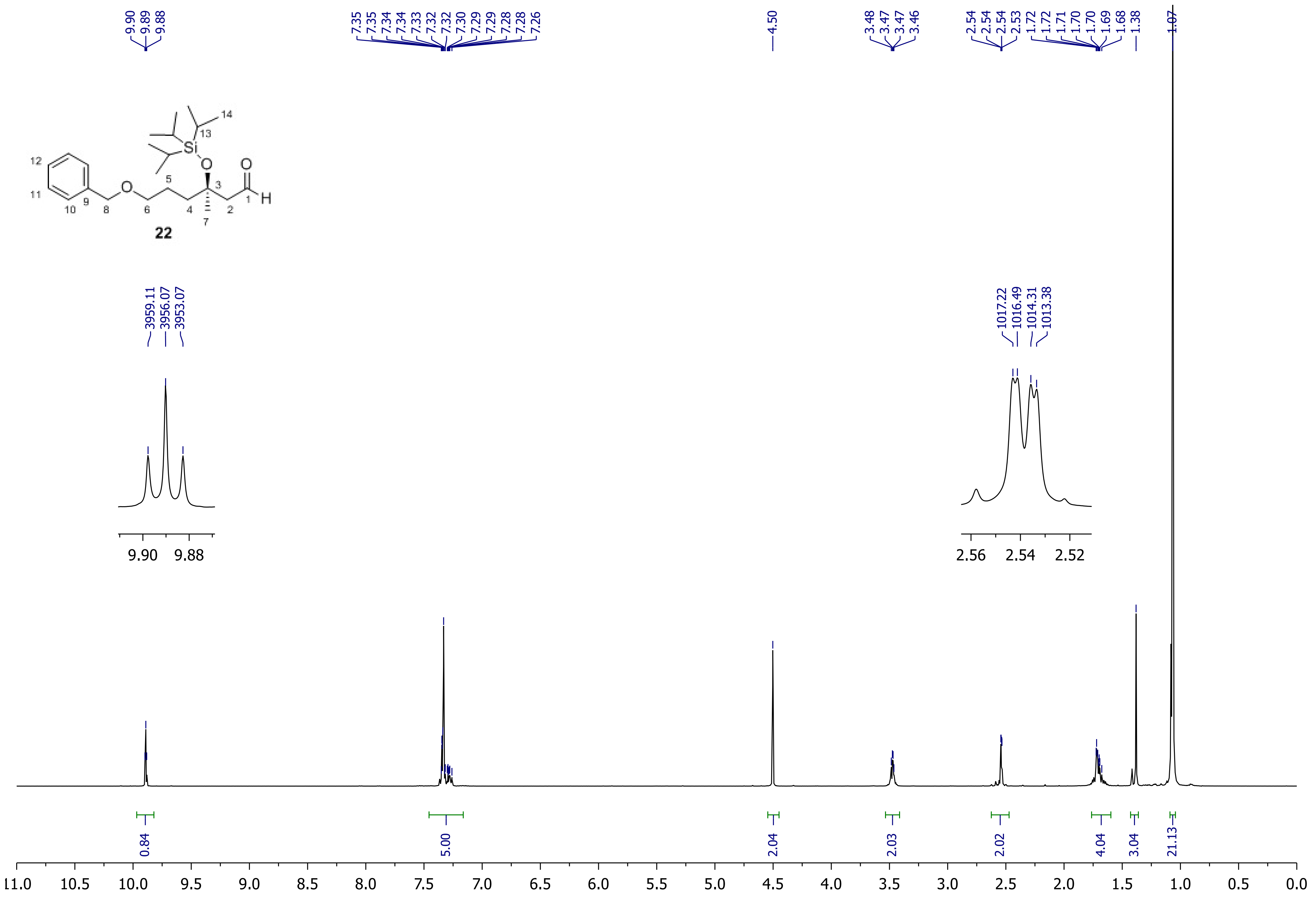

9.90
9.89
9.88
7.35
7.35
7.34
7.34
7.33
7.32
7.32
7.30
7.29
7.29
7.28
7.28
7.26
4.50
3.48
3.47
3.47
3.46
2.54
2.54
2.54
2.53
1.72
1.72
1.71
1.70
1.70
1.69
1.68
1.38
1.07
22
3959.11
3956.07
3953.07
9.90
9.88
1017.22
1016.49
1014.31
1013.38
2.56
2.54
2.52
0.84
5.00
2.04
2.03
2.02
4.04
3.04
21.13
11.0
10.5
10.0
9.5
9.0
8.5
8.0
7.5
7.0
6.5
6.0
5.5
5.0
4.5
4.0
3.5
3.0
2.5
2.0
1.5
1.0
0.5
0.0

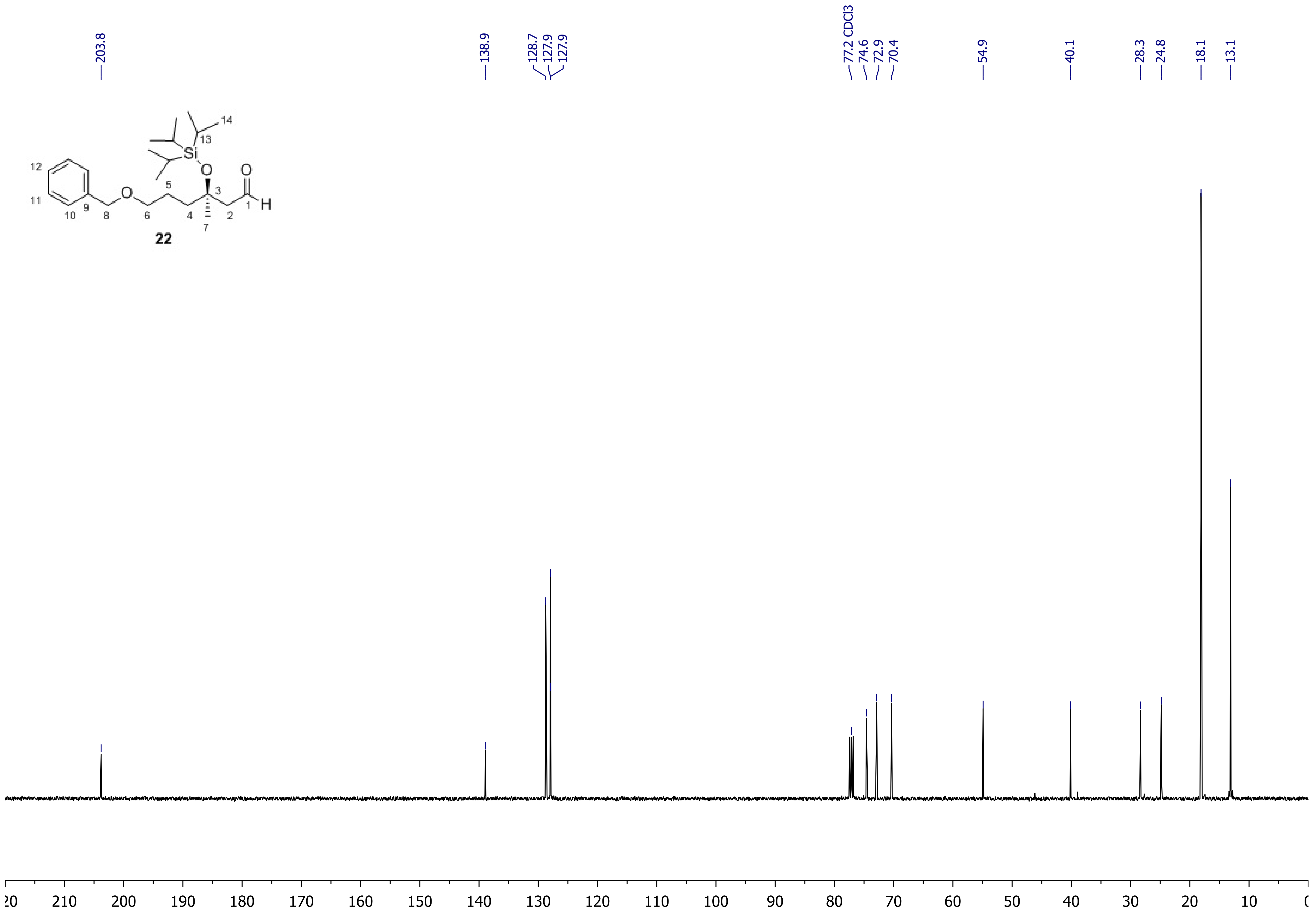

203.8
138.9
128.7
127.9
127.9
77.2 CDCl3
74.6
72.9
70.4
54.9
40.1
28.3
24.8
18.1
13.1
22
210
200
190
180
170
160
150
140
130
120
110
100
90
80
70
60
50
40
30
20
10

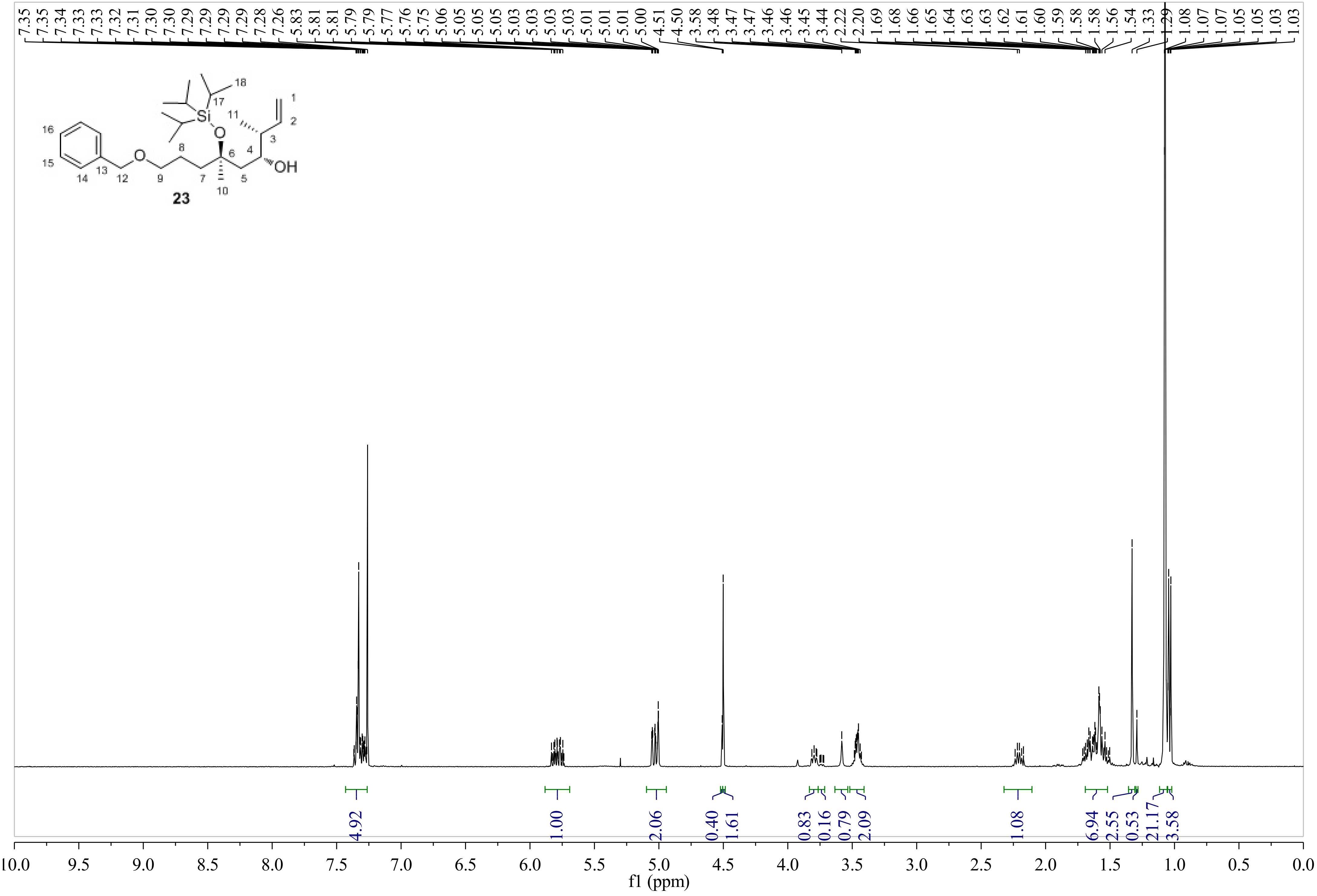

7.35
7.35
7.34
7.33
7.33
7.32
7.31
7.30
7.30
7.29
7.29
7.29
7.29
7.29
7.28
7.26
5.83
5.81
5.81
5.79
5.79
5.77
5.76
5.75
5.06
5.05
5.05
5.05
5.03
5.03
5.03
5.03
5.01
5.01
5.01
5.00
4.51
4.50
3.58
3.48
3.47
3.47
3.46
3.46
3.45
3.44
2.22
2.20
1.69
1.68
1.66
1.65
1.64
1.63
1.63
1.62
1.61
1.60
1.59
1.58
1.58
1.56
1.54
1.33
1.29
1.08
1.07
1.07
1.05
1.05
1.03
1.03
4.92
1.00
2.06
0.40
1.61
0.83
0.16
0.79
2.09
1.08
6.94
2.55
0.53
21.17
3.58
10.0
9.5
9.0
8.5
8.0
7.5
7.0
6.5
6.0
5.5
5.0
4.5
4.0
3.5
3.0
2.5
2.0
1.5
1.0
0.5
0.0
f1 (ppm)
1
2
3
4
5
6
7
8
9
10
11
12
13
14
15
16
17
18
Si
O
O
OH

23

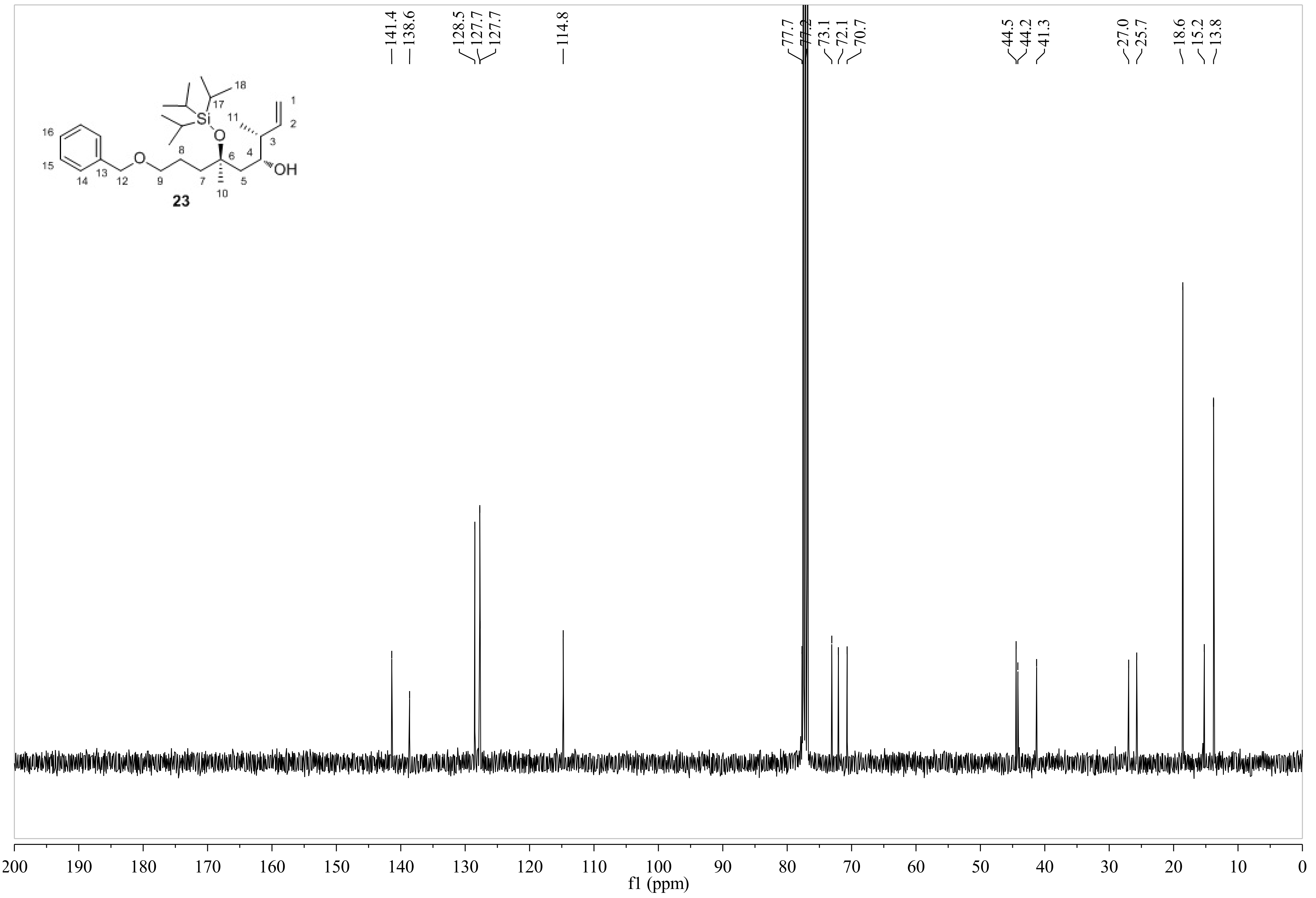
141.4
138.6
128.5
127.7
127.7
114.8
77.7
77.2
73.1
72.1
70.7
44.5
44.2
41.3
27.0
25.7
18.6
15.2
13.8
23
OH
O
Si
1
2
3
4
5
6
7
8
9
10
11
12
13
14
15
16
17
18
200
190
180
170
160
150
140
130
120
110
100
90
80
70
60
50
40
30
20
10
0
f1 (ppm)

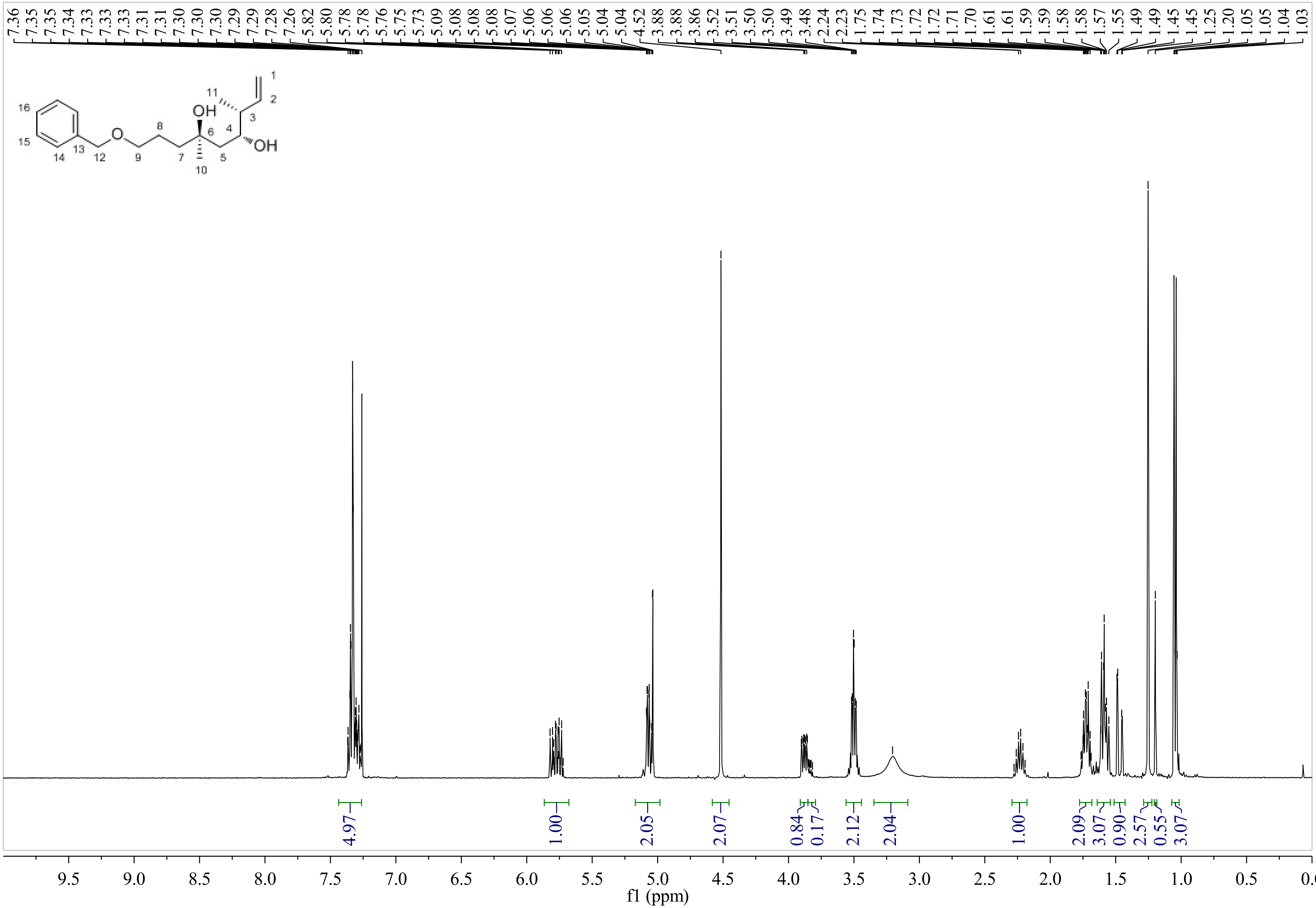
7.36 7.35 7.35 7.34 7.33 7.33 7.33 7.31 7.31 7.30 7.30 7.30 7.29 7.29 7.28 7.26 5.82 5.80 5.78 5.78 5.76 5.75 5.73 5.09 5.08 5.08 5.08 5.07 5.06 5.06 5.06 5.05 5.04 5.04 4.52 3.88 3.88 3.86 3.52 3.51 3.50 3.50 3.49 3.48 2.24 2.23 1.75 1.74 1.73 1.72 1.72 1.71 1.70 1.61 1.61 1.59 1.59 1.58 1.58 1.57 1.55 1.49 1.49 1.45 1.45 1.25 1.20 1.05 1.05 1.04 1.03
OH
OH
O
1 2 3 4 5 6 7 8 9 10 11 12 13 14 15 16
4.97 1.00 2.05 2.07 0.84 0.17 2.12 2.04 1.00 2.09 3.07 0.90 2.57 0.55 3.07
9.5 9.0 8.5 8.0 7.5 7.0 6.5 6.0 5.5 5.0 4.5 4.0 3.5 3.0 2.5 2.0 1.5 1.0 0.5 0.0
f1 (ppm)

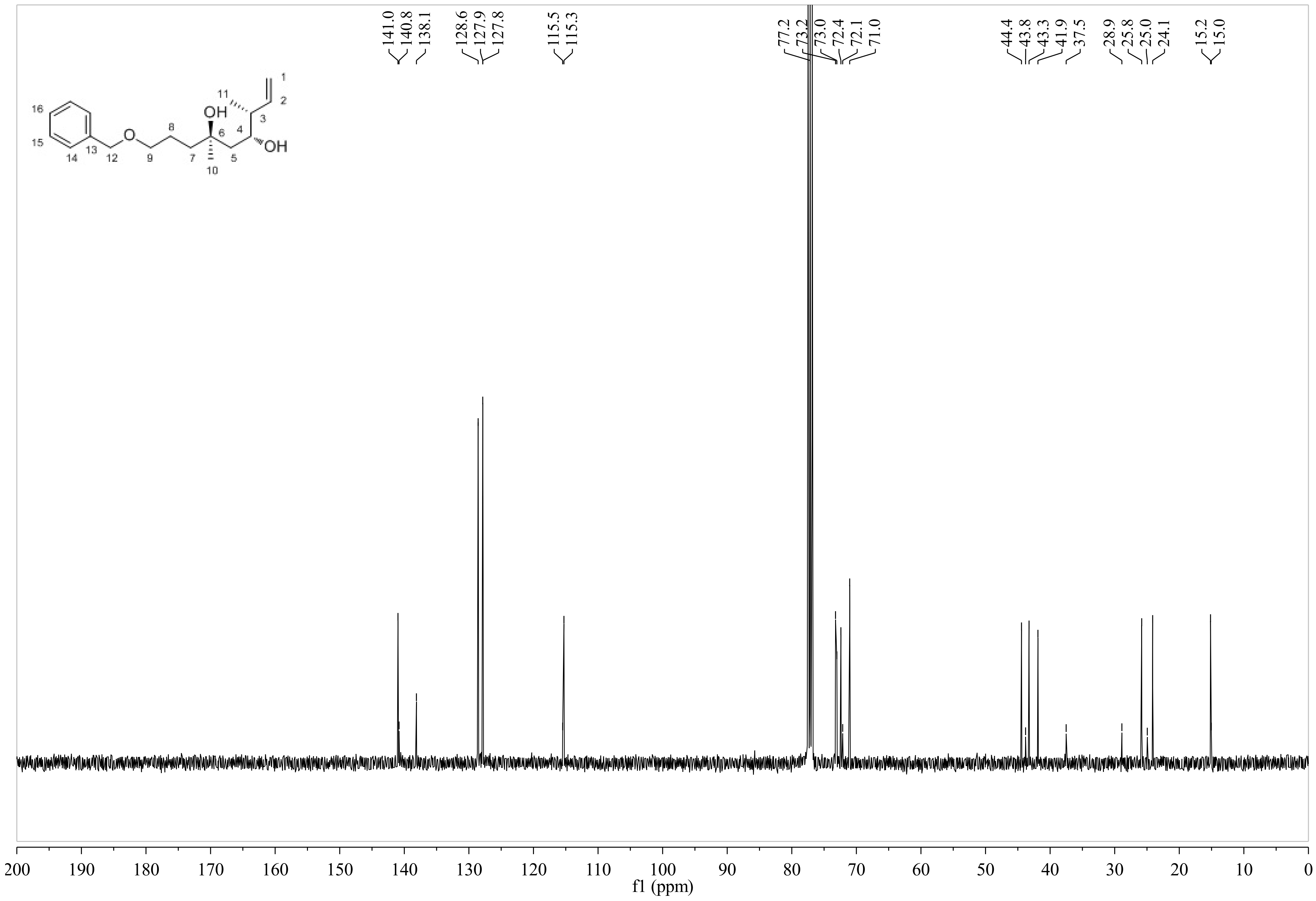
141.0
140.8
138.1
128.6
127.9
127.8
115.5
115.3
77.2
73.2
73.0
72.4
72.1
71.0
44.4
43.8
43.3
41.9
37.5
28.9
25.8
25.0
24.1
15.2
15.0
OH
OH
O
f1 (ppm)

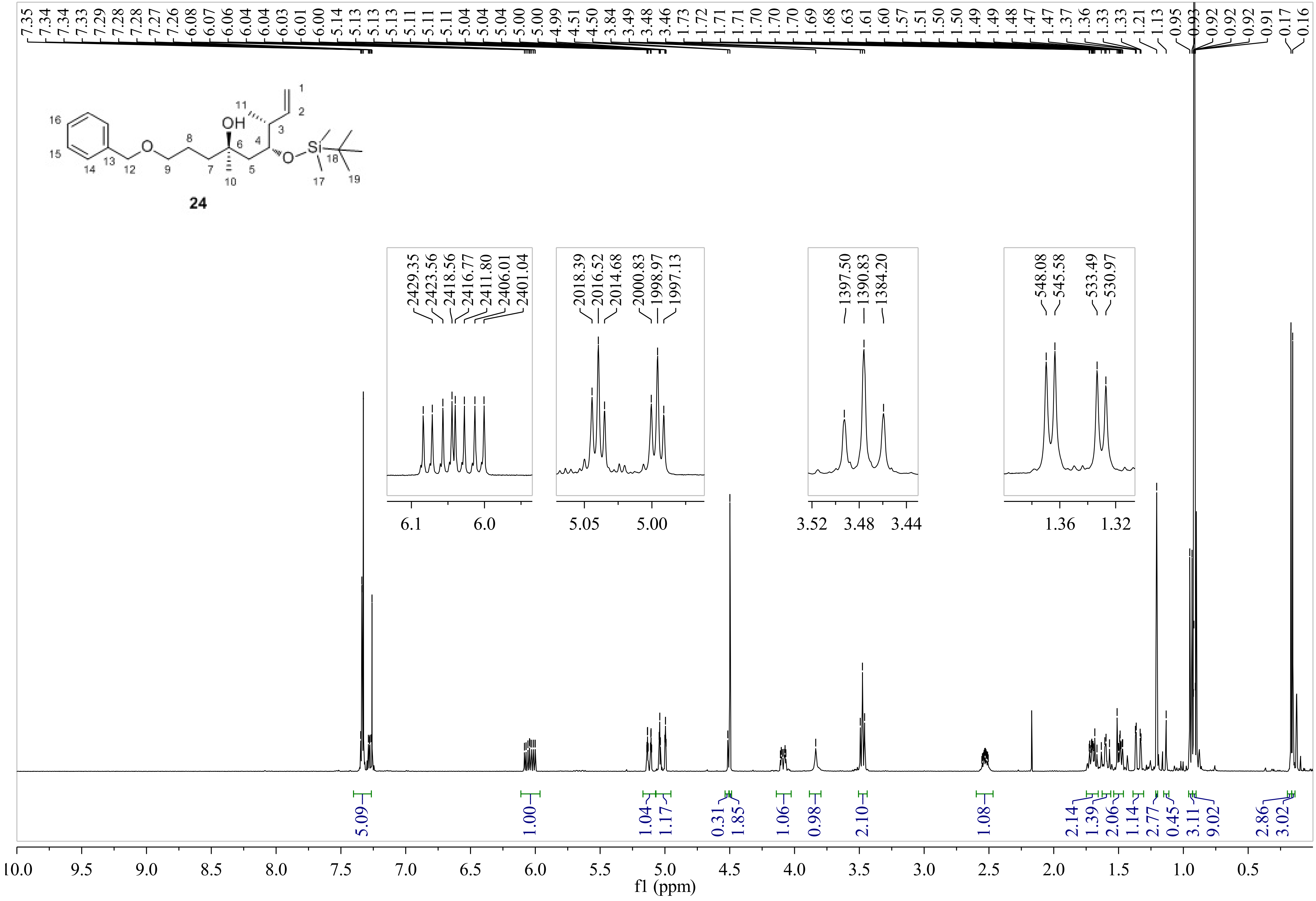

24
7.35 7.34 7.34 7.33 7.29 7.28 7.28 7.27 7.26 6.08 6.07 6.06 6.04 6.04 6.03 6.01 6.00 5.14 5.13 5.13 5.13 5.11 5.11 5.11 5.04 5.04 5.04 5.00 5.00 4.99 4.51 4.50 3.84 3.49 3.48 3.46 1.73 1.72 1.71 1.71 1.70 1.70 1.70 1.69 1.68 1.63 1.61 1.60 1.57 1.51 1.50 1.50 1.49 1.49 1.48 1.47 1.47 1.37 1.36 1.33 1.33 1.21 1.13 0.95 0.93 0.92 0.92 0.92 0.91 0.17 0.16
2429.35 2423.56 2418.56 2416.77 2411.80 2406.01 2401.04
2018.39 2016.52 2014.68 2000.83 1998.97 1997.13
1397.50 1390.83 1384.20
548.08 545.58 533.49 530.97
5.09 1.00 1.04 1.17 0.31 1.85 1.06 0.98 2.10 1.08 2.14 1.39 2.06 1.14 2.77 0.45 3.11 9.02 2.86 3.02
f1 (ppm)

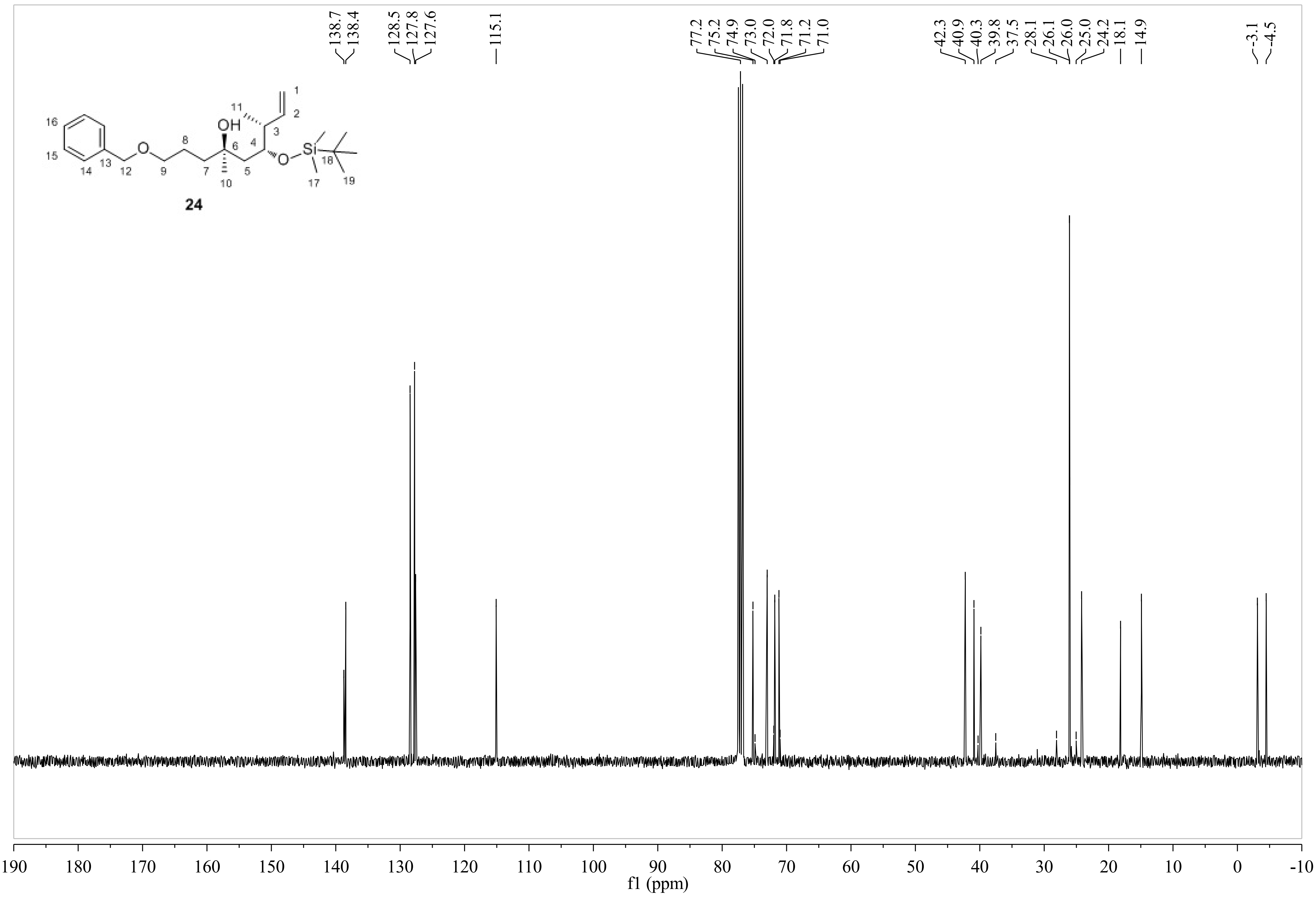

138.7
138.4
128.5
127.8
127.6
115.1
77.2
75.2
74.9
73.0
72.0
71.8
71.2
71.0
42.3
40.9
40.3
39.8
37.5
28.1
26.1
26.0
25.0
24.2
18.1
14.9
-3.1
-4.5
OH
O
Si
1
2
3
4
5
6
7
8
9
10
11
12
13
14
15
16
17
18
19
24
190
180
170
160
150
140
130
120
110
100
90
80
70
60
50
40
30
20
10
0
-10
f1 (ppm)

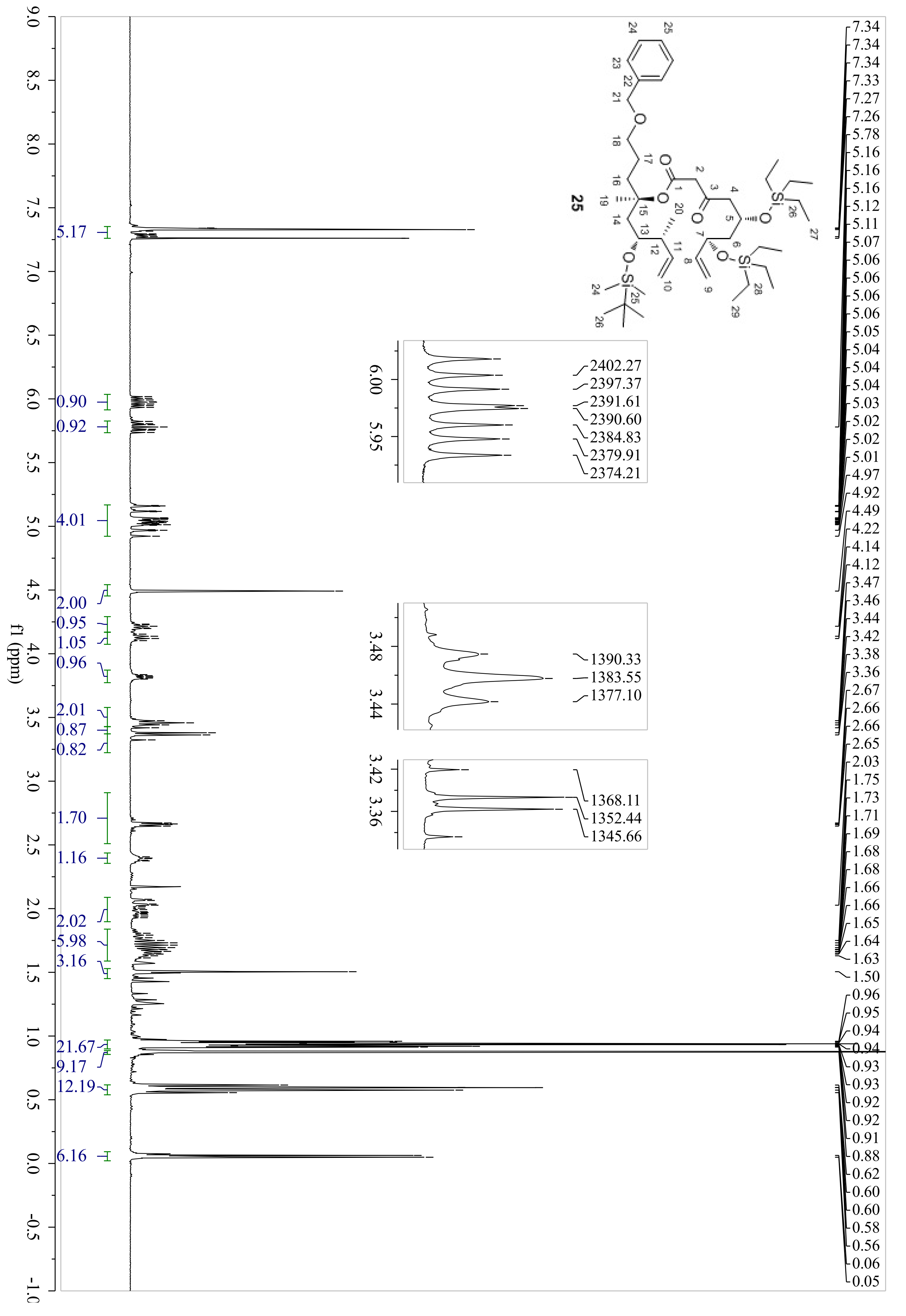

25
f1 (ppm)
9.0
8.5
8.0
7.5
7.0
6.5
6.0
5.5
5.0
4.5
4.0
3.5
3.0
2.5
2.0
1.5
1.0
0.5
0.0
-0.5
5.17
0.90
0.92
4.01
2.00
0.95
1.05
0.96
2.01
0.87
0.82
1.70
1.16
2.02
5.98
3.16
21.67
9.17
12.19
6.16
2402.27
2397.37
2391.61
2390.60
2384.83
2379.91
2374.21
6.00
5.95
1390.33
1383.55
1377.10
3.48
3.44
1368.11
1352.44
1345.66
3.42
3.36
7.34
7.34
7.34
7.33
7.27
7.26
5.78
5.16
5.16
5.16
5.12
5.11
5.07
5.06
5.06
5.06
5.06
5.05
5.04
5.04
5.04
5.03
5.02
5.02
5.01
4.97
4.92
4.49
4.22
4.14
4.12
3.47
3.46
3.44
3.42
3.38
3.36
2.67
2.66
2.66
2.65
2.03
1.75
1.73
1.71
1.69
1.68
1.68
1.66
1.66
1.65
1.64
1.63
1.50
0.96
0.95
0.94
0.94
0.93
0.93
0.92
0.92
0.91
0.88
0.62
0.60
0.60
0.58
0.56
0.06
0.05

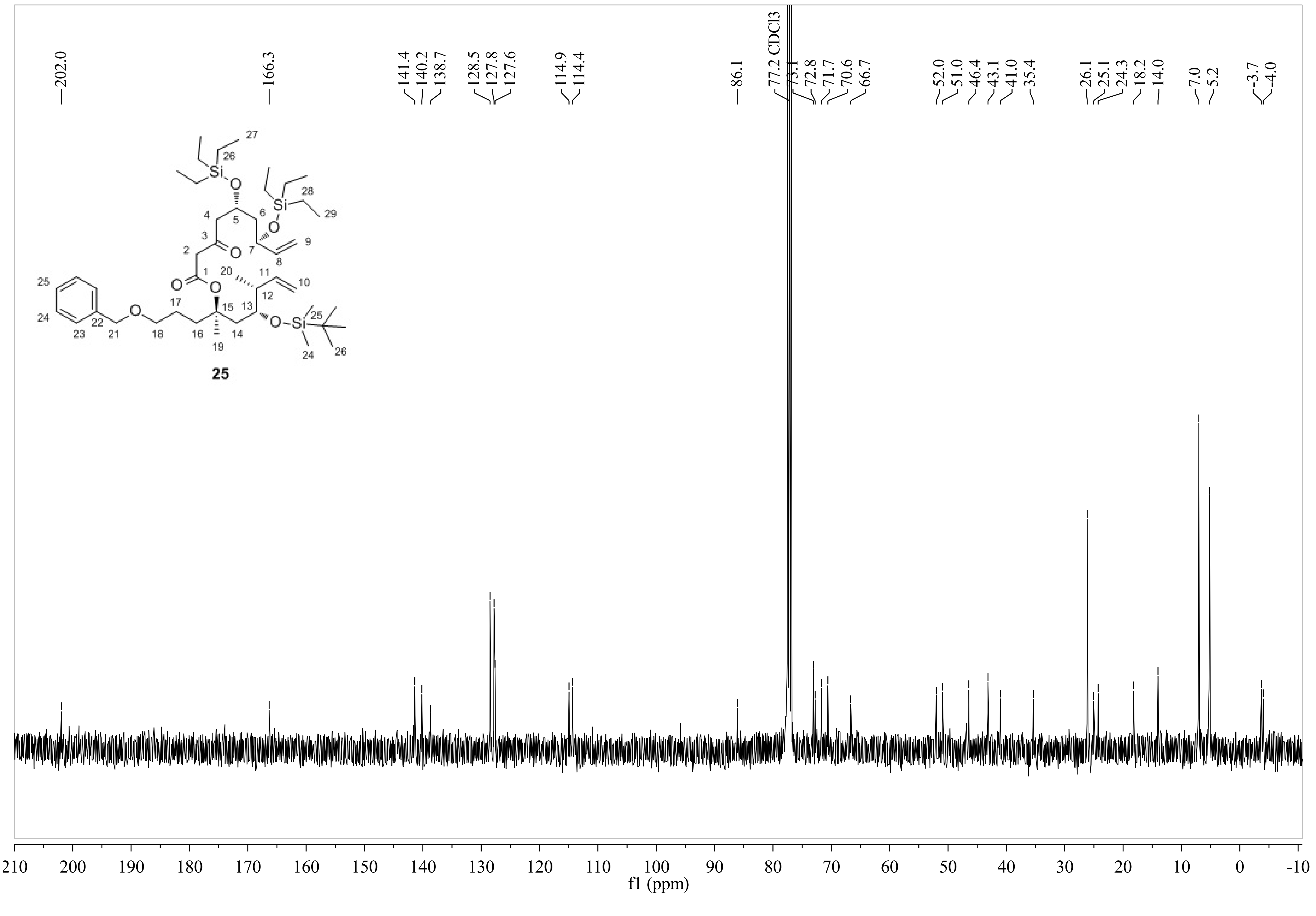
202.0
166.3
141.4
140.2
138.7
128.5
127.8
127.6
114.9
114.4
86.1
77.2 CDCl3
73.1
72.8
71.7
70.6
66.7
52.0
51.0
46.4
43.1
41.0
35.4
26.1
25.1
24.3
18.2
14.0
7.0
5.2
-3.7
-4.0
25
f1 (ppm)
210 200 190 180 170 160 150 140 130 120 110 100 90 80 70 60 50 40 30 20 10 0 -10

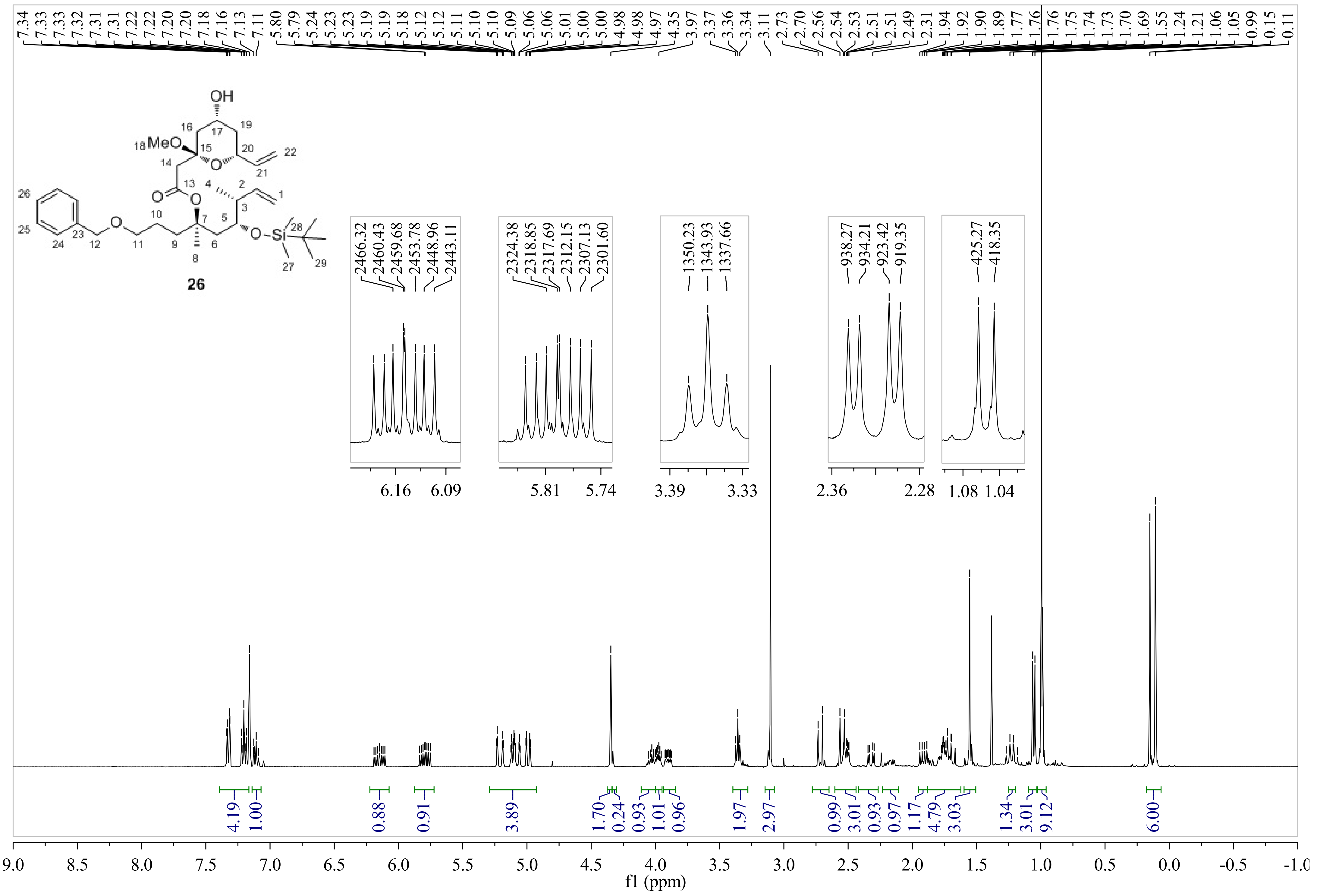
7.34 7.33 7.33 7.32 7.31 7.31 7.22 7.22 7.20 7.20 7.18 7.16 7.13 7.11 5.80 5.79 5.24 5.23 5.23 5.19 5.19 5.18 5.12 5.12 5.11 5.10 5.10 5.09 5.06 5.06 5.01 5.00 5.00 4.98 4.98 4.97 4.35 3.97 3.37 3.36 3.34 3.11 2.73 2.70 2.56 2.54 2.53 2.51 2.51 2.49 2.31 1.94 1.92 1.90 1.89 1.77 1.76 1.76 1.76 1.75 1.74 1.73 1.70 1.69 1.55 1.24 1.21 1.06 1.05 0.99 0.15 0.11
26
2466.32 2460.43 2459.68 2453.78 2448.96 2443.11
6.16 6.09
2324.38 2318.85 2317.69 2312.15 2307.13 2301.60
5.81 5.74
1350.23 1343.93 1337.66
3.39 3.33
938.27 934.21 923.42 919.35
2.36 2.28
425.27 418.35
1.08 1.04
4.19 1.00 0.88 0.91 3.89 1.70 0.24 0.93 1.01 0.96 1.97 2.97 0.99 3.01 0.93 0.97 1.17 4.79 3.03 1.34 3.01 9.12 6.00
9.0 8.5 8.0 7.5 7.0 6.5 6.0 5.5 5.0 4.5 4.0 3.5 3.0 2.5 2.0 1.5 1.0 0.5 0.0 -0.5 -1.0
f1 (ppm)

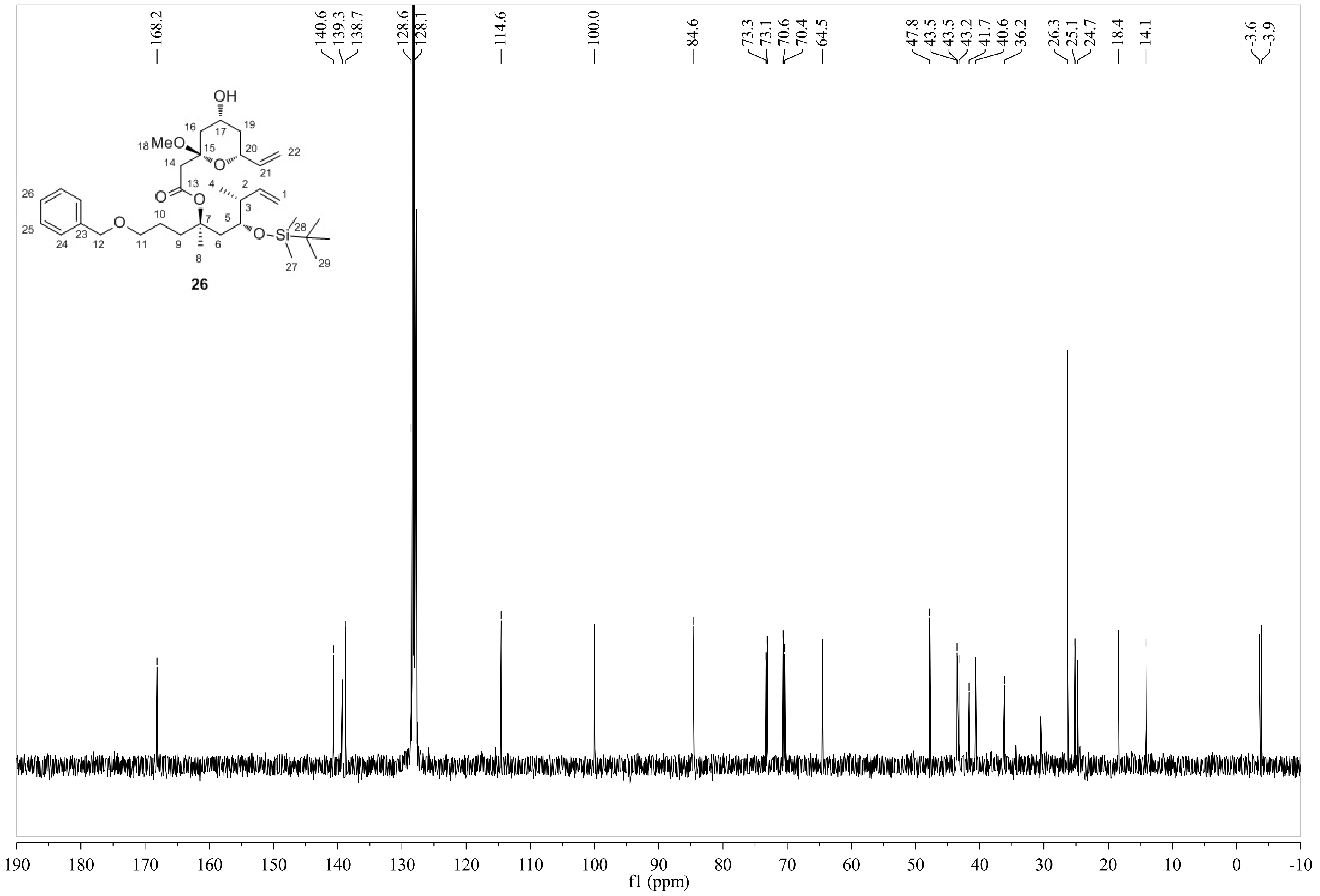

26
168.2
140.6
139.3
138.7
128.6
128.1
114.6
100.0
84.6
73.3
73.1
70.6
70.4
64.5
47.8
43.5
43.5
43.2
41.7
40.6
36.2
26.3
25.1
24.7
18.4
14.1
-3.6
-3.9
190
180
170
160
150
140
130
120
110
100
90
80
70
60
50
40
30
20
10
0
-10
f1 (ppm)

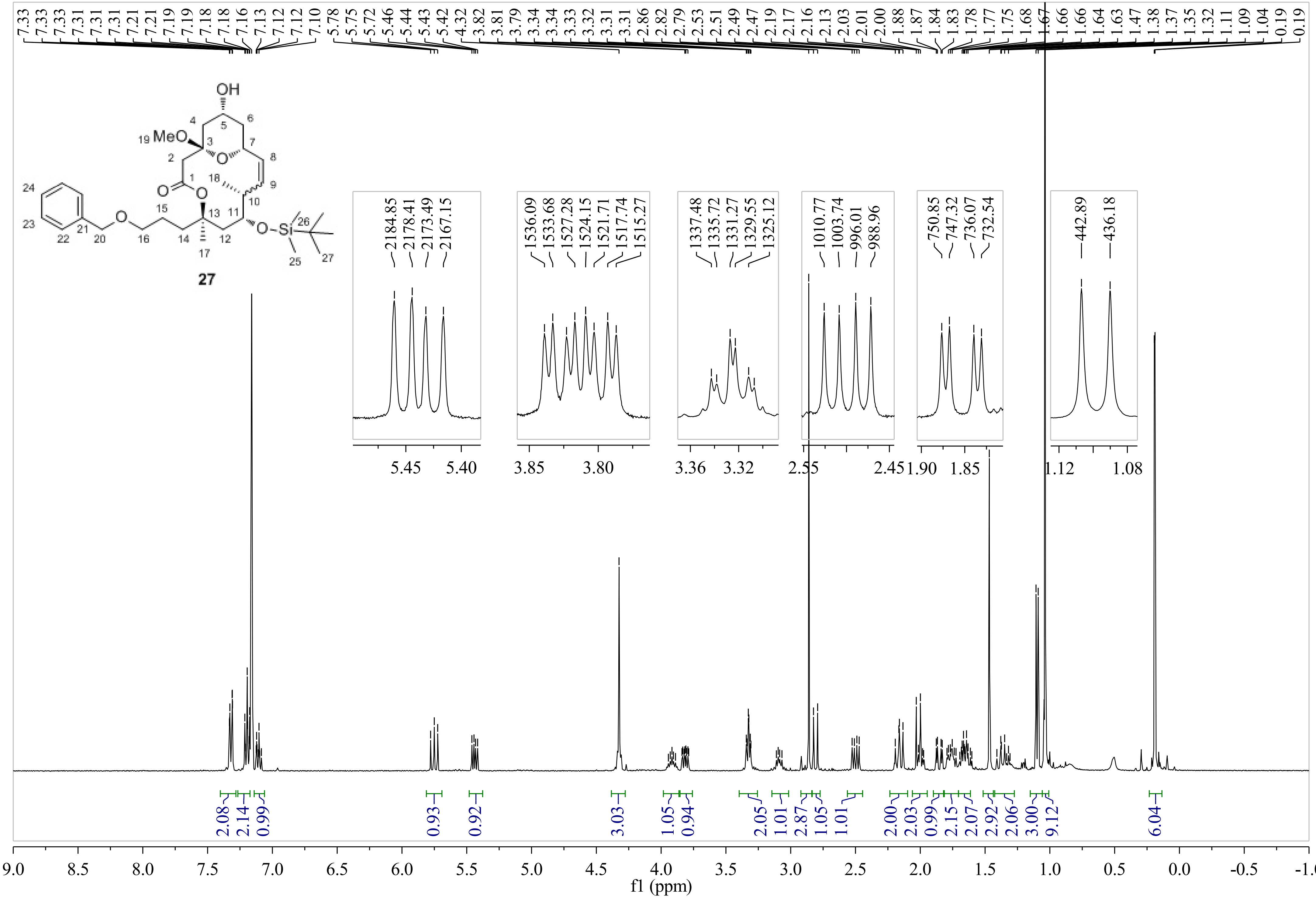

7.33 7.33 7.33 7.31 7.31 7.31 7.21 7.21 7.19 7.19 7.18 7.18 7.16 7.13 7.12 7.12 7.10 5.78 5.75 5.72 5.46 5.44 5.43 5.42 4.32 3.82 3.81 3.79 3.34 3.34 3.33 3.32 3.31 3.31 2.86 2.82 2.79 2.53 2.51 2.49 2.47 2.19 2.17 2.16 2.13 2.03 2.01 2.00 1.88 1.87 1.84 1.83 1.78 1.77 1.75 1.68 1.67 1.66 1.66 1.64 1.63 1.47 1.38 1.37 1.35 1.32 1.11 1.09 1.04 0.19 0.19
OH
MeO
27
2184.85 2178.41 2173.49 2167.15
1536.09 1533.68 1527.28 1524.15 1521.71 1517.74 1515.27
1337.48 1335.72 1331.27 1329.55 1325.12
1010.77 1003.74 996.01 988.96
750.85 747.32 736.07 732.54
442.89 436.18
5.45 5.40 3.85 3.80 3.36 3.32 2.55 2.45 1.90 1.85 1.12 1.08
2.08 2.14 0.99 0.93 0.92 3.03 1.05 0.94 2.05 1.01 2.87 1.05 1.01 2.00 2.03 0.99 2.15 2.07 2.92 2.06 3.00 9.12 6.04
9.0 8.5 8.0 7.5 7.0 6.5 6.0 5.5 5.0 4.5 4.0 3.5 3.0 2.5 2.0 1.5 1.0 0.5 0.0 -0.5 -1.0
f1 (ppm)

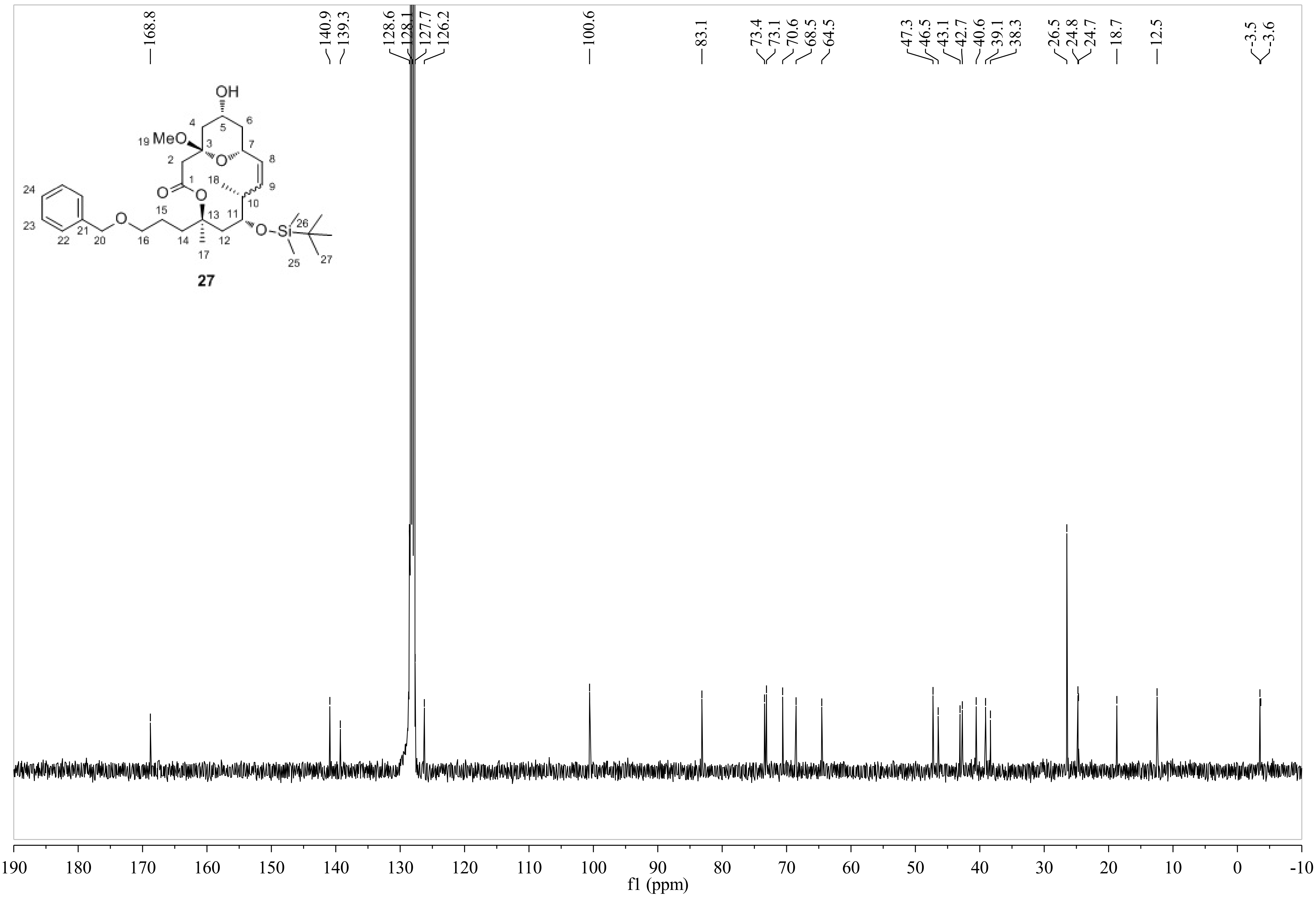
168.8
140.9
139.3
128.6
128.1
127.7
126.2
100.6
83.1
73.4
73.1
70.6
68.5
64.5
47.3
46.5
43.1
42.7
40.6
39.1
38.3
26.5
24.8
24.7
18.7
12.5
-3.5
-3.6
OH
MeO
O
O-Si
27
190
180
170
160
150
140
130
120
110
100
90
80
70
60
50
40
30
20
10
0
-10
f1 (ppm)

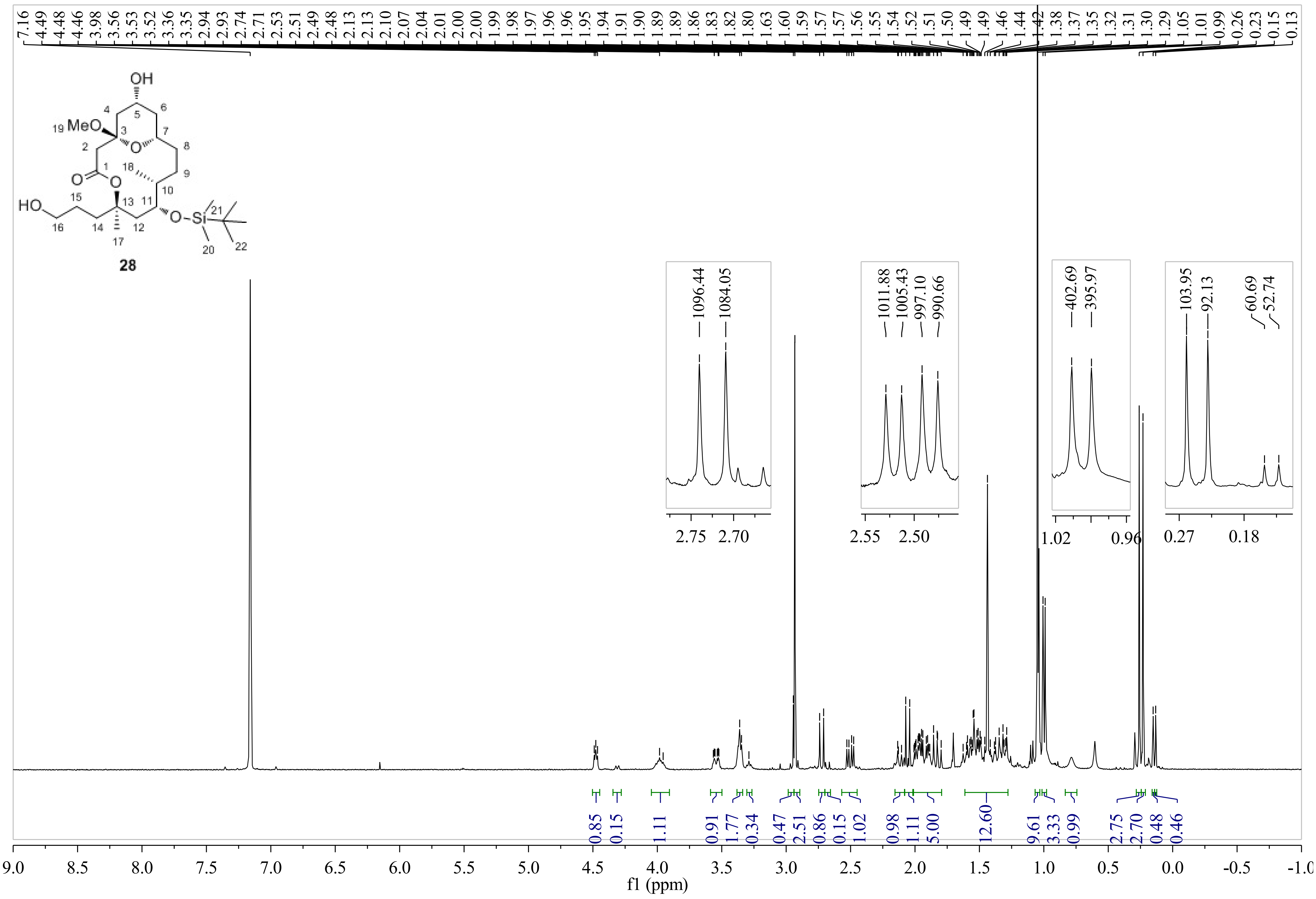
7.16 4.49 4.48 4.46 3.98 3.56 3.53 3.52 3.36 3.35 2.94 2.93 2.74 2.71 2.53 2.51 2.49 2.48 2.13 2.13 2.10 2.07 2.04 2.01 2.00 2.00 1.99 1.98 1.97 1.96 1.96 1.95 1.94 1.91 1.90 1.89 1.89 1.86 1.83 1.82 1.80 1.63 1.60 1.59 1.57 1.57 1.56 1.55 1.54 1.52 1.51 1.50 1.49 1.49 1.46 1.44 1.42 1.38 1.37 1.35 1.32 1.31 1.30 1.29 1.05 1.01 0.99 0.26 0.23 0.15 0.13
OH
19 MeO
28
HO
O-Si
1096.44
1084.05
2.75 2.70
1011.88
1005.43
997.10
990.66
2.55 2.50
402.69
395.97
1.02 0.96
103.95
92.13
60.69
52.74
0.27 0.18
0.85 0.15 1.11 0.91 1.77 0.34 0.47 2.51 0.86 0.15 1.02 0.98 1.11 5.00 12.60 9.61 3.33 0.99 2.75 2.70 0.48 0.46
9.0 8.5 8.0 7.5 7.0 6.5 6.0 5.5 5.0 4.5 4.0 3.5 3.0 2.5 2.0 1.5 1.0 0.5 0.0 -0.5 -1.0
f1 (ppm)

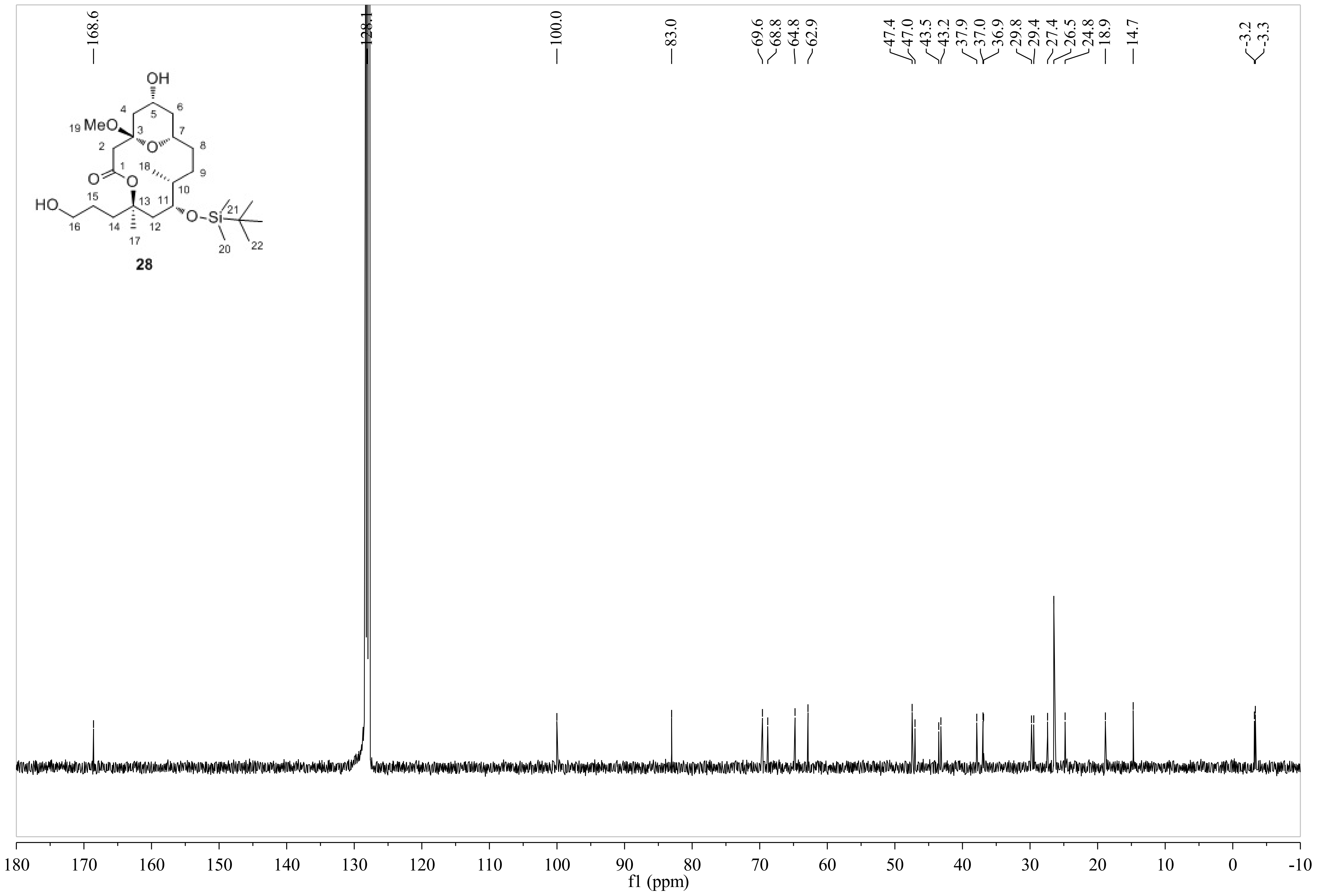
168.6
128.1
100.0
83.0
69.6
68.8
64.8
62.9
47.4
47.0
43.5
43.2
37.9
37.0
36.9
29.8
29.4
27.4
26.5
24.8
18.9
14.7
-3.2
-3.3
OH
MeO
HO
O-Si
28
180 170 160 150 140 130 120 110 100 90 80 70 60 50 40 30 20 10 0 -10
f1 (ppm)

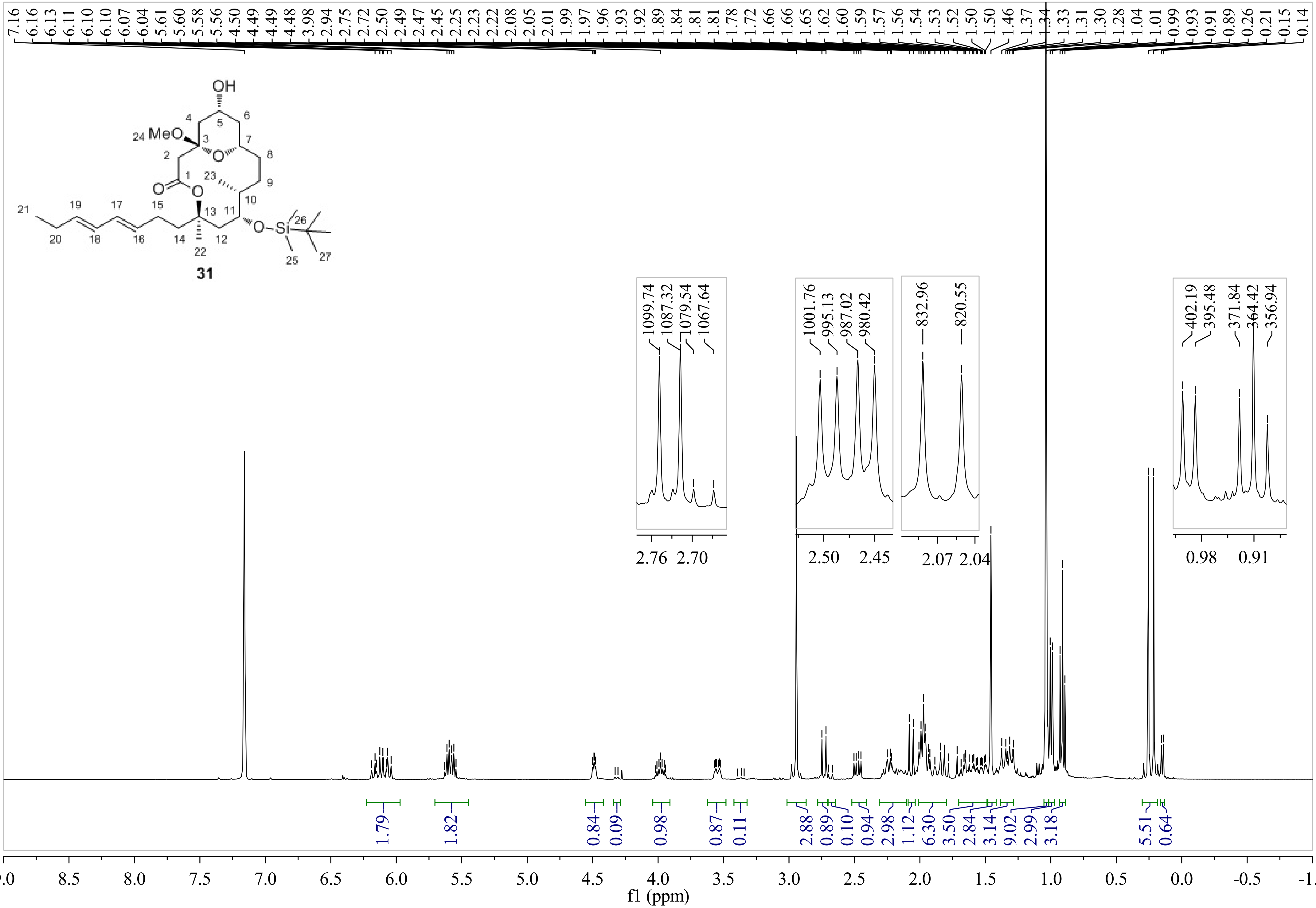

7.16 6.16 6.13 6.11 6.10 6.10 6.07 6.04 5.61 5.60 5.58 5.56 4.50 4.49 4.49 4.48 3.98 2.94 2.75 2.72 2.50 2.49 2.47 2.45 2.25 2.23 2.22 2.08 2.05 2.01 1.99 1.97 1.96 1.93 1.92 1.89 1.84 1.81 1.81 1.78 1.72 1.66 1.66 1.65 1.62 1.60 1.59 1.57 1.56 1.54 1.53 1.52 1.50 1.50 1.46 1.37 1.34 1.33 1.31 1.30 1.28 1.04 1.01 0.99 0.93 0.91 0.89 0.26 0.21 0.15 0.14
OH
MeO
O
O
O
O–Si
1 2 3 4 5 6 7 8 9 10 11 12 13 14 15 16 17 18 19 20 21 22 23 24 25 26 27
31
1099.74
1087.32
1079.54
1067.64
2.76 2.70
1001.76
995.13
987.02
980.42
2.50 2.45
832.96
820.55
2.07 2.04
402.19
395.48
371.84
364.42
356.94
0.98 0.91
1.79 1.82 0.84 0.09 0.98 0.87 0.11 2.88 0.89 0.10 0.94 2.98 1.12 6.30 3.50 2.84 3.14 9.02 2.99 3.18 5.51 0.64
9.0 8.5 8.0 7.5 7.0 6.5 6.0 5.5 5.0 4.5 4.0 3.5 3.0 2.5 2.0 1.5 1.0 0.5 0.0 -0.5 -1.0
f1 (ppm)

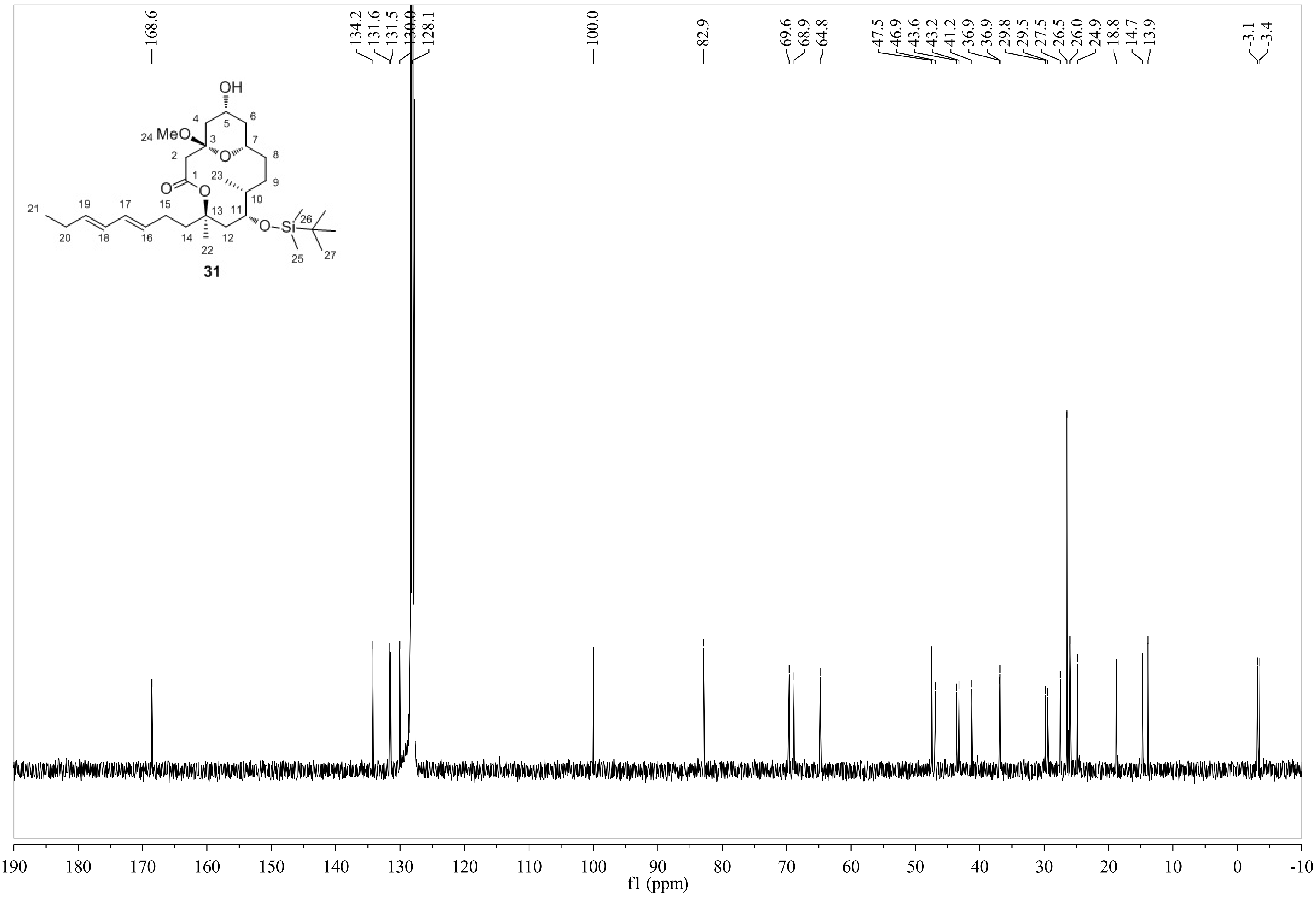
168.6
134.2
131.6
131.5
130.0
128.1
100.0
82.9
69.6
68.9
64.8
47.5
46.9
43.6
43.2
41.2
36.9
36.9
29.8
29.5
27.5
26.5
26.0
24.9
18.8
14.7
13.9
-3.1
-3.4
OH
MeO
O
31
190 180 170 160 150 140 130 120 110 100 90 80 70 60 50 40 30 20 10 0 -10
f1 (ppm)

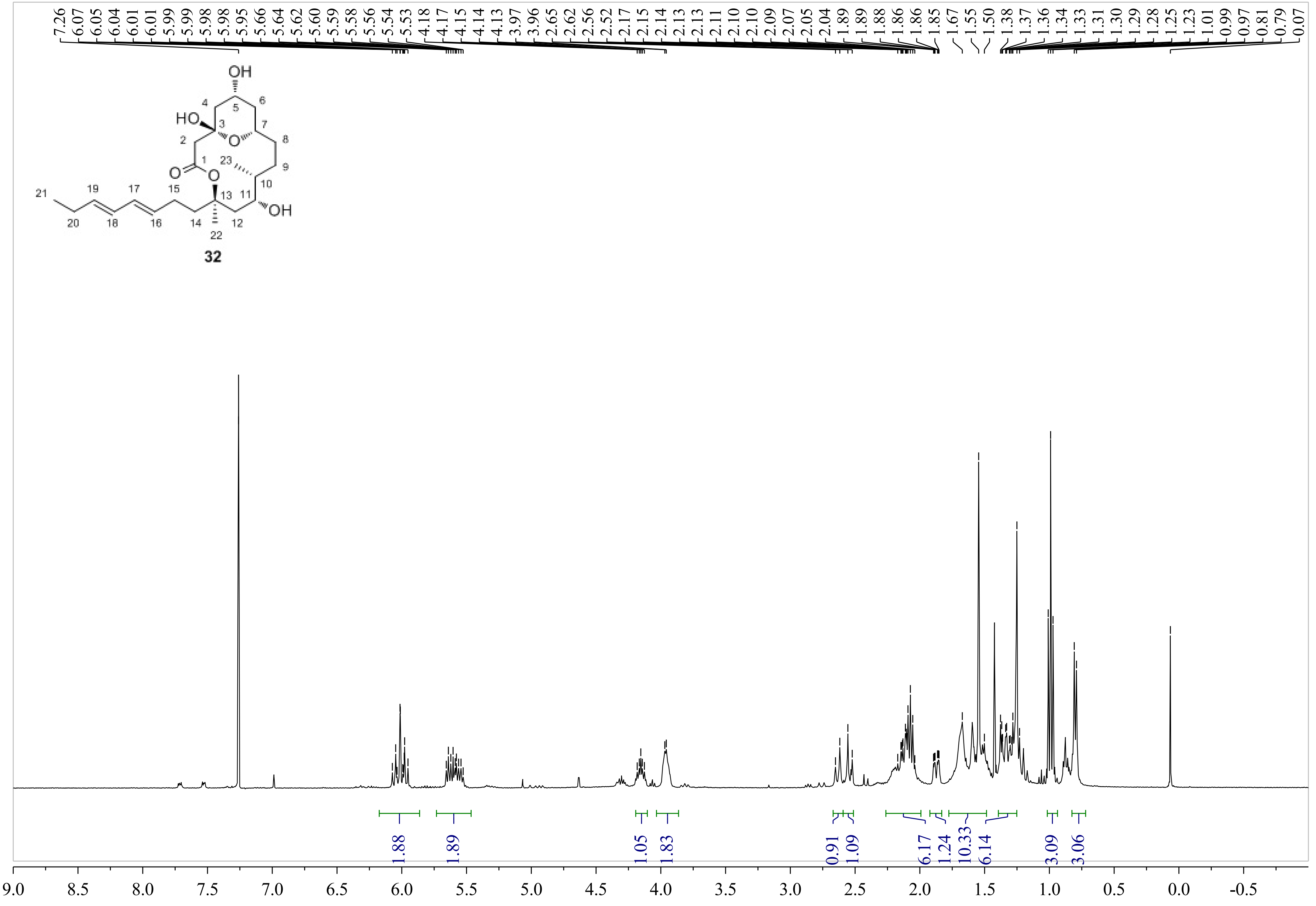

7.26
6.07
6.05
6.04
6.01
6.01
5.99
5.99
5.98
5.98
5.95
5.66
5.64
5.62
5.60
5.59
5.58
5.56
5.54
5.53
4.18
4.17
4.15
4.14
4.13
3.97
3.96
2.65
2.62
2.56
2.52
2.17
2.15
2.14
2.13
2.13
2.11
2.10
2.10
2.09
2.07
2.05
2.04
1.89
1.89
1.88
1.86
1.86
1.85
1.67
1.55
1.50
1.38
1.37
1.36
1.34
1.33
1.31
1.30
1.29
1.28
1.25
1.23
1.01
0.99
0.97
0.81
0.79
0.07
OH
HO
O
OH
32
1.88
1.89
1.05
1.83
0.91
1.09
6.17
1.24
10.33
6.14
3.09
3.06
9.0
8.5
8.0
7.5
7.0
6.5
6.0
5.5
5.0
4.5
4.0
3.5
3.0
2.5
2.0
1.5
1.0
0.5
0.0
-0.5

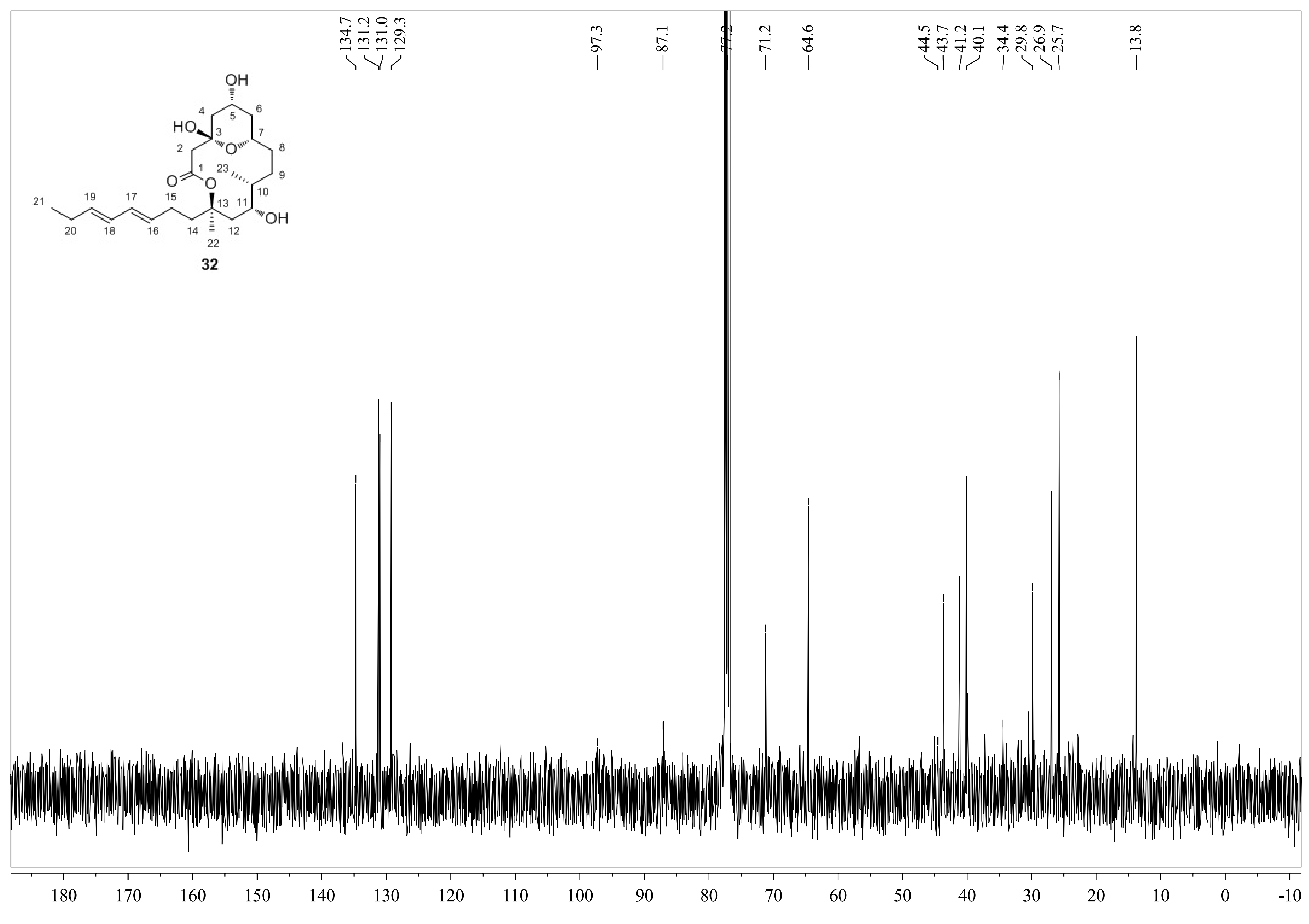

134.7
131.2
131.0
129.3
97.3
87.1
77.2
71.2
64.6
44.5
43.7
41.2
40.1
34.4
29.8
26.9
25.7
13.8
32
180
170
160
150
140
130
120
110
100
90
80
70
60
50
40
30
20
10
0
-10
